# Emotion Differentiation in Adolescents: Short-term Trade-offs with Regulation Variability and Emotion Intensity

**DOI:** 10.1007/s42761-025-00301-4

**Published:** 2025-05-28

**Authors:** Tak Tsun Lo, Maaike Verhagen, J. Loes Pouwels, Eeske van Roekel, Sarah T. O’Brien, Gillian Debra, Jolien Braet, Jacqueline M. Vink, Dominique F. Maciejewski

**Affiliations:** 1 Behavioural Science Institute, Radboud University, Nijmegen, The Netherlands; 2 School of Social and Behavioral Sciences, Tilburg University, Tilburg, The Netherlands; 3 Tilburg Experience Sampling Center, Tilburg University, Warandelaan 2, Tilburg, 5037 AB The Netherlands; 4 Melbourne School of Psychological Sciences, University of Melbourne, Melbourne, Australia; 5 Faculty of Psychology and Educational Sciences, Ghent University, Ghent, Belgium

**Keywords:** Dynamics, Emotion differentiation, Emotion regulation variability, Emotion intensity, Adolescents

## Abstract

Emotion differentiation—distinctively labeling emotions—is theorized to guide adolescents in regulating emotions amid changing daily life situations. Momentary fluctuations in emotion differentiation are expected to introduce variability in using emotion regulation strategies, leading to sequential emotion intensity changes. Using five experience sampling datasets (*N* = 750, aged 11–25, 59.17% female, 25,834 observations) that repeatedly assess emotion differentiation and emotion regulation variability, we examined their interaction and impact on emotion intensity. Surprisingly, moments of heightened emotion differentiation were followed by more stable use of regulation strategies (lower variability), while moments of higher emotion regulation variability were followed by less emotion differentiation. Both heightened differentiation and regulation variability preceded contra-hedonic outcomes, such as increased negative emotions and decreased positive emotions. These findings were robust across different types of emotion regulation variability (intensity or switching) and valences of emotions (positive or negative). In the short term, emotion differentiation predicts reduced regulation variability and may bring unpleasant changes in emotion intensity.

**Supplementary Information:**

The online version contains supplementary material available at 10.1007/s42761-025-00301-4.

Adolescence^1^ is a period of emotional challenges ranging from pubertal changes, academic or work-related pressure, and transforming interpersonal relationships (Holmbeck et al., 2006). To navigate this transitional period, adolescents use various strategies to regulate the intensity of their emotions (Klein et al., 2022). Emotion differentiation—how well emotions are distinctively labelled—is expected to facilitate emotion regulation, because knowing what one feels informs ways of regulating one’s emotions (Barrett et al.., 2001; Berking et al., 2014; Schwarz & Clore, 1983). Based on this assumption, fluctuating levels of emotion differentiation within an adolescent should introduce subsequent variability in the use of emotion regulation strategies and, sequentially, changes in emotion intensity (Kashdan et al., 2015). In the background of these theoretical views, there are increasing interests to develop self-guided and online interventions that target emotion differentiation for improving emotion regulation (Matt et al., 2024; Seah & Coifman, 2024; Van der Gucht et al., 2019). Before it becomes appropriate to target emotion differentiation in interventions, we need to clarify the two theorized effects of emotion differentiation on emotion regulation variability and emotion intensity changes in adolescents’ daily lives, which remain empirically understudied. Therefore, this study aims to investigate the temporal sequences between adolescents’ emotion differentiation and emotion regulation variability in their daily lives, and the subsequent changes in emotion intensity therein.

**Fig. 1:**
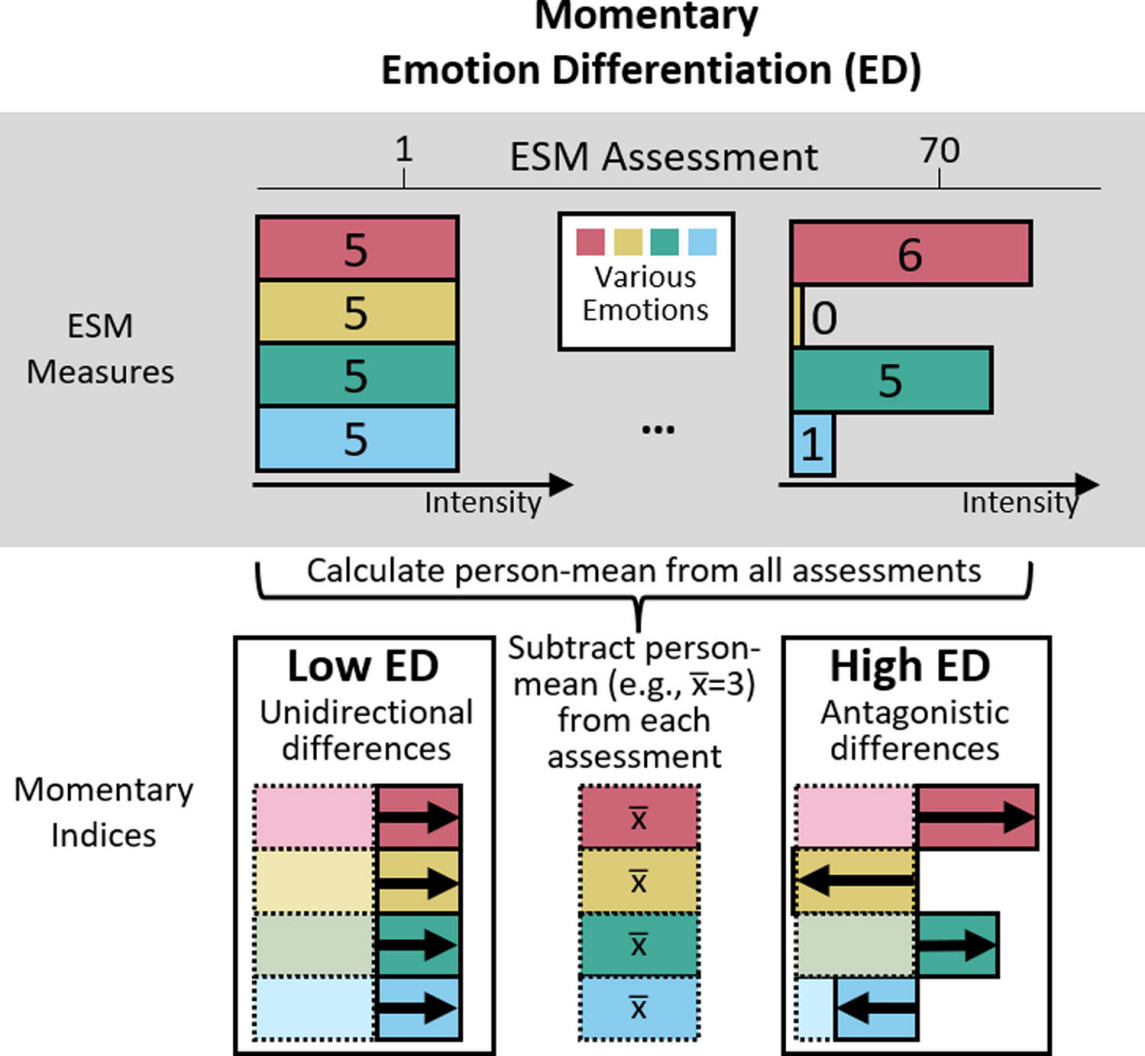
Hypothetical assessments of ESM measures to illustrate how to calculate emotion differentiation. For simplicity, only the calculation steps of the numerator but not the denominator are shown. Numbers on the bars represent the intensity ratings of emotions

## Is Emotion Differentiation Related to Emotion Regulation Strategy Use in Daily Life?

To study the relation between emotion differentiation and emotion regulation in daily life, researchers often assess emotions and emotion regulation strategies repeatedly over the course of several days, for instance using daily diaries or experience sampling methods (ESM). These methods have two advantages, namely capturing life as it is lived with high ecological validity, and allowing researchers to tease apart within-person fluctuations from individual differences of the baseline emotion (regulation) throughout the assessments (Bolger & Laurenceau, 2013).

Using these methods, researchers have investigated how emotion differentiation is related to emotion regulation strategy use. Two studies that investigated this association between individuals gave an inconclusive picture. One daily diary study found that individuals with higher differentiation of negative emotions showed greater average use of emotion regulation strategies compared to those with lower emotion differentiation (Barrett et al.., 2001), but an ESM study that examined separate strategies found that high differentiators used less social sharing compared to low differentiators (Kalokerinos et al., 2019). Additionally, this ESM study revealed no significant associations between emotion differentiation and five other strategies examined (e.g., distraction).

ESM allows researchers to scrutinize daily life within-person fluctuations of emotions and their regulation. However, similar to studies on individual differences, there lacks empirical evidence on within-person temporal relations from emotion differentiation to emotion regulation strategy use. A 10-day ESM study showed that on days when university students had higher negative emotion differentiation than usual, they did not use their emotion regulation strategies any differently compared to their average use (O’Toole et al., 2021). Using a recently developed within-person momentary emotion differentiation index (Fig. 1, Erbas et al., 2021), one study tested if emotion differentiation preceded emotion regulation: Lower emotion differentiation predicted subsequent higher social sharing. However, this finding was only seen in two out of four datasets analyzed (Sels et al., 2024). Overall, empirical evidence suggests weak between-person associations between emotion differentiation and the use of separate emotion regulation strategies, and potentially no concurrent or temporal within-person associations.

**Fig. 2:**
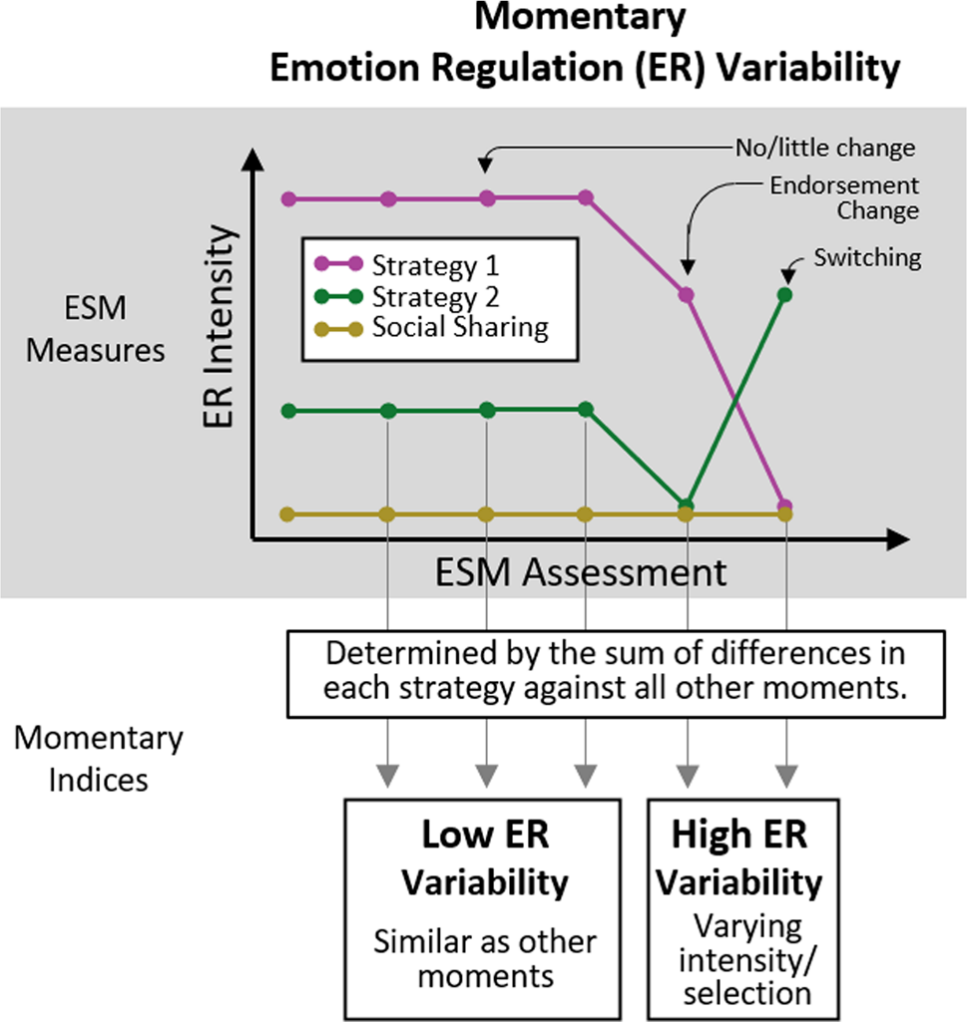
Hypothetical assessments of ESM measures to illustrate how to calculate emotion regulation variability. For simplicity, only the calculation steps of the numerator but not the denominator are shown

## The Need To Consider Variability of Multiple Emotion Regulation Strategies Collectively

These weak associations may have been a result of a methodological limitation, namely analyzing the variability of emotion regulation strategies separately. Hypothetically, imagine an adolescent who consistently not used social sharing but alternated between using two other strategies throughout all measurements (Fig. 2). Researchers who only analyze the adolescent’s social sharing would, by their decision of analyzing a single strategy, miss out variability from the two other strategies. Simulation studies have demonstrated the poor performance of this approach of single-strategy analysis in detecting emotion regulation variability, even if the approach is mitigated by taking the average variability from multiple single-strategy analyses (Lo et al., 2024). Therefore, emotion regulation variability should be considered between multiple strategies *collectively* across time. A recently validated method for capturing emotion regulation variability is the Bray-Curtis dissimilarity index, which has been commonly used in ecological research to quantify compositional changes in multiple species over sites. Applied to emotion regulation, treating each ESM assessment as a site and regulation strategies as species, Bray-Curtis dissimilarity denotes the degree to which use of strategies at a moment of interest is different from other moments. The Bray-Curtis dissimilarity full index can be partitioned into two subcomponents that reflect two theoretical grounded processes of emotion regulation variability: Strategy switching (e.g., replacing one strategy with another) and endorsement change (e.g., decreasing the extent of emotion regulation). Bray-Curtis dissimilarity has an advantage over conventional variability indices (e.g., standard deviation) in the detection of momentary within-person emotion regulation variability in all strategies (Lo et al., 2024). This momentary index can be averaged within a person. Such trait-like emotion regulation variability is theorized to be the foundation of adaptively using emotion regulation strategies to match situational demands (Aldao et al., 2015). To overcome the previous methodological limitation of examining strategies separately, we reexamined whether emotion differentiation affects the subsequent use of multiple emotion regulation strategies using the Bray-Curtis dissimilarity index.

## Changes in Emotion Intensity: Feeling Better or Worse?

Adolescents endorse emotion regulation strategies to change the perceived intensity of emotions. If emotion differentiation is to facilitate emotion regulation, a subsequent change in emotion intensity should follow. Typically, emotion regulation is assumed to produce pro-hedonic outcomes—decreasing negative emotions and increasing positive emotions (Webb et al., 2012). Evidence supports this pro-hedonic effect in both individual differences and within-person fluctuations in emotion differentiation. For individual differences, adolescents with high emotion differentiation appear buffered from depressive feelings when experiencing stress (Nook, Flournoy et al., 2021) or rumination (Starr et al., 2017). At the within-person level, momentary emotion differentiation is positively associated with simultaneous pro-hedonic outcomes (Erbas et al., 2021). Emotion regulation strategies also play a role in the differentiation-intensity link; individuals with higher baseline negative emotion differentiation can reduce negative emotions with less strategy deployment compared to those with lower differentiation (Kalokerinos et al., 2019). Integrating this evidence with theoretical models of emotion differentiation (Kashdan et al., 2015), we test a within-person mediation model, where emotion differentiation influences emotion intensity change through emotion regulation.

Building on previous findings, one might speculate that heightened emotion differentiation and emotion regulation variability would lead to pro-hedonic changes in emotion intensity (i.e., decreases in negative emotions and increases in positive emotions). However, it is equally plausible that contra-hedonic outcomes—such as increases in negative emotions and decreases in positive emotions—could result instead. Psychotherapy literature recognizes that emotion differentiation, a common therapeutic task across different treatment approaches (Sønderland et al., 2023), intensifies negative emotions elicited in therapy (Lane et al., 2022). Similar short-term contra-hedonic outcomes appear in non-clinical samples. In one experiment with university students who feared spiders, participants assigned to a condition that put their feelings into words—a procedure related to the increase of baseline emotion differentiation (Seah & Coifman, 2024)—demonstrated reduced physiological fear arousal and improved approach behaviors only after a week, but not immediately (Kircanski et al., 2012). In another experiment, students who explored their emotions in previous upsetting experiences and wrote about them showed immediate spikes in negative emotion (Pascual-Leone et al., 2016). Complicating the expectation of pro- or contra-hedonic outcomes, an ESM study showed that attention to emotion is associated with high negative emotion intensity concurrently but preceded subsequent decreases in intensity (Thompson et al., 2011); Assuming attending to emotions is a prerequisite for emotion differentiation, the contribution of emotion differentiation to subsequent changes in emotion intensity likely depends on how long attention is sustained.

Based on this evidence, we remained open to both short-term pro-hedonic and contra-hedonic changes in emotion intensity when examining whether emotion differentiation affects subsequent emotion intensity via emotion regulation variability.

## The Present Study

Our study tested the temporal relations between emotion differentiation and emotion regulation variability, and their effect on subsequent emotion intensity within adolescents. In all our analyses, we focused solely on negative emotion regulation strategies, as there were limited datasets available that measured positive emotion regulation strategies, preventing us from testing similar hypotheses with sufficient statistical power.

In line with the idea that emotion differentiation facilitates emotion regulation, which we expected to lead to changes in strategy use (i.e., increases in variability), we pre-registered three hypotheses: Hypothesis 1 states that, within an adolescent, greater emotion differentiation at a given moment is related to higher emotion regulation variability at the subsequent moment. Previous theoretical discussions did not expect a reversed temporal sequence (Kashdan et al., 2015; Thompson et al., 2021). Therefore, Hypothesis 2 states that, within an adolescent, emotion regulation variability at one moment is not associated with emotion differentiation at the following moment. Hypothesis 3 is between-person, stating that adolescents with higher emotion differentiation would show higher emotion regulation variability on average. After analyzing the results from these hypotheses, we formulated the following exploratory research questions: Research question 1 explores whether within-person fluctuations in emotion differentiation and emotion regulation variability precede subsequent pro-/contra-hedonic changes in emotion intensity. Additionally, research question 2 explores if the differentiation-intensity temporal relation, if any, is mediated by emotion regulation variability.

**Table 1:** Overview of study characteristics of included datasets

	G(F)ood together (Verhagen et al., 2022)	Emotions in daily life 2011 (Koval et al., 2013)	3-wave longitudinal study (Erbas et al., 2018)	Emotions in daily life (Van Roekel & Trompetter, 2023)	Outside-in (Braet et al., 2023)
Institute	Radboud University, the Netherlands	KU Leuven, Belgium	KU Leuven, Belgium	Tilburg University, the Netherlands	Ghent University, Belgium
N after exclusion criteria applied	83	97	202	178	218
Age *M* *(SD)*, range	16.4 (0.7),15.0–18.0	19.1 (1.3),18.0–24.0	18.3 (1),17.0–24.0	20.9 (1.7),18.0–25.0	13.5 (0.6),11.0–15.0
Female	57%	63%	55%	78%	48%
Observations per day	10	10	10	5	5
Number of days	7	7	7	14	14
Interval scheme	Semi-random	Stratified-random	Stratified-random	Quasi-random	Fixed
Positive emotions	4 items:ContentRelaxedJoyfulEnergetic	2 items:RelaxedHappy	3 items:HappyRelaxedCheerful	7 items:EnthusiasticContentEnergeticCalmDeterminedCheerfulGrateful	3 items:HappyCalmEnthusiastic
Negative emotions	5 items:IrritatedWorriedDepressedInsecureLonely	4 items:AngryAnxiousDepressedSad	6 items:AngryAnxiousDepressedSadLonelyStress	6 items:AngryIrritatedDepressed SadNervousBored	6 items:AngryInsecureAfraidSadStressedBored
Emotion regulation strategies	5 items:RuminationReappraisalSuppressionAcceptanceSocial Sharing	6 items:RuminationReappraisalDistraction ReflectionSuppressionSocial Sharing	6 items:RuminationReappraisalDistractionWorrySuppressionSocial Sharing	7 items:RuminationDistractionAvoidanceProblem SolvingAcceptanceCo-BroodingSocial Sharing	8 items:RuminationReappraisalDistractionSelf-Compassion (Support)Self-compassion (Cheer-up)ExpressionSuppressionSocial Sharing

All pre-registered hypotheses and research questions concerned differentiation of *negative* emotions because previous literature mostly investigated negative emotion differentiation. As part of our sensitivity analyses, we repeated testing all hypotheses and research questions with positive emotion differentiation and two subcomponents of emotion regulation variability. These sensitivity analyses served to enrich our understanding on these understudied specifications (positive emotion differentiation and emotion regulation variability subcomponents). We tested all these hypotheses using data from five ESM studies, in which adolescents rated momentary emotions and emotion regulation strategies multiple times per day.

## Method

This paper follows the Workflow for Open Reproducible Code in Science (Van Lissa et al., 2021). The pre-registration (hypotheses and analysis plan), data, and analysis codes of this study are available via https://osf.io/cq6n4/. In Supplemental Materials 1, we detailed our *a priori* power analysis which showed we had more than 80% power to test our confirmatory hypotheses and exploratory research question 1, and reported four minor deviations we had from our pre-registration.

### Participants and Procedures

This study combines five ESM datasets (see Supplemental Materials 2 for details on participants and procedures). Table 1 shows an overview of the demographics per dataset. The five datasets included participants with a mean age of 17.42 years (*SD* = 2.99; range: 11 to 25 years), with 59.17% females (range across datasets: 47.71 to 77.59%). All studies, approved by respective ethical committees, were conducted in Belgium and the Netherlands with Dutch-speaking participants. All studies assessed participants either 10 times for 7 days or 5 times for 14 days, resulting in the same 70 observations. As pre-registered, we excluded 33 participants with zero variance in positive emotions, negative emotions, or emotion regulation strategies. We further excluded 4 participants with an average reaction time below 500 ms because it may indicate careless responding (McCabe, 2012). Participants completed on average 74% of all possible observations (*SD* = 23%). Supplemental Materials 2 has further details on participants and procedures of all datasets.

### Measures

#### ESM Measures

The studies differed in how many items were used to assess negative emotions, positive emotions, and emotion regulation strategies, but they all used multiple items with unipolar scales (see Table 1). Within each dataset, all items were rescaled before analyses to a scale of 0 to 10 to facilitate pooling across studies. Within-person correlations of items in the same scales were all lower than.80 (Supplemental Materials 3), indicating no multicollinearity problem (Katz, 2006; see the application to an ESM study, Wang et al., 2024). Intraclass correlation coefficients (ICC) of all items ranged from 0.19 to 0.64, indicating they had adequate within-person variance for further analyses. Supplemental Materials 2 has full item wordings for all items and the steps we have taken to assess their reliability and validity.

#### Momentary Indices Calculated from ESM Measures

##### Intensity of Positive Emotions, Negative Emotions, and Emotion Regulation

We calculated momentary intensities of negative emotions, positive emotions, and emotion regulation as the mean intensities of relevant items (e.g., in dataset 2, momentary negative emotion intensity is the mean of *angry*, *sad*, *anxious*, and *depressed*). Multi-level confirmatory factor analyses using the *lavaan* package (Rosseel, 2012) showed positive and negative emotions loaded separately on two factors as indicated with satisfactory fit indices (Supplemental Materials 4). Reliability was satisfactory for all indices within adolescents (positive emotion intensity: .60 to .80; negative emotion intensity: .66 to .76; emotion regulation intensity: .52 to .72) and between adolescents (positive emotion intensity: .88 to .93; negative emotion intensity: .90 to .94; emotion regulation intensity: .68 to .97).

##### Emotion Differentiation

To assess the degree of positive and negative emotion differentiation within adolescents at a specific moment, we calculated the momentary emotion differentiation index from the positive and negative emotion items (Erbas et al., 2021). This index was mathematically derived from the average consistency variant of ICC, a between-person measure of emotion differentiation commonly used in prior research to assess emotion differentiation. This index has no lower bound and an upper bound of 0 and it shows good predictive validity (Erbas et al., 2021). The momentary emotion differentiation index measures how consistently intensities of emotions are deviating in the same direction (i.e., positively or negatively) with regard to a person’s mean. For example, if an adolescent has a mean rating of 3 in each of the four emotions assessed 70 times, a moment when all four emotions are rated at 5 will give a low value of momentary emotion differentiation, whereas a moment when two of the four emotions are rated higher (e.g., 6 and 5) and two lower (e.g., 0 and 1) will give a high value of momentary emotion differentiation (Fig. 1).

##### Emotion Regulation Variability

We calculated momentary emotion regulation variability as Bray-Curtis dissimilarity from the emotion regulation strategy items. This index has recently been validated (Lo et al., 2024). This momentary index can be partitioned into two subcomponents that respectively detect two qualitatively different and theoretically relevant subcomponents (Aldao et al., 2015): endorsement change (e.g., from not using any strategies to using distraction) and strategy switching (e.g., replacing distraction with reappraisal). Bray-Curtis dissimilarity was calculated by comparing the moment of interest with all other moments the same adolescent reported (Fig. 2) using the *betapart* package (Baselga et al., 2022; see Github tutorial at Lo, 2023). In this way, Bray-Curtis dissimilarity reflects the within-person deviation from their typical emotion regulation style—in terms of intensity or strategy selection.^2^ Before calculating Bray-Curtis dissimilarity, we linearly transformed all emotion regulation intensity ratings by adding a small constant of 0.001 to prevent division-by-zero computational errors, so that two moments with all strategies rated 0 can still be compared. Bray-Curtis dissimilarity index falls between 0 and 1. To improve comparison with other indices, we multiplied the Bray-Curtis dissimilarity index with 10 so it ranges from 0 to 10, where 0 indicates no variability and 10 represents the maximum variability possible, based on the emotion regulation intensity it is derived from.

### Analysis

We conducted all analyses in this paper in R (R Core Team, 2023). After preparing each dataset, data were pooled into an overall dataset for analysis. To distinguish temporal effects (Hypothesis 1, 2, and exploratory research questions) from individual differences (Hypothesis 3), we separated observations of indices (emotion intensity, emotion differentiation, emotion regulation intensity, emotion regulation variability) into two components. The within-person component, which can vary at each time point, is the raw score minus the person-mean. The within-person component, which indicates an adolescent’s time-invariant difference from others, is the person-mean minus the grand-mean (Bolger & Laurenceau, 2013).

#### Main Analyses

##### Pre-Registered Hypotheses

To test our hypotheses, we ran multilevel models. In model 1A, which corresponded to Hypothesis 1, emotion differentiation was the predictor, and emotion regulation variability was the outcome. In model 2A, which corresponded with Hypothesis 2, emotion regulation variability was the predictor and emotion differentiation was the outcome. In the two multilevel models, observations (Level 1) were nested within participants (Level 2). Participants (Level 2) were further nested within datasets (Level 3) to account for between-dataset differences (see Boedhoe et al., 2019 for related methodological discussion). The outcome variables at each moment were predicted by the within-person components at Level 1 and between-person components at Level 2. We added negative emotion intensity and momentary emotion regulation intensity as covariates because we wanted to examine the relations between predictor and outcome variables above and beyond mean intensities (Dejonckheere et al., 2019; O’Toole et al., 2021). We added time as a covariate, centered with the 35.5th observation as zero (midpoint of 70 observations), to control for any systematic time trends in the data. Age and gender were also added as time-invariant covariates. Time-varying within-person components of the predictor and control variables were entered both as fixed and random effects. Random intercepts and slopes were allowed to covary. Within-person components and centered time were entered as fixed effects. We included a first-order autocorrelation structure on the residuals. We used the *nlme* package (Pinheiro et al., 2022) to estimate multilevel models with the quasi-Newton optimizer.

In Hypotheses 1 (emotion differentiation predicting subsequent emotion regulation variability) and 2 (emotion regulation variability not predicting subsequent emotion differentiation) we were primarily interested in the fixed effects of the within-person components of the predictor variables in models 1A and 2A. For Hypothesis 1, we examined if the fixed effect differed significantly from zero. For Hypothesis 2, we used the two one-sided test approach to equivalence testing (Lakens et al., 2020) by inspecting whether the 90% confidence interval of the fixed effect crossed $-$.187 and .187, the reference fixed slope we obtained in our power analysis (Supplemental Materials 1). To test Hypothesis 3 (adolescents with high emotion differentiation show high emotion regulation variability), we examined the significance of the fixed effect of between-person components in model 2A.^3^

##### Exploratory Research Questions

We ran within-person mediation models to investigate the impact from emotion differentiation (predictor) to subsequent emotion intensity (outcome) via emotion regulation variability (mediator) with the R packages *nlme* and *lme4* (Bates et al., 2015). We restructured the data by stacking, which refers to splitting each row of data into two rows where one emphasizes the outcome (emotion intensity) and the other the mediator (emotion regulation variability) (Bauer et al., 2006; Bolger & Laurenceau, 2013). By doing so, the mediation model, inherently multivariate, can be fitted in the R packages we used, which only supported univariate modeling (McNeish & MacKinnon, 2022). After restructuring the data, we estimated the within-person mediation model, model 1 M, which can be understood as an extension of Model 1A. In model 1 M, the predictor-outcome (“c’-path” from lagged differentiation to intensity) and the mediator-outcome (“b-path” from regulation variability to intensity) temporal relations were estimated simultaneously with the predictor-mediator temporal relation (“a-path” from lagged differentiation to regulation variability, originally included in Model 1A). The mediation effect is given by the sum of two components: the product of the predictor-mediator and mediator-outcome temporal relations (“a-path” and “b-path”), and the covariance of the two paths. The covariance term was included to account for how much the two paths co-vary within the same adolescents, informing the extent to which the mediation operates at the within-person level (Bolger & Laurenceau, 2013). To estimate the confidence interval of the mediation effect, we used the Monte Carlo method (Preacher & Selig, 2010), which required us to extract the following estimates of the predictor-mediator and mediator-outcome relations in model 1 M: Fixed effect, residual variance, covariance of fixed effect, covariance of random effect, and asymptotic covariance of random effects. Other details regarding the specification of model 1 M and testing the within-person mediation can be found in Supplemental Materials 5.

To test for pro-/contra-hedonic changes of emotion intensity for exploratory research question 1, we examined if the relevant fixed effects in the within-person mediation model differed significantly from zero. To test for the mediation effect for exploratory research question 2, we inspected whether the 95% confidence interval of the mediation effect contained zero.

**Fig. 3:**
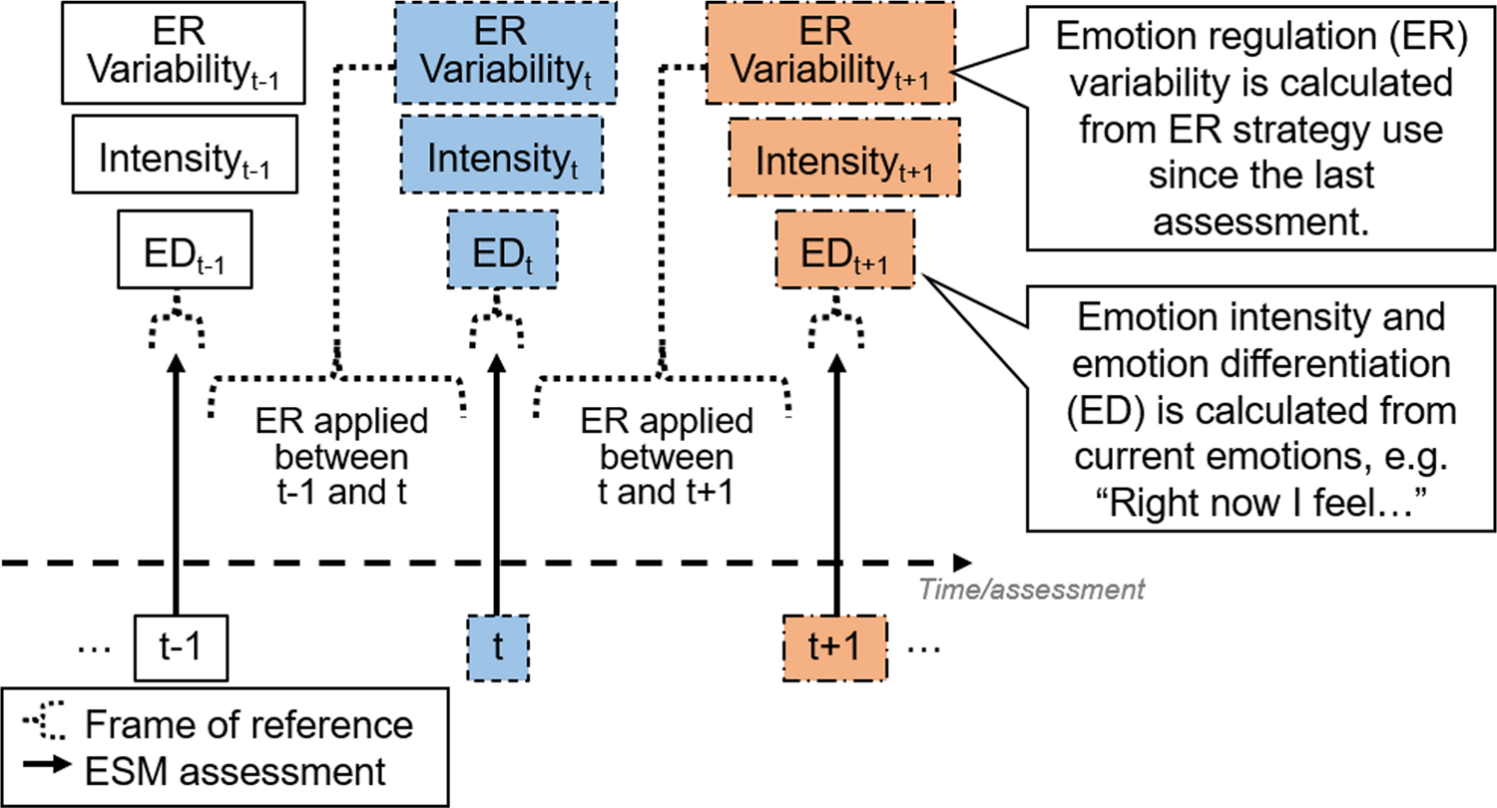
t refers to the moment of interest. Tiles with similar colors and borders belong to the same moment

#### Sensitivity Analyses

##### Different Specifications of Momentary Indices

We ran models 1B, 1C, and 2B to explore the two subcomponents of emotion regulation variability. Model 1B and 1C followed the structure of model 1A, treating emotion differentiation as the predictor, but differed as follows: Model 1B made strategy switching the outcome and added endorsement change as a covariate; model 1C made endorsement change the outcome and added strategy switching as a covariate. Model 2B followed the structure of model 2A, treating emotion differentiation as the outcome but used both emotion regulation variability subcomponents (strategy switching and endorsement change) as simultaneous predictors in replacement of the full index in model 2A. We repeated all the analyses (model 1A, 1B, 1C, 1 M, 2A, and 2B) by substituting negative emotion indices (differentiation and intensity) with positive emotion indices.

##### Robustness Across Adolescents’ Age and Upon Measurement Occasions With Zero Negative Emotion (Regulation) Intensity

We also conducted a series of sensitivity analyses to investigate the robustness of the results of all models. These analyses included using an alternative temporal comparison operationalization of Bray-Curtis dissimilarity (Supplemental Materials 6), adding within-person moderators that tested the potential influence of within-dataset age differences (Supplemental Materials 7), and adding within-person moderators that tested the potential influence of zero negative emotion (regulation) intensity (Supplemental Materials 7). In the analyses with additional moderators, we considered our results robust if the main effects (i.e., the portion of effect without age or zero intensity as moderators) of the independent variables remain similar to the results from our main analyses.

#### Frame of Reference

In all datasets, the frame of reference for rating emotion regulation strategies was about regulating the negative emotions between the previous and current assessment (e.g., “Since the last beep, to change my negative feelings, I have sought for distraction”), whereas emotion items were assessed in terms of “right now” during each assessment (Fig. 3). Therefore, associations between momentary emotion regulation variability and the emotion differentiation index, as derived from the same assessments, indicate that emotion regulation variability precedes emotion differentiation. As such, to examine Hypothesis 1 (heightened emotion differentiation is followed by subsequent increases in emotion regulation variability; model 1A to 1C), we used the lagged momentary emotion differentiation index as the predictor (and lagged momentary negative emotion intensity as covariate), and momentary emotion regulation variability as the outcome. In contrast, to examine Hypothesis 2 (emotion regulation variability does not affect subsequent emotion differentiation; model 2A and 2B), momentary emotion regulation variability as the predictor and the momentary emotion differentiation index as the outcome both came from the same assessment. Given the temporal sequence of lagged emotion differentiation, regulation variability, and emotion intensity observed in this frame of reference, we extended Model 1A to develop and run the within-person mediation model (Model 1 M).

## Results

### Descriptive Statistics

On average, adolescents showed relatively low intensity of negative emotions and emotion regulation but moderate positive emotion intensity (Table 2). With regards to emotion differentiation and emotion regulation variability indices, within-person and between-person variance indicated that there is sufficient variation across time and between people. In Supplemental Materials 3, we detailed how we inspected the indices’ distributions, assessed potential floor and ceiling effects, and compared correlations of momentary indices against published studies. In general, we considered it appropriate to further analyze emotion intensity, emotion differentiation, and emotion regulation variability indices as the primary (in)dependent variables in our hypotheses.

**Table 2:** Descriptive statistics of momentary indices of the pooled dataset (*N* = 778)

Momentary index	Minimum possible value	Maximum possible value	Mean	Between-person *SD*	Within-person *SD*	Within-person minimum	Within-person maximum
Positive emotion intensity	0.00	10.00	5.78	1.65	1.53	2.16	8.54
Positive emotion differentiation	-Infinity	0.00	−1.98	0.76	3.06	−15.25	−0.03
Negative emotion intensity	0.00	10.00	1.46	1.16	0.98	0.30	4.57
Negative emotion differentiation	-Infinity	0.00	−2.15	0.82	4.80	−28.26	−0.03
Emotion regulation intensity	0.00	10.00	2.28	1.62	1.06	0.78	5.08
Emotion regulation variability (full index)	0.00	10.00	4.03	1.78	1.13	3.04	7.29
Endorsement change subcomponent	0.00	10.00	2.35	1.47	1.13	1.50	6.12
Strategy switching subcomponent	0.00	10.00	1.68	1.05	0.75	0.38	3.65

### Main Analyses

#### Pre-Registered Hypotheses

**Fig. 4:**
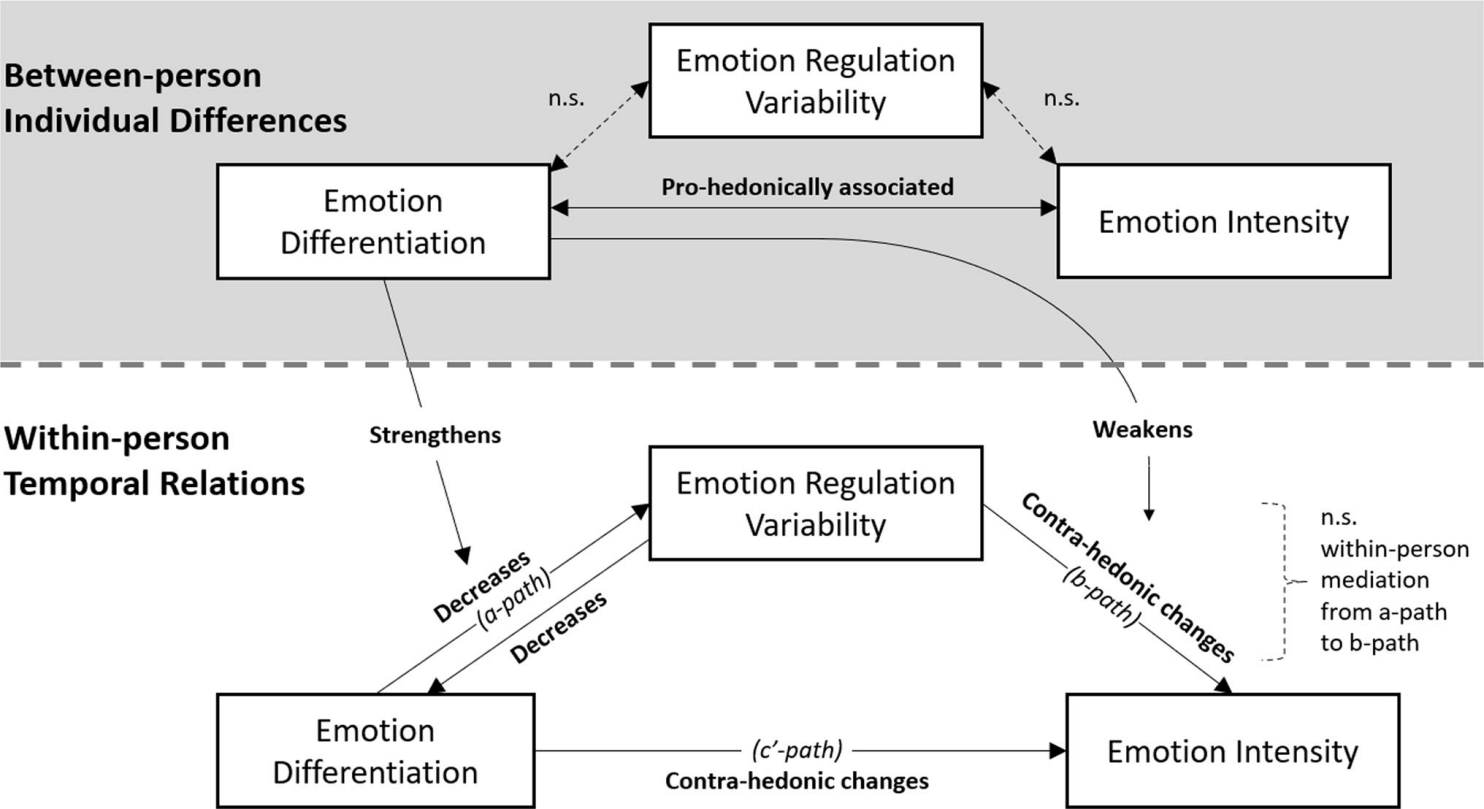
Summary of between-person individual differences and within-person temporal relations between emotion differentiation, emotion regulation variability, and emotion intensity. Pro-(contra-)hedonic refers to increased (decreased) positive emotion and decreased (increased) negative emotion intensity. n.s.: non-significant

In contrast with Hypothesis 1 (heightened emotion differentiation is followed by subsequent increases in emotion regulation variability within adolescents), model 1A (Table 3, Fig. 4) results showed negative within-person associations between negative emotion differentiation and subsequent emotion regulation variability. This indicated that higher negative emotion differentiation at one moment was related to lower emotion regulation variability within adolescents at the subsequent moment. In contrast with Hypothesis 2 (emotion regulation variability does not affect subsequent emotion differentiation within adolescents), model 2A results indicated that higher emotion regulation variability at one moment was significantly associated with decreases in negative emotion differentiation at the subsequent moment.^4^ In contrast with Hypothesis 3 (adolescents with high emotion differentiation show high emotion regulation variability), confirmatory analyses revealed no between-person association between negative emotion differentiation and emotion regulation variability (Model 2A, Table 3). This suggested that adolescents’ average levels of negative emotion differentiation and regulation variability were unrelated. However, higher baseline negative emotion differentiation was pro-hedonically associated with lower average negative emotion intensity.

#### Exploratory Research Questions

Research question 1 explores changes in negative emotion intensity subsequent to fluctuations in negative emotion differentiation and emotion regulation variability. Model 1 M suggested that both negative emotion differentiation and emotion regulation variability predicted an increase in subsequent negative emotions, bringing contra-hedonic changes. Research question 2 explores the within-person mediation effect from negative emotion differentiation to emotion intensity via emotion regulation variability. The temporal relations between the predictor and mediator (“a-path” from lagged differentiation to regulation variability) and the mediator and outcome (“b-path” from regulation variability to intensity) were both significant on average across all adolescents. However, there was no evidence on mediation effect, as the 95% confidence intervals for the indirect effect included zero. This suggests that the two temporal paths covaried in a manner that offset the potential mediation effect. Specifically, adolescents with a stronger a-path tended to have a weaker b-path, and vice versa, resulting in no overall within-person mediation. This covariance between the a-path and b-path can be characterized as co-moderation, meaning that both paths were simultaneously moderated. A further exploratory analysis showed that between-person negative emotion differentiation could be such a co-moderator: Higher baseline negative emotion differentiation intensified the negative a-path (moderated *b* = $-$0.005 [$-$0.009, $-$0.002]) and weakened the positive b-path (moderated *b* = $-$0.034 [$-$0.057, $-$0.011]) in Model 1 M.

**Table 3:** Fixed effect estimates in within-person temporal associations and between-person differences between emotion differentiation and emotion regulation variability

	Negative emotions *b *[95% *CI]*	Positive emotions *b *[95% *CI]*	Model
Within-person temporal hypotheses			
H1: Higher emotion differentiation is associated with subsequently higher emotion regulation variability (*N* = 751, *n* = 25,851)			
Emotion differentiation $\rightarrow$ Emotion regulation variability	$-$**.009** [$-$**.014**, $-$**.005**]	$-$**.009** [$-$**.014**, $-$**.004**]	1A
Emotion differentiation $\rightarrow$ Strategy switching	$-$**.004** [$-$**.007**, $-$**.002**]	$-$**.004** [$-$**.007**, $-$**.000**]	1B
Emotion differentiation $\rightarrow$ Endorsement change	$-$**.008** [$-$**.012**, $-$**.004**]	$-$**.007** [$-$**.012**, $-$**.003**]	1C
H2: Emotion regulation variability is not associated with subsequent changes in emotion differentiation (*N* = 750, *n* = 25,830)			
Emotion regulation variability $\rightarrow$ Emotion differentiation	$-$**.514** [$-$**.731**, $-$**.296**]	$-$**.276** [$-$**.496**, $-$**.057**]	2A
Strategy switching $\rightarrow$ Emotion differentiation	$-$**.432** [$-$**.730**, $-$**.133**]	$-$**.306** [$-$**.525**, $-$**.086**]	2B
Endorsement change $\rightarrow$ Emotion differentiation	$-$**.550** [$-$**.771**, $-$**.328]**	$-$**.262** [$-$**.480**, $-$**.043**]	2C
RQ: Emotion differentiation affects subsequent emotion intensity via emotion regulation variability (*N* = 755, *n* = 51,991)			
a-path: Emotion differentiation $\rightarrow$ Emotion regulation variability	$-$**.013** [$-$**.018**, $-$**.008**]	$-$**.014** [$-$**.020**, $-$**.008**]	1 M
b-path: Emotion regulation variability $\rightarrow$ Emotion intensity	**.073 [.038, .108]**	$-$**.049** [$-$**.091**, $-$**.006**]	1 M
c’-path: Emotion differentiation $\rightarrow$ Emotion intensity	**.008 [.003, .013]**	$-$**.016** [$-$**.026**, $-$**.006**]	1 M
Mediation (sum of covariance and product of a- and b-path)	$-$.000 [$-$.001, .001]	$-$.000 [$-$.001, .001]	1 M
Between-person hypothesis			
H3: Higher emotion differentiation is associated with higher emotion regulation variability (*N* = 750)			
Emotion differentiation $\leftarrow$ $\rightarrow$ Emotion regulation variability	$-$.035 [$-$.072, .001]	$-$.012 [$-$.039, .015]	2A
Emotion differentiation $\leftarrow$ $\rightarrow$ Strategy switching	.055 [$-$.008, .118]	$-$.004 [$-$.052, .044]	2B
Emotion differentiation $\leftarrow$ $\rightarrow$ Endorsement change	$-$**.091** [$-$**.140**, $-$**.042**]	$-$.018 [$-$.055, .019]	2B
Other exploratory analyses (*N* = 750)			
Emotion intensity $\leftarrow$ $\rightarrow$ Emotion differentiation	$-$**.238** [$-$**.296**, $-$**.180**]	**.035 [.005, .065]**	2A
Emotion intensity $\leftarrow$ $\rightarrow$ Emotion regulation variability	$-$.023 [$-$.128, .083]	$-$**.107** [$-$**.181**, $-$**.034**]	1A
Emotion intensity $\leftarrow$ $\rightarrow$ Strategy switching	.032 [$-$.022, .085]	$-$.035 [$-$.073, .002]	1B
Emotion intensity $\leftarrow$ $\rightarrow$ Endorsement change	$-$.072 [$-$.148, .004]	.025 [$-$.028, .079]	1C

*Note: * Significant effects are displayed in bold. $\rightarrow$: temporal precedence; $\leftarrow$ $\rightarrow$: between-person association; n: number of ESM assessments with complete observations of all indices required for modeling; N: number of adolescents; b: unstandardized effect; CI: confidence interval; H1 - H3: Hypotheses 1 to 3. RQ: Exploratory research questions. Negative emotions and positive emotions were analyzed in separate models. Small differences in n and N between models exist due to different availability of indices as required in the different models. For brevity, we displayed the smaller n and N of the models grouped under the same hypotheses. H1 was tested using three negative emotion models and three positive emotion models because of three outcome variables (emotion regulation variability and its two subcomponents). H2 was tested using two models for positive emotions and two models for negative emotions. Two subcomponents were included together in model 2B. In Model 1 M, n is doubled because of how data have undergone the stacking preparation step. Full model results with estimates of covariates (emotion intensity, emotion regulation intensity, time, gender, and age) are available in Supplemental Materials 5

### Sensitivity Analyses

All three hypotheses and exploratory research questions were generally robust against sensitivity analyses: They held for both positive and negative emotion intensity and differentiation, for both subcomponents of emotion regulation variability (Table 3 and Supplemental Materials 5), alternative specification of Bray-Curtis dissimilarity (Supplemental Materials 6), or when moderation effects of age and zero emotion (regulation) intensity on the hypothesized within-person relations were controlled for (Supplemental Materials 7 and 8). In other words, emotion differentiation—whether positive or negative—and emotion regulation variability, regardless of the specific subcomponent, seemed to hinder each other subsequently. Additionally, emotion differentiation and emotion regulation variability both introduce subsequent contra-hedonic changes, in terms of increased negative emotion and decreased positive emotion intensity. However, in terms of individual differences, adolescents with higher emotion differentiation tended to have more pro-hedonic emotion intensity in general (higher positive emotion and lower negative emotion intensity). Evidence of robustness was the strongest for our pre-registered hypotheses specified with negative emotions. Exploratory research questions results were also generally robust, but with increasingly nuanced evidence for analyses with compounding exploratory specifications (e.g., positive emotions and moderation by age).

Across datasets, the within-person effects in our main analyses were consistent in direction, indicating that the results were driven collectively by all datasets rather than being influenced disproportionately by one or two (Supplemental Materials 8). Our within-person results appeared to be stronger among datasets that sampled late adolescents. However, in most models, dataset-centered age did not moderate the within-person relations.

## Discussion

Using five ESM datasets that encompassed 25,834 observations in 750 adolescents, we tested whether higher emotion differentiation was related to higher subsequent emotion regulation variability and changes in emotion intensity. Contrary to expectations, our pre-registered analyses showed that momentarily heightened differentiation of negative or positive emotions predicted lower subsequent emotion regulation variability, indicating greater stability in deploying regulation strategies. Reciprocally, increased deviation from typical emotion regulation strategies (i.e., higher variability) predicted less emotion differentiation at the next assessment. Exploratory analyses further showed that moments of heightened emotion differentiation and regulation variability were both followed by feeling worse, with increased negative and decreased positive emotion intensity. These effects were consistent across two subcomponents of regulation variability (endorsement change and strategy switching) and held true regardless of whether emotion differentiation involved positive or negative emotions.

Although our results did not reveal between-person associations between emotion differentiation and emotion regulation variability, individual differences in emotion differentiation might have moderated within-person processes. Specifically, the higher baseline negative emotion differentiation adolescents have, the more intensified negative reciprocal relations between negative emotion differentiation and emotion regulation variability are, but the more adolescents are buffered from contra-hedonic changes in negative emotion that follow momentarily higher emotion regulation variability.

In summary, our results add to the theoretical understanding of how emotion differentiation may influence emotion regulation. At both within-person and between-person levels, emotion differentiation influences subsequent within-person fluctuations in emotion regulation strategy use and emotion intensity.

### Possible Explanations of the Interplay between Emotion Differentiation, Emotion Regulation Variability, and Emotion Intensity

A possible explanation for the negative reciprocal relationship between emotion differentiation and emotion regulation variability is that these processes may compete for the same mental resources; when one is more active, the other may consequently decline. Mental effort could represent such a cost. Emotion differentiation has been theorized as an effortful process in daily life (Erbas et al., 2019; Wranik et al., 2007) and shown to be so in experimental settings requiring participants to label emotions (Lieberman et al., 2011; Torre & Lieberman, 2018). Additionally, a recent review indicated that emotion regulation demands effort and can lead to fatigue (Lewczuk et al., 2022). Given that both processes require effort, high emotion differentiation may restrict variability in emotion regulation strategies, and vice versa. This “effort as cost” perspective may also explain changes in negative emotion intensity. A recent meta-analysis synthesizing 170 studies revealed that mental effort strongly correlates with higher negative emotion intensity across various tasks and populations, including late adolescents aged 18 to 25 (David et al., 2024). Consistent with this finding, experiments have demonstrated that labeling emotions in addition to initiating a regulation strategy counteracts the strategy’s pro-hedonic effects in responding to aversive stimuli (Nook, Satpute et al., 2021). Thus, our findings that negative emotion intensity increased following emotion differentiation and emotion regulation variability may result from—or reflect—the exertion of mental effort.

Contra-hedonic changes in emotion intensity may also be explained by the assumption that there is an increase in attention to emotion following increased emotion differentiation (Thompson et al., 2011). However, this intensifying mechanism explains only the increase in negative emotion intensity, not the decrease in positive emotion intensity. One possible explanation lies in the differing tendency of attending to positive versus negative emotions. Individuals, including late adolescents aged 18 to 25, typically avoid negative emotions and embrace positive ones; studies indicate a tendency to approach positive-valence stimuli and avoid negative ones (Krieglmeyer et al., 2010; Phaf et al., 2014) by resisting attention to aversive experiences (Lee et al., 2024). Hence, it is possible that to heighten emotion differentiation, individuals must pay extra attention to negative emotions, but not necessarily so to positive emotions, because they already do. This could lead to a “double increase” in negative emotion intensity, both due to the greater attention and exertion of effort. In contrast, positive emotion intensity may decrease because the effortful nature of differentiation likely outweighs the minimal intensifying effects on positive emotions due to little attentional increase.

### Baseline Negative Emotion Differentiation May Co-moderate the Two-Step Within-Person Processes

Our within-person results on the temporal sequence—from emotion differentiation to regulation variability, and from regulation variability to intensity change—appear to suggest that differentiated emotions help adolescents’ emotion regulation by fostering consistent use of strategies (resulting in low variability) that lead to pro-hedonic emotion intensity outcomes. However, the within-person mediation analyses do not support this two-step pathway. Instead, our results highlight individual differences in these sequential processes by how they have been co-moderated. This co-moderation effect reveals that adolescents who display a stronger connection in one of these relations tend to show a weaker connection in the other. Our results suggest that baseline emotion differentiation at the person level may act as a co-moderator. Specifically, high baseline negative emotion differentiation intensifies the negative temporal relation from negative emotion differentiation to emotion regulation variability, while buffering adolescents from contra-hedonic outcomes following increased regulation variability. As a result, within-person changes in emotion intensity arise directly from differentiation itself, rather than being mediated through regulation variability.

### Are Momentary Contra-Hedonic Emotion Intensity Changes at Odds With the Long Term Benefits of Emotion Differentiation?

Our results show that adolescents with higher baseline emotion differentiation are more likely to have higher levels of positive emotions and lower levels of negative emotions in general (Table 3, Supplemental Materials 3). These are in line with earlier reports describing that individuals with higher baseline emotion differentiation tend to experience better well-being (Seah & Coifman, 2024). Cross-sectional data have suggested that adolescents may experience a dip in their emotion differentiation before developing to higher levels as they age (Nook et al., 2018). A promising direction for future research would be to examine whether repeated momentary efforts to increase emotion differentiation yield long-term benefits in improving baseline emotion differentiation and well-being. While short-term contra-hedonic outcomes may seem like an obstacle for voluntarily heightening momentary emotion differentiation, adolescents may be well-suited for this challenge: Compared to older adults, adolescents are more inclined to tolerate contra-hedonic experiences if such experiences contribute to long-term goals (Riediger et al., 2009; Tamir, 2009).

### Open Developmental and Contextual Questions in Emotion Differentiation and Emotion Regulation Variability

Due to differing study designs across datasets and lacking contextual data, it was not feasible for us to formally test age differences or contextual influence.

Future research should explore the development of emotion differentiation and emotion regulation variability across adolescence, ideally using a single large dataset encompassing the entire adolescent age range. Drawing on prior work that suggested nonlinear development in emotion differentiation (Nook et al., 2018), researchers may investigate whether emotion regulation variability also follows a nonlinear trajectory during adolescence. This could be due to adolescents’ intermediate experimentation with an expanding repertoire of regulation strategies (Elkjær et al., 2022). Middle adolescence, in particular, may feature heightened variability, as adolescents in this stage are less likely to regulate emotions like sadness and anger compared to younger or older peers (Zimmermann & Iwanski, 2014). These middle adolescents may have more frequent all-or-nothing changes in employing emotion regulation strategies, leading to greater observed variability.

Future research should investigate how emotion regulation variability relates with contexts. Our exploratory findings indicated that increased emotion regulation variability preceded contra-hedonic changes in emotion intensity, a result contrasting with Lo et al. (2024)’s initial findings, which suggested that this variability reduces subsequent negative emotion intensity (but did not control for covariates such as prior emotion regulation intensity). Additionally, the contra-hedonic effect of heightened emotion regulation variability was moderated by person-level emotion differentiation. This aligns with recent literature showing that other conditions, such as emotion regulation goals and contexts, significantly shape emotion regulation variability (Liao et al., 2024). Furthermore, it has been proposed that emotion regulation variability attuned to shifting contexts or that emerged when prior strategies are ineffective differs conceptually from variability that is context-insensitive (Kalokerinos et al., 2022; Southward et al., 2018). Interpreting emotion regulation variability in relation to changes in contexts and goals enables researchers to ask under what contexts high or low variability benefits adolescents. These questions could enhance our understanding of the dynamics between context and strategy use, which is increasingly seen as central to defining adaptive emotion regulation in daily life (Aldao et al., 2015; Paul et al., 2023).

### Limitations

Other limitations must be considered when interpreting our results. First, there is heterogeneity across datasets due to varying sample characteristics and ESM protocols. We have included dataset-level random intercepts to mitigate this, but future studies should explore how these study characteristics affect outcomes. Second, the generalizability of our conclusions depends on the scope of emotions and emotion regulation items included. Caution must be applied in generalizing sensitivity analysis results on positive emotions due to having few items in some datasets for forming positive emotion momentary indices. In contrast, our confirmatory results about negative emotion differentiation are more generalizable because of being derived from at least four negative emotion items. Although our datasets selected conventional items from emotion (regulation) theories (Supplemental Materials 2), they did not cover maladaptive behaviors such as non-suicidal self-injury (Zaki et al., 2013) and alcohol consumption (Kashdan et al., 2010), which have been linked to poorer negative emotion differentiation. These behaviors can be treated as emotion regulation strategies in an expanded framework of emotion regulation (Seah & Coifman, 2021). Therefore, future studies may reexamine our results by widening the scope of emotions and emotion regulation items. Researchers may additionally consider the use of personalized items (e.g., Olthof et al., 2023), given the idiographic nature of emotion and emotion regulation (Entwistle et al., 2023; Grommisch et al., 2020). Third, in our analysis, we assumed equal intervals in the temporal sequences of emotion differentiation and emotion regulation variability, but in reality, they varied due to study designs (Table 1) and the frame of reference (Fig. 3). Future research should consider methodologies that can model irregular time intervals (e.g., Asparouhov & Muthén, 2020) to validate our findings.

### Practical Implications

Our study provides three considerations for practitioners in emotion-focused psychoeducation (e.g., Metz et al., 2013). First, training emotion differentiation and regulation variability separately may be more effective than a combined one-session approach. Our within-person findings suggest these processes can hinder each other, and combining them may be counterproductive. Second, practitioners should anticipate short-term discomfort following increased emotion differentiation or regulation variability. To support participants, they might consider complementing training with techniques to hasten recovery from worsened feelings and emphasize the long-term benefits to maintain motivation. Third, pre-training assessments of adolescents’ baseline emotion differentiation could be valuable. Our between-person findings suggest that adolescents vary in training needs; for instance, those with higher baseline differentiation may show a stronger negative relationship from differentiation to regulation variability, while others may experience a stronger positive link from regulation variability to contra-hedonic outcomes. However, it is important to note that our findings are correlational and do not predict how these processes may interact post-intervention.

### Conclusion

To conclude, this well-powered study is the first to test how emotion differentiation temporally influences emotion regulation variability and emotion intensity in adolescents’ daily lives. Our findings suggest that, at least in the short term, emotion differentiation and emotion regulation variability hinder each other, regardless of the type of variability (endorsement change or strategy switching) or valence of emotions (positive or negative). Furthermore, contra-hedonic emotional intensity changes follow momentarily heightened emotion differentiation or regulation variability. Adolescents differ in these within-person processes. Specifically, high baseline emotion differentiation intensifies the negative reciprocal relationship between differentiation and regulation variability, while buffering them from contra-hedonic outcomes following increased regulation variability. These results prompt reconsideration of how emotion differentiation supports emotion regulation, highlighting within-person processes that may enable practitioners to better tailor emotion-focused mental health interventions for adolescents.

## Supplementary Information

Below is the link to the electronic supplementary material.

Supplementary file 1 (pdf 1035 KB)

## Footnotes

1 We followed a recent definition of adolescence as ages 10 to 25 (Sawyer et al., 2018).

2 Another method to compute Bray-Curtis dissimilarity is by contrasting each moment with the preceding one. We ran sensitivity analyses with this successive temporal comparison approach. Results were generally consistent with what we present in the main text. Details can be found in Supplemental Materials 6.

3 Model 2A was selected over model 1A for testing to allow easier comparison of estimates with our exploratory findings from model 2B. For sensitivity analyses, we selected model 2B to simultaneously assess both subcomponents on one side as predictors, unlike models 1B and 1C which split them as outcomes and predictors.

4 Technically, the fixed effect tested in Model 2A crossed the reference slope at $-$0.187, meaning the equivalence proposed in Hypothesis 2 could not be confirmed. Additionally, the 95% confidence interval of the fixed effect did not include 0, indicating non-equivalence and suggesting that heightened emotion regulation variability was significantly associated with subsequent decreases in emotion differentiation.

## Additional Information

### Availability of data and material

Following the Workflow for Open Reproducible Code in Science (Van Lissa et al., 2021), the preregistration (hypotheses and analysis plan), materials, primary data and analysis codes of this study are available via https://osf.io/cq6n4/.

### Preregistration

The hypotheses and methods were preregistered (https://osf.io/9vx7t/) on 2022-05-04 prior to accessing the five datasets used in this study. There was an update of preregistration on 2023-10-19 for the purpose of including more datasets to achieve sufficient power for our hypotheses (for details, see Supplementary Materials 1).
