# Supplementary material for: Emotion Differentiation in Adolescents: Short-term Trade-offs with Regulation Variability and Emotion Intensity
Source: Affect Sci. 2025 May 28;6(2):243–58. doi: 10.1007/s42761-025-00301-4 (PMC12209115; doi:10.1007/s42761-025-00301-4)
Supplement: Supplementary file 1 — (pdf 1035 KB) [file 42761_2025_301_MOESM1_ESM.pdf]

Supplemental Materials: Emotion Differentiation in Adolescents: Short-term Trade-offs with Regulation Variability and Emotion Intensity

Tak Tsun Lo[1], Maaike Verhagen[1], J. Loes Pouwels[1], Eeske van Roekel[2], Sarah O'Brien[3], Gillian Debra[4], Jolien Braet[4], Jacqueline M. Vink[1], and Dominique F. Maciejewski[2]

[1]Behavioural Science Institute, Radboud University

[2]School of Social and Behavioral Sciences, Tilburg University

[3]Melbourne School of Psychological Sciences, the University of Melbourne

[4]Faculty of Psychology and Educational Sciences, Ghent University

Supplemental Materials 1: Pre-registration, *a priori* Power Analysis, and Deviations

Pre-registration: the Original and Updated Version

On 04 May 2022, we submitted our original version of pre-registration [https://osf.io/9vx7t?revisionId=62723c863252440156414dd8&view_only= bbeadda0702c4a6696d906bbf8faaa83]. While we initially expected to have sufficient power to test our hypotheses usingthe G(F)ood together dataset from Radboud University, we are now using Bray-Curtis dissimilarity, a newly proposed emotion regulation variability (Lo et al., 2024), for testing our hypotheses. Therefore, we updated the power analysis. The new power analysis revealed that we are underpowered at 30% to test our hypotheses with multilevel modeling with only the G(F)ood together dataset. To ensure sufficient power, we decided to include more experience sampling method (ESM) datasets to test our hypotheses. We reached out to researchers who used ESM in Dutch-speaking regions with the same specified inclusion criteria in terms of frame of reference of ESM items and age group. We received favorable replies from researchers in accessing four ESM datasets, which provided us with a large enough sample size to reach 80% power. The pre-registered questions and hypotheses remained the same. We updated our pre-registration on 19 Oct 2023 prior to accessing the new datasets [https://osf.io/9vx7t?view_only=bbeadda0702c4a6696d906bbf8faaa83].

Updated Power Analysis

The pooled sample size across five datasets was 811. We used the PowerAnalysisIL Shiny app (Lafit et al., 2021) to calculate power for Hypothesis 1 (greater emotion differentiation at a given moment will result in heightened variability in emotion regulation at the subsequent moment) and Hypothesis 2 (variability in emotion regulation at one moment will not be associated with emotion differentiation at the following moment). We obtained parameters needed analyzing an unrelated ESM dataset collected by another

researcher in Radboud University not involved in this specific project (Mosannenzadeh, 2021).

Hypothesis 1

Power analysis results for Hypothesis 1 are shown in Table S1.1. We concluded that power is likely to be over 80% when the final sample size approaches 800.

Table S1.1

Hypothesis 1 Power Analysis Results

Power Analysis Setup		Power Analysis Result	
Parameters	Value	Number of Participants	Simulated Power
Outcome	Emotion regulation variability	100	0.186
Predictor	Emotion differentiation	300	0.46
Number of observations per participant	13	500	0.681
Fixed Intercept	3.208	700	0.796
Fixed Slope	-0.016		
SD of error residual	0.636		
Autocorrelation of level-1 errors	0.21		
SD random intercept	0.738		
SD random slope	0.027		
Correlation (random intercept and random slope)	-0.174		
Mean of predictor	3.221		
SD of predictor	1.175		
Estimate AR(1) correlated errors	Yes		
Type I error	0.05		
Monte Carlo Replicates	1000		
Method	Maximizing the log-likelihood		

Hypothesis 2

Power analysis results for Hypothesis 2 are shown in Table S1.2. For Hypothesis 2, there was already enough power by only just using the G(F)ood together dataset (N after exclusion criteria applied = 83).

Table S1.2

Hypothesis 2 Power Analysis Results

Power Analysis Setup		Power Analysis Result	
Parameters	Value	Number of Participants	Simulated Power
Outcome	Emotion differentiation	80	0.938
Predictor	Emotion regulation variability	90	0.966
Number of observations per participant	13	100	0.984
Fixed Intercept	-1.75		
Fixed Slope	-0.187		
SD of error residual	2.583		
Autocorrelation of level-1 errors	0.118		
SD random intercept	0.514		
SD random slope	0.417		
Correlation (random intercept and random slope)	0.124		
Mean of predictor	-2.883		
SD of predictor	6.079		
Estimate AR(1) correlated errors	Yes		
Type I error	0.05		
Monte Carlo Replicates	1000		
Method	Maximizing the log-likelihood		

Deviations from pre-registration

Our study had four minor deviations from its original pre-registration.

First, in section 19 and 28 (indices), we initially planned to use intraclass correlation coefficient (ICC) for between-person emotion differentiation to test the between-person Hypothesis 3 (stated as Hypothesis 1 in the original pre-registration). In our actual analyses, we did not use ICC, but the between-person component of the momentary emotion differentiation index (Erbas et al., 2021). We considered this deviation a better approach because the within-person and between-person hypotheses could be tested together. Momentary emotion differentiation index, derived from ICC, was shown to be statistically perfectly related to ICC (Erbas et al., 2021). This supports us using the momentary emotion differentiation index in substitution of ICC in testing Hypothesis 3.

Second, in section 22 (analysis plan), we initially planned to test the between-person Hypothesis 3 (originally Hypothesis 1 in the pre-registration) with hierarchical regressions. In our actual analysis, we instead tested this hypothesis by examining the fixed effect estimates of the time-invariant between-person components in multilevel models. Although a minor procedural deviation, this approach is statistically highly similar as the pre-registered approach. Just like the first deviation, we chose this because this approach allows us to test the within-person and between-person hypotheses could be tested together.

Third, in section 27 (data exclusion), we specified the exclusion of data with zero variance across all observations. However, we did not clarify if this zero variance criterion was to be applied at the item level (e.g., for a specific emotion like sadness) or at the factor level (e.g., for a group of related emotions such as sad, angry, depressed, and anxious, useful in calculating negative emotion intensity and differentiation). In our actual analysis, we opted for the factor-level application. This decision was based on the understanding that some items might not be relevant to participants (see Discussion), leading to zero ratings, but this would not necessarily indicate poor data quality if there was variance in

other items within the same factor. Additionally, our dynamic indices evaluate multiple items, not just single ones. Applying the exclusion criterion at the factor level aligns more closely with our research objectives and ensures a more accurate assessment of data quality than excluding data based on single-item zero variance.

Fourth, in Section 28, we initially planned an exploratory analysis on the differentiation of positive emotions. Beyond this planned analysis, we conducted additional exploratory analyses: (a) within-person mediation on the temporal sequence from emotion differentiation to emotion regulation variability to emotion intensity, (b) an alternative specification of Bray-Curtis dissimilarity using the successive difference temporal comparison approach (Supplemental Materials 6), (c) the moderating effects of within-dataset age differences on our main hypotheses, and (d) the moderating effects of zero negative emotion (regulation) intensity on our main hypotheses.

Supplemental Materials 2: Participants, Procedures and ESM Measures per Dataset

Note that though descriptions of ESM measures are in English here, questionnaires were presented in Dutch to participants across the five studies.

We assessed the validity of ESM measures in four steps that were recognized as good practices given the current state of development in ESM measures validation (Vogelsmeier et al., 2023). First, we documented the reliability of measures in our samples (ESM Measures subsection in each dataset in Supplemental Materials 2). Second, we cited how these measures have been validated or used in earlier studies (Supplemental Materials 2). Third, we inspected the distributions of measures in our samples and compared them with those reported in earlier studies (Supplemental Materials 3). Fourth, we compared relations between measures in our samples against those reported in earlier studies (Supplemental Materials 3).

Most studies that we cited for the purpose of assessing ESM measures validity had samples with mean ages that fell between early to late adolescence (Sawyer et al., 2018): (In ascending order of age) Schneiders et al. (2006); Achterhof et al. (2022); Bülow et al. (2022); Rauschenberg et al. (2017); Hasmi et al. (2017); Bennik (2015); Barrantes-Vidal et al. (2013); Fried et al. (2022); Medland et al. (2020); Bakker et al. (2019); Brans et al. (2013). We also included studies with a wider age range but still covered adolescent participants (Barge-Schaapveld et al., 1999; Bastiaansen et al., 2018; Delespaul & DeVries, 1987; Jacobs et al., 2007; Kiekens et al., 2023) and a few that covered only adults (Hartley et al., 2014; Myin-Germeys et al., 2000; Spence et al., 2014; van Eck et al., 1998). In the subsequent pages, where we detail each dataset we analyzed, the mean age and standard deviation of each sample are specified under the "Participants" heading (page 8, 11, 13, 14, and 17, corresponding to the five datasets).

Dataset 1: G(F)ood together, Radboud University (main reference: Verhagen et al., 2022)

Participants

This study was part of a larger project (G(F)ood together, in Dutch: G(V)oed voor elkaar; see van den Broek et al. (2020) for other details) that studied adolescents' eating behaviours and health with six longitudinal waves of data collection across 2017 to 2021 and one ESM study (in 2021) among Dutch adolescents and their parents. The study procedures were approved by the Ethics Committee Social Sciences of Radboud University, Nijmegen, the Netherlands (ECSW20170805-516). The ESM study was administered between the fifth and sixth wave in June and July 2021. An active parental consent procedure was used for the participation of the ESM study.

The goal for the ESM study was to recruit a subsample of 100 participants. 257 families whose parents or adolescents remained active at wave 5 of the G(F)ood together study were invited to participate in the ESM study, resulting in the inclusion of 89 adolescent participants (age M = 16.42, SD = 0.61) and one of their parents. After excluding observations in which each ESM item was completed in less than 500ms (potential careless responding) and excluding participants who showed zero variance across all ESM items, the final sample size consisted of 83 participants (age M = 16.43, SD = 0.68, female = 57.63%). Most of the participants were born in the Netherlands (97.59%).

Procedure

All participants completed the ESM using the SEMA-app (version 3, O'Brien et al., 2023) which they installed on their mobile phones a few days before starting the study. A semi-random sampling scheme was employed, with participants receiving 10 notifications per day at random moments within a fixed time interval spanning from 07.30 a.m. to 09.00 p.m. over seven consecutive days. Upon receiving a notification, participants had a 30-minute window to complete the ESM assessment. For the end-of-the-day assessment, a

longer period of 149 minutes was allowed. In cases where participants did not open the momentary assessments, the app sent two reminders at 15 minutes and 25 minutes after the initial notification (75 minutes and 145 minutes for the end-of-the-day assessment). Participants responded to 3674 out of 6020 (61%) ESM notifications sent. The median number of assessments completed per participant was 47 out of 70 (67%; M = 41.83, SD = 17.06). All participants entered into a raffle for two €250 vouchers. Participants were paid at least €5 and up to €25 if they and their parents both had high compliance in the study.

ESM Measures

Emotions. At each momentary assessment, participants rated four positive emotions (content, relaxed, joyful, and energetic) and five negative emotions (irritated, worried, depressed, insecure, and lonely) presented in a randomized order on a 10-point slider scale (0 = not at all, 10 = a lot). The stem for these items was "Right now I feel [emotion]." These items have been used in other ESM studies (Achterhof et al., 2022; Bakker et al., 2019; Barge-Schaapveld et al., 1999; Barrantes-Vidal et al., 2013; Bastiaansen et al., 2018; Bennik, 2015; Bülow et al., 2022; Delespaul & DeVries, 1987; Fried et al., 2022; Hasmi et al., 2017; Jacobs et al., 2007; Kiekens et al., 2023; Myin-Germeys et al., 2000; Rauschenberg et al., 2017; Schneiders et al., 2006; van Eck et al., 1998). With 10 daily assessments over 7 days, the maximum possible number of measurements for negative and positive emotions was 70. Reliability was satisfactory for positive emotions (.70) and negative emotions (.66).

Emotion regulation strategies. At each even beep throughout the day (i.e., assessed five times daily), following the rating of negative emotions, participants responded to one additional question on a slider scale regarding the intensity of the most unpleasant event since the previous beep ("Think about the most unpleasant thing that you have experienced, since the last beep. How unpleasant was it?" 0= not at all unpleasant, 10 = very much unpleasant). If the unpleasantness was 5 or higher, participants had the

opportunity to rate their use of emotion regulation strategies related to the event. This branching was introduced with a rationale of collecting reports with more intensive use of emotion regulation strategies. At the final beep of each day, regardless of event intensity, questions about emotion regulation strategies were asked. Adapted from Brans et al. (2013), for each of the five emotion regulation strategies listed below, participants rated their use on a 11-point scale (0 = not applicable at all, 10 = very applicable): acceptance ("I have accepted my feelings about it"), reappraisal ("to feel better, I have changed the way I think about it"), expression suppression ("I have avoided expressing my feelings about it"), rumination ("I couldn't stop thinking my feelings about it"), and sharing ("I talked about it to someone"). These strategies have been assessed in previous ESM studies (Hartley et al., 2014; Kiekens et al., 2023, 2023). With 5 even-beep assessments over 7 days, the maximum possible number of measurements for emotion regulation strategies was 35. Adolescents had a total of 719 beeps which they had the opportunity to report emotion regulation strategy use from 575 end-of-day beeps and 144 non-end-of-day even beeps which they rated having experienced a negative event with unpleasantness at 5 or above. Adolescents reported their use of emotion regulation strategies in 586 out of the 719 possible beeps (81.50%). Reliability was satisfactory for emotion regulation strategies (.59).

Dataset 2: Emotions in daily life 2011, KU Leuven (main reference: Koval et al., 2013)

Participants

Participants were recruited from a pool of 439 undergraduates at the University of Leuven, Belgium, in a study which the ethics committee of the University of Leuven approved of. All undergraduates completed a Dutch translation of the Center for Epidemiologic Studies Depression Scale (CES-D, Radloff, 1977) and were further selected to maximize variation in depression scores. The target sample of 100 participants were contacted in 2011. Three participants were excluded because the devices they used had

malfunction. There was no further exclusion based on careless responding (<500 ms) or zero variance instances. The final sample consisted of 97 participants. Mean age of the sample was 19.05 years (*SD* = 1.27), and 63% were women. Majority of the sample had Belgian nationality (97%).

Procedure

Participants took part in an introductory session in the laboratory, in which they gave informed consent to participate, filled out questionnaires unrelated to the current study, and received standardized devices (Tungsten E2 PalmOne, Mankato, MN), which were programmed to assess ESM items. The ESM study started the following day and lasted 7 days, during which 10 beeps occurred semi-randomly each day in a 12-hr time frame. Participants were informed that completing one measurement would take an average of 1 minute. Participants had to start the questionnaire within 2 minutes after the notification. Participants had 90 seconds to answer each question once they opened the questionnaire before it timed out. There were no reminders for participants in case they did not open the momentary assessments Participants answered 91.5% of the beeps (*SD* = 6.2%, range: 67–100% of all beeps). The participants were reimbursed with 70 Euros for the entire study.

ESM measures

Emotions. At each momentary assessment, participants rated two positive emotions (relaxed, happy) and four negative emotions (angry, sad, anxious, and depressed) presented on a 100-point slider scale (1 = not at all, 100 = very much). The stem for these items was "How [emotion] do you feel at the moment?" These items have been used in other ESM studies (Achterhof et al., 2022; Bakker et al., 2019; Barge-Schaapveld et al., 1999; Bastiaansen et al., 2018; Bennik, 2015; Brans et al., 2013; Bülow et al., 2022; Delespaul & DeVries, 1987; Fried et al., 2022; Hasmi et al., 2017; Jacobs et al., 2007; Kiekens et al., 2023; Myin-Germeys et al., 2000; Rauschenberg et al., 2017; Schneiders et

al., 2006). With 10 daily assessments over 7 days, the maximum possible number of measurements for negative and positive emotions was 70. Reliability was satisfactory for positive emotions (.71) and negative emotions (.76).

Emotion regulation strategies. At each momentary assessment, participants rated the extent they used six emotion regulation strategies presented on a 100-point slider scale (1 = not at all, 100 = very much so). The stem for these items was “Since the last beep, did you…” and ended with “ruminate about your feelings” (rumination), “calmly reflect on your feelings?” (reflection), “see the event that caused your feelings from a different perspective?” (reappraisal), “try to distract yourself from your feelings?” (distraction), “suppress the expression of your feelings?” (expressive suppression), and “talk with others about your feelings” (social sharing). These strategies have been assessed in previous ESM studies (Brans et al., 2013; Hartley et al., 2014; Kiekens et al., 2023, 2023; Medland et al., 2020). With 10 daily assessments over 7 days, the maximum possible number of measurements for emotion regulation strategies was 70. Reliability was satisfactory for emotion regulation strategies (.53).

Dataset 3: 3-wave longitudinal study, KU Leuven (main reference: Erbas et al., 2018)

Participants

Participants were undergraduates from the University of Leuven, Belgium. This three-wave study was approved by the ethics committee of the University of Leuven. Here, we only used the data from the first wave collected in 2012. 686 first-year undergraduates completed the Center for Epidemiologic Studies Depression Scale (CES-D, Radloff, 1977) as a prescreening questionnaire. 180 participants, formed by equal number of participants from four quartiles of the CES-D distribution, were selected following a stratified sampling approach. An additional 22 participants took part without completing the CES-D, resulting in a total of 202 participants. There were no participants excluded based on

reaction time because reaction time was not available for ESM assessments in this dataset. No participants had zero variance across all ESM items, so the final sample was 202 participants. Mean age of the sample was 18.32 years (SD = 0.96), and 55% were women. Majority of the sample had Belgian nationality (93%).

Procedure

The participants took part in an introductory session in the laboratory and filled out questionnaires unrelated to the current study. Then, they received standardized devices (Motorola Defy Plus) with custom-built ESM software installed and were trained to use the phone to complete the ESM questionnaires. Participants practiced filling the ESM questionnaire and could clarify with an experimenter before leaving the lab. The ESM study lasted for 7 consecutive days, during which 10 beeps occurred semi-randomly each day in a 12-hr time frame. Participants were informed that completing one measurement would take an average of 1-2 minutes. Participants had 90 seconds to answer each question once they opened the questionnaire before it timed out. There were no reminders for participants in case they did not open the momentary assessments. Participants answered 87.27% of the beeps (SD = 9.05%, range: 67–100% of all beeps). The participants were reimbursed with 60 Euros for this wave of study. They were eligible for an extra 60 EUR reimbursement for completing all three waves of study.

ESM measures

Emotions. At each momentary assessment, participants rated three positive emotions (happy, relaxed, cheerful) and six negative emotions (lonely, angry, anxious, sad, depressed, and stressed) presented on a slider scale from 0 (not at all) to 100 (very much). The stem for these items was “How [emotion] do you feel at the moment?” These items have been used in other ESM studies (Achterhof et al., 2022; Bakker et al., 2019; Barge-Schaapveld et al., 1999; Bastiaansen et al., 2018; Bennik, 2015; Brans et al., 2013; Bülow et al., 2022; Delespaul & DeVries, 1987; Fried et al., 2022; Hasmi et al., 2017; Jacobs

et al., 2007; Kiekens et al., 2023; Myin-Germeys et al., 2000; Rauschenberg et al., 2017; Schneiders et al., 2006). With 10 daily assessments over 7 days, the maximum possible number of measurements for negative and positive emotions was 70. Reliability was satisfactory for positive emotions (.74) and negative emotions (.73).

Emotion regulation strategies. At each momentary assessment, participants rated the extent they used six emotion regulation strategies presented on a slider scale from 0 (not at all) to 100 (almost all the time). The stem for these items was "Since the last beep, have you…" and ended with "viewed the cause of your feelings from a different perspective?" (cognitive reappraisal), "suppressed the expression of your feelings" (expressive suppression), "distracted your attention away from your feelings" (distraction), "talked about your feelings with others" (social sharing), "brooded about something in the past" (rumination) and "brooded about something in the future" (worry). These strategies have been assessed in previous ESM studies (Achterhof et al., 2022; Bastiaansen et al., 2018; Brans et al., 2013; Hartley et al., 2014; Kiekens et al., 2023; Medland et al., 2020). With 10 daily assessments over 7 days, the maximum possible number of measurements for emotion regulation strategies was 70. Reliability was satisfactory for emotion regulation strategies (.52).

Dataset 4: Emotion regulation in daily life, Tilburg University (main reference: Van Roekel & Trompetter, 2023)

Participants

Participants were undergraduates from Tilburg University, the Netherlands. This study was approved by the ethics committee of the Tilburg School of Social and Behavioral Sciences (protocol number: EC-2017.95). Data were collected in 2018. 242 first-year undergraduates who needed to earn course credits were recruited. For this study, only data from participants who were younger than 25 years old were used. Therefore, the initial sample consisted of 179 participants (age M = 20.84 , *SD* = 1.67). After excluding

participants who had zero variance across all ESM items, there was a final sample of 178 participants. There were no participants excluded based on reaction time because reaction time was not available for ESM assessments in this dataset. Mean age of the sample was 20.85 years (SD = 1.67), and 78% were women. Majority of the sample was born in the Netherlands (93%).

Procedure

Participants were recruited through the University course credit system, where they were able to read information about the research and could register via the same system. To participate, students had to click a link in an information letter sent to them by email. There, they signed informed consent and completed a questionnaire with baseline data that were not relevant for this study. The email also instructed participants to download the app "Ethica" (www.ethicadata.com) on their smartphone for the ESM assessments. The ESM period started within a few days after completing the baseline questionnaires. The ESM study lasted for 14 consecutive days, during which the Ethica app gave 5 beeps quasi-randomly each day in a 12-hr time frame. The participants had to complete the questionnaire within 30 minutes after the notification. Participants were informed that completing one measurement would take an average of 3 minutes. In cases where participants did not open the momentary assessments, the app sent a reminder after the initial notification, but the details of the notification setting were lost due to interface change of Ethica. The median number of completed assessments per participant was 52 out of 70 (73.97%, M = 66.36% , SD = 23.50%, range: 5.35–98.63% of all beeps). When the 14 days were over, the study was completed and the participants were rewarded with 4 test credits for participants recruited via the Tilburg course credit system or a chance of winning 30-Euro shopping vouchers for participants recruited via other channels.

ESM measures

Emotions. At each momentary assessment, participants rated seven positive emotions (enthusiastic, content, energetic, calm, powerful, cheerful, and grateful) and six negative emotions (irritated, bored, nervous, sad, angry, and depressed) presented on a slider scale from 0 (not at all) to 100 (very much). The stem for these items was "I now feel (right before the beep went off) [emotion]." These items have been used in other ESM studies (Achterhof et al., 2022; Bakker et al., 2019; Barge-Schaapveld et al., 1999; Bastiaansen et al., 2018; Bennik, 2015; Bülow et al., 2022; Delespaul & DeVries, 1987; Fried et al., 2022; Hasmi et al., 2017; Jacobs et al., 2007; Kiekens et al., 2023; Myin-Germeys et al., 2000; Rauschenberg et al., 2017; Schneiders et al., 2006; Spence et al., 2014). With 10 daily assessments over 7 days, the maximum possible number of measurements for negative and positive emotions was 70. Reliability was satisfactory for positive emotions (.80) and negative emotions (.69).

Emotion regulation strategies. At each momentary assessment, participants rated the extent they used seven emotion regulation strategies presented on a slider scale from 0 (not at all) to 100 (very much). Based on theoretical frameworks of Parkinson and Totterdell (1999) and Aldao et al. (2010), the stem for these items was "Indicate to what extent you have used each of the following strategies since the last beep, regardless of whether they helped. To change my negative emotions, I have…" and ended with "addressed the situation that caused my emotions or have made plans for addressing it" (problem solving), "brooded my emotions with others" (co-brooding), "sought distraction" (distraction), "suppressed, ignored or avoided (the thoughts about) my emotions or the situation that caused them." (avoidance), "talked about my feelings with others for advice or support" (social sharing), "been thinking about my feelings and their causes and/or consequences" (rumination) and "experienced my emotions as they are without wanting them change: it is OK that they are there" (acceptance). Rumination, acceptance, social sharing, and distraction have been assessed in previous ESM studies (Achterhof et al.,

2022; Kiekens et al., 2023). With 10 daily assessments over 7 days, the maximum possible number of measurements for emotion regulation strategies was 70. Reliability was satisfactory for emotion regulation strategies (.53).

Dataset 5: Outside-in, Ghent University (main reference: Braet et al., 2023)

Participants

244 students were recruited from local schools in Belgium (age M = 13.46, SD = 0.42; female = 48%). This 3-wave study was approved by the Medical Ethics Committee of Ghent University Hospital (protocol number: BC-09559). For our analysis, we only utilized data from the third wave, which was collected in 2022. This choice ensures that participants from this study have a closer age range to participants in other studies. After excluding observations in which each ESM item was completed in less than 500ms and excluding participants who showed zero variance across all ESM items, the final sample consisted of 212 participants. Mean age of the sample was 13.46 years (SD = 0.42), and 44% were female. Majority of the sample were born to Belgian parents (90%).

Procedure

Participants were recruited through nine different schools (Flanders region). Parental consent and written assent from adolescents were obtained. All participants installed the m-Path app on their smartphones (www.m-path.io, Mestdagh et al., 2023). The ESM period started within a few days after completing different baseline questionnaires. The ESM study lasted for 14 consecutive days during school weeks, during which the m-path app gave 5 beeps at fixed intervals each day in a 12-hr time frame. One measurement took an average of 2 minutes. The participants had 50 to 120 minutes after the notification to complete the questionnaire (first to third beep of the day: 50 minutes, fourth beep of the day: 90 minutes, and last beep of the day: 120 minutes). In cases where participants did not open the momentary assessments, the app sent reminders every 10 minutes after the initial notification. Compliance rate was also monitored during the study

for each participant, after two days of low compliance participants received a message via m-path. Out of all participants, one discontinued the study after seven days, thus only receiving 35 beeps. Two participants encountered technical issues that prevented them from receiving some beeps on weekends, resulting in only 52 and 56 beeps received. Another 27 participants experienced occasional technical issues, receiving 65 to 69 beeps over the course of 14 days. The median number of assessments completed per participant was 49 out of 70 (70%, M = 64.51% , SD = 24.97%, range: 1.4%–100% of all possible beeps). When the 14 days were over, the study was completed and the participants were rewarded with a gift voucher worth €20 when they completed at least 70% of surveys, while a voucher of €10 was given to those who completed between 50% and 70% of surveys.

ESM measures

Emotions. At each momentary assessment, participants rated three positive emotions (happy, energetic, and relaxed) and six negative emotions (sad, angry, anxious, uncertain, annoyed, and stressed) presented on a 7-point scale from 1 (totally not) to 7 (totally). The stem for these items was "I now feel: [emotion]." These items have been used in other ESM studies (Achterhof et al., 2022; Bakker et al., 2019; Barge-Schaapveld et al., 1999; Bastiaansen et al., 2018; Bennik, 2015; Brans et al., 2013; Bülow et al., 2022; Delespaul & DeVries, 1987; Hasmi et al., 2017; Jacobs et al., 2007; Kiekens et al., 2023; Myin-Germeys et al., 2000; Rauschenberg et al., 2017; Schneiders et al., 2006). With 5 daily assessments over 14 days, the maximum possible number of measurements for negative and positive emotions was 70. Reliability was satisfactory for positive emotions (.60) and negative emotions (.69).

Emotion regulation strategies. First, participants reported the intensity of their experienced negative emotions since the last survey (or after waking up). In case no negative emotion was experienced, participants were instructed to respond with a score of 1. Then, Participants rated the extent they used eight emotion regulation strategies

presented on a 7-point scale from 1 (totally not) to 7 (totally). The stem for these items was "When I felt those negative emotions...." With reference to Medland et al. (2020), five items ended with "I tried to see the situation in other ways" (cognitive reappraisal), "I tried to hide my emotions" (expressive suppression), "I did things to distract myself" (distraction), "I could not stop thinking about them" (rumination), and "I tried to express my emotions" (expression). Next, one item was added to assess social sharing, "I talked with someone else about the situation" (social sharing). These strategies have been assessed in previous ESM studies (Achterhof et al., 2022; Bastiaansen et al., 2018; Brans et al., 2013; Hartley et al., 2014; Kiekens et al., 2023). Finally, based on Berking and Znoj (2011), two more self-compassion items were included: "I have supported myself" (self-compassion) and "I tried to cheer up myself" (self-compassion). With 5 daily assessments over 14 days, the maximum possible number of measurements for emotion regulation strategies was 70. Reliability was satisfactory for emotion regulation strategies (.72).

Supplemental Materials 3: Distributions, Descriptive Statistics and Correlations of Measures

Distrbutions of Momentary Indices

We visually inspected the distributions of within-person means, standard deviations, and skewness values of all momentary indices calculated from ESM measures (Figure S3). All indices have comparable means and standard deviations with earlier ESM studies that reported emotion intensity (Bennik, 2015; Bülow et al., 2022; Jacobs et al., 2007; Rauschenberg et al., 2017; Schneiders et al., 2006), emotion differentiation (Emery et al., 2022; Erbas et al., 2021; Knapp et al., 2024; Lischetzke et al., 2021), and emotion regulation variability (Lo et al., 2024).

Referencing to von Klipstein et al. (2023)'s procedures in assessing potential floor or ceiling effects, we noticed that negative emotion intensity, emotion regulation intensity, and the strategy switching subcomponent of emotion regulation variability have some mean values close to the lower bound of the scale, indicating potential floor effects. We further checked the proportion of zero values in these indices across persons and across measurements (Table S3). Across all ESM measurements, 18.62% of ratings for negative emotion intensity and 15.02% for emotion regulation intensity were zero. However, these percentages are significantly lower than the 51.7% zero-rating proportion reported in von Klipstein et al. (2023), with which they demonstrated a floor effect in negative emotion intensity in their sample. The comparatively lower proportions in our samples suggest a lesser extent of floor effects, if present at all. Despite the potential floor effects, the distribution of negative emotion differentiation is comparable to that of positive emotion differentiation, originating from normally distributed positive emotion intensities. Moreover, emotion regulation variability calculations inherently control for emotion regulation intensity, protecting against floor effects. This is evident from a very low proportion of zero values across adolescents and ESM measurements in emotion regulation

variability. Interestingly, the strategy switching subcomponent of emotion regulation variabiliity has the highest proportion of floored within-person mean and SD, and is among the indices with highest proportion of floored values at ESM measurement-level. So, for some adolescents, their emotion regulation variability is solely comprised of the endorsement change subcomponent. This means that these adolescents varied the *intensity* of the same strategies but seldom change varied their strategy *selection*. Our confirmatory hypotheses primarily focused on negative emotion differentiation and the full index of emotion regulation variability. Based on the observed distribution patterns, we deemed it appropriate to use these indices for testing the confirmatory hypotheses. That said, our exploratory analyses included negative emotion intensity as an outcome variable. To address this, in Supplemental Materials 8, we conducted sensitivity analyses to examine whether the presence of zero emotion (regulation) intensity moderated the effects tested in our study.

Table S3

Proportion of Zero Values on Momentary Indices Across Persons and Across ESM Measurements

Momentary index	Within-person Mean	Within-person SD	Across all ESM Measurements
Positive emotion intensity	0.00%	0.13%	0.95%
Positive emotion differentiation	0.00%	0.00%	0.03%
Negative emotion intensity	0.00%	0.00%	18.62%
Negative emotion differentiation	0.00%	0.13%	0.03%
Emotion regulation intensity	0.00%	0.00%	15.02%
Emotion regulation variability (full index)	0.52%	0.91%	0.01%
Endorsement change subcomponent	0.52%	0.91%	0.01%
Strategy switching subcomponent	4.52%	4.55%	15.89%

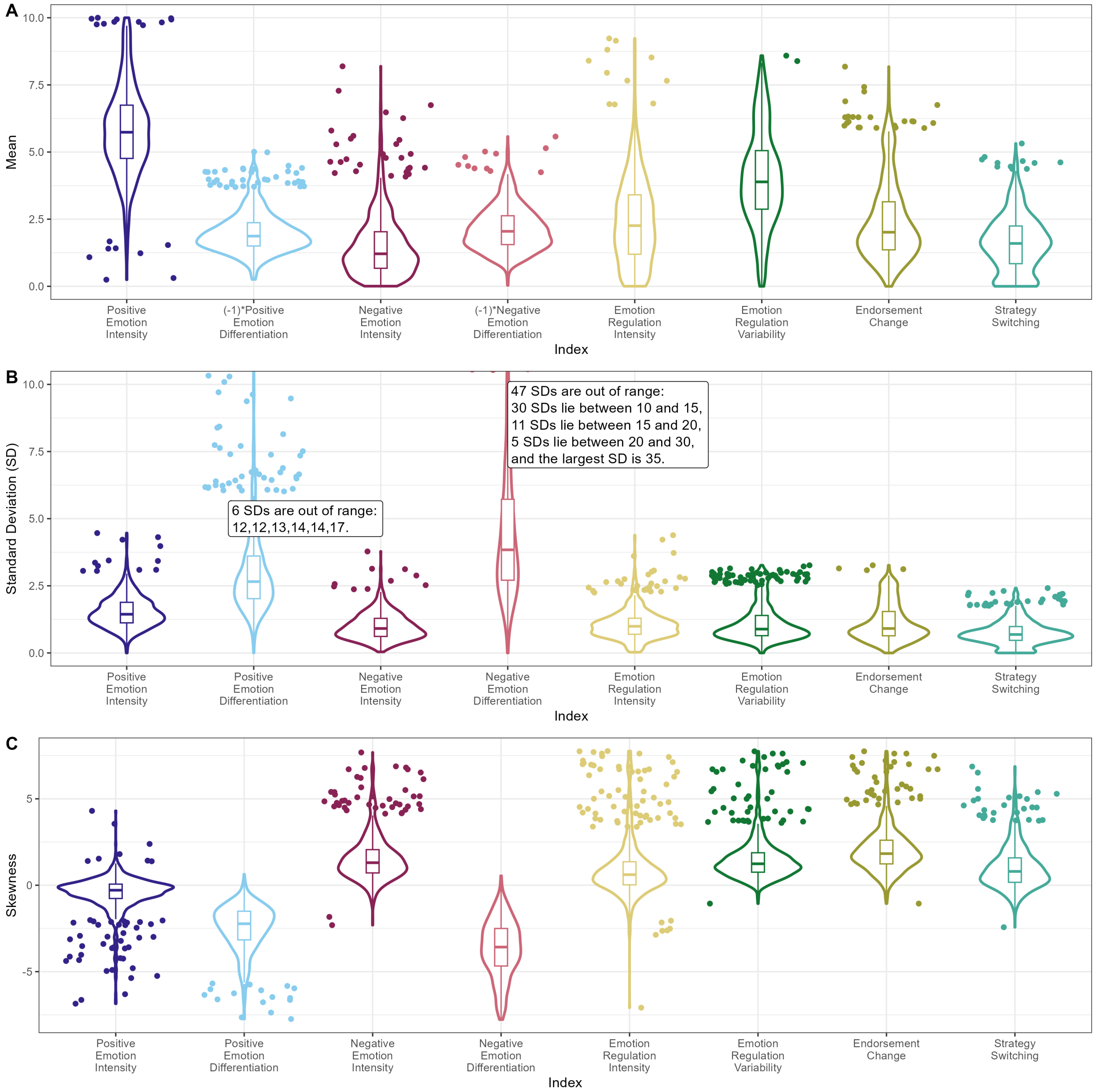

Figure S3

Combined violin plots and box plots of the within-person means (M; Panel A), within-person standard deviations (SD; Panel B), and within-person skewness (Panel C) of momentary indices derived from ESM measurements. The outer shapes represent the mirrored density function, encompassing box plots. The thick central line in the box plot marks the median, while the bottom and top edges of the rectangle show the 25th and 75th percentiles, respectively. Vertical lines stretch beyond these percentiles to a maximum of 1.5 times the inter-quartile range, and dots represent values outside this range of the vertical lines. Note that in Panel A, we inverted the negative values of means of positive and negative emotion differentiation to positive values to ease comparison.

Descriptive Statistics and Correlations of Measures

We further inspected the descriptive statistics, within-person correlations and between-person correlations of momentary indices (Table S3.1 to S3.2.5) and ESM measures (Table S3.3.1 to S3.3.5). First, between positive and negative emotion intensity, there were negative within-person and between-person correlations, matching previous ESM studies that reported such negative within-person correlations (Springstein et al., 2023) and between-person correlations (Schneiders et al., 2006; van Eck et al., 1998). Second, between any pairs of positive/negative emotion differentiation and positive/negative emotion intensity, their within-person and between-person correlations matched in directions and were of comparable strengths with previous ESM studies that reported such correlations (Knapp et al., 2024; Lischetzke et al., 2021; Springstein et al., 2023). Third, between negative emotion intensity and emotion regulation variability, although within-person correlations were not consistent in directions across datasets, negative between-person correlations between negative emotion intensity and emotion regulation variability (and its endorsement subcomponent) matched previous reports (Lo et al., 2024). Overall, correlations between momentary indices in our pooled dataset were generally in line with previous ESM studies, supporting us to further analyze these indices.

Table S3.1

Descriptive Statistics, Within- and Between-person Correlations of Momentary Indices in the Pooled Dataset (N=778)

Variable(Index/Measure)	n	M	SDw	SDb	Min	Max	1	2	3	4	5	6	7	8
1. Positive emotion intensity	39286	5.78	1.65	1.53	2.16	8.54		.27	-.44	.14	-.03	-.12	.03	-.24
								[.20, .33]	[-.50,-.39]	[.07, .21]	[-.10, .05]	[-.19,-.05]	[-.04, .10]	[-.31,-.18]
2. Positive emotion differentiation	39230	-1.98	0.76	3.06	-15.25	-0.03	.23		-.10	.24	-.02	.00	.03	-.05
							[.22, .24]		[-.16,-.02]	[.17, .30]	[-.10, .05]	[-.07, .07]	[-.04, .10]	[-.12, .02]
3. Negative emotion intensity	39179	1.46	1.16	0.98	0.3	4.57	-.45	-.19		-.26	.41	-.10	-.20	.11
							[-.46,-.44]	[-.20,-.18]		[-.32,-.19]	[.35, .47]	[-.17,-.03]	[-.26,-.13]	[.04, .18]
4. Negative emotion differentiation	39179	-2.15	0.82	4.8	-28.26	-0.03	.22	.28	-.51		-.07	-.02	-.04	.03
							[.21, .23]	[.28, .29]	[-.52,-.50]		[-.14, .00]	[-.09, .05]	[-.11, .03]	[-.04, .10]
5. Emotion regulation intensity	36383	2.28	1.62	1.06	0.78	5.08	-.10	-.06	.28	-.16		-.24	-.40	.14
							[-.11,-.09]	[-.07,-.05]	[.27, .29]	[-.17,-.15]		[-.31,-.17]	[-.45,-.34]	[.07, .21]
6. Emotion regulation variability	36218	4.03	1.78	1.13	3.04	7.29	-.03	-.11	.06	-.15	-.04		.81	.57
							[-.04,-.02]	[-.12,-.10]	[.05, .07]	[-.16,-.14]	[-.05,-.03]		[.79, .83]	[.52, .61]
7. Endorsement change	36218	2.35	1.47	1.13	1.5	6.12	-.01	-.07	.04	-.13	-.04	.76		-.02
							[-.02, .00]	[-.08,-.06]	[.03, .05]	[-.14,-.12]	[-.05,-.03]	[.75, .76]		[-.09, .05]
8. Strategy switching	36218	1.68	1.05	0.75	0.38	3.65	-.03	-.06	.03	-.02	-.01	.34	-.36	
							[-.04,-.02]	[-.07,-.05]	[.02, .04]	[-.03,-.01]	[-.02, .00]	[.33, .35]	[-.36,-.35]	

Note: SDw: Within-person SD. SDb: Between-person SD. Min: mean of minimum rating. Max: mean of maximum rating. Within-person correlations at lower triangle and between-person correlations at upper triangle. Confidence interval of correlations in squared brackets. All these indices were calculated only in observations with no missingness in relevant ESM items, so the lower n for emotion regulation indices reflected more missing items in constituent ESM items.

Table S3.2.1

Descriptive Statistics, Within- and Between-person Correlations of Momentary Indices in Dataset 1: G(F)ood together (Radboud)

Variable(Index/Measure)	n	M	SDw	SDb	Min	Max	1	2	3	4	5	6	7	8
1. Positive emotion intensity	3384	6.76	1.19	1.14	3.97	8.64		.39	-.64	.41	.00	-.07	.03	-.18
								[.19, .56]	[-.75,-.49]	[.21, .57]	[-.22, .22]	[-.28, .16]	[-.19, .25]	[-.38, .04]
2. Positive emotion differentiation	3384	-1.92	0.61	2.78	-13.62	-0.02	.30		-.38	.53	-.04	-.13	-.04	-.13
							[.27, .33]		[-.55,-.18]	[.35, .67]	[-.26, .18]	[-.34, .10]	[-.26, .18]	[-.34, .09]
3. Negative emotion intensity	3331	1.29	1.13	0.9	0.23	3.8	-.54	-.23		-.35	.09	-.07	-.16	.20
							[-.56,-.51]	[-.27,-.20]		[-.52,-.14]	[-.13, .30]	[-.28, .16]	[-.37, .06]	[-.02, .40]
4. Negative emotion differentiation	3331	-1.81	0.68	3.41	-18.09	-0.03	.28	.34	-.50		.10	-.17	-.07	-.17
							[.24, .31]	[.31, .37]	[-.53,-.47]		[-.12, .32]	[-.38, .05]	[-.28, .16]	[-.38, .05]
5. Emotion regulation intensity	583	3.48	1.48	1.58	1.67	5.78	-.16	-.14	.22	-.12		-.52	-.61	.28
							[-.24,-.08]	[-.22,-.06]	[.14, .30]	[-.21,-.04]		[-.67,-.34]	[-.73,-.45]	[.06, .47]
6. Emotion regulation variability	583	4.28	1.87	1.21	3.38	6.55	.00	-.04	-.01	-.03	-.20		.85	.07
							[-.09, .08]	[-.12, .04]	[-.09, .08]	[-.12, .05]	[-.28,-.13]		[.78, .90]	[-.15, .28]
7. Endorsement change	583	2.93	2.11	1.13	2.17	5.16	.04	.02	-.04	-.01	-.26	.83		-.46
							[-.05, .12]	[-.07, .10]	[-.12, .05]	[-.10, .07]	[-.34,-.19]	[.80, .85]		[-.62,-.27]
8. Strategy switching	583	1.34	1.11	0.67	0.63	2.5	-.07	-.09	.05	-.03	.10	.28	-.31	
							[-.15, .02]	[-.17,-.01]	[-.03, .13]	[-.12, .05]	[.02, .18]	[.20, .35]	[-.39,-.24]	

Note: SDw: Within-person SD. SDb: Between-person SD. Min: mean of minimum rating. Max: mean of maximum rating. Within-person correlations at lower triangle and between-person correlations at upper triangle. Confidence interval of correlations in squared brackets. All these indices were calculated only in observations with no missingness in relevant ESM items, so the lower n for emotion regulation indices reflected more missing items in constituent ESM items.

Table S3.2.2

Descriptive Statistics, Within- and Between-person Correlations of Momentary Indices in Dataset 2: Emotions in daily life (Leuven)

Variable(Index/Measure)	n	M	SDw	SDb	Min	Max	1	2	3	4	5	6	7	8
1. Positive emotion intensity	5816	5.67	1.32	1.75	1.62	8.96		.16	-.62	.21	-.23	.18	.21	.07
								[-.04, .35]	[-.73,-.48]	[.01, .40]	[-.41,-.03]	[-.02, .36]	[.01, .39]	[-.13, .27]
2. Positive emotion differentiation	5816	-1.49	0.2	2.05	-10.47	0	.21		-.04	.27	-.10	.00	.04	-.04
							[.19, .24]		[-.24, .16]	[.07, .44]	[-.29, .10]	[-.20, .20]	[-.16, .24]	[-.24, .16]
3. Negative emotion intensity	5814	1.47	1.08	0.99	0.24	4.79	-.48	-.17		-.41	.61	-.50	-.48	-.32
							[-.50,-.46]	[-.20,-.15]		[-.57,-.23]	[.47, .72]	[-.64,-.33]	[-.62,-.31]	[-.49,-.13]
4. Negative emotion differentiation	5814	-2.05	0.48	4.8	-30.11	0	.26	.33	-.54		-.30	.13	.11	.09
							[.24, .29]	[.31, .35]	[-.56,-.52]		[-.47,-.11]	[-.08, .32]	[-.09, .31]	[-.11, .28]
5. Emotion regulation intensity	5815	2.32	1.06	1	0.63	5.2	-.14	-.07	.37	-.24		-.66	-.72	-.33
							[-.17,-.12]	[-.09,-.04]	[.35, .40]	[-.26,-.21]		[-.76,-.53]	[-.80,-.61]	[-.49,-.14]
6. Emotion regulation variability	5815	4.48	1.48	0.85	3.44	7.13	.03	-.08	-.06	-.09	-.19		.83	.78
							[.01, .06]	[-.10,-.05]	[-.09,-.04]	[-.11,-.06]	[-.21,-.16]		[.75, .88]	[.69, .85]
7. Endorsement change	5815	2.32	0.96	0.93	1.28	5.8	.01	.00	.00	-.10	-.05	.54		.30
							[-.01, .04]	[-.03, .02]	[-.03, .02]	[-.13,-.08]	[-.08,-.03]	[.52, .56]		[.11, .47]
8. Strategy switching	5815	2.17	0.87	0.87	0.44	4.66	.02	-.07	-.06	.03	-.13	.40	-.55	
							[-.01, .04]	[-.10,-.05]	[-.09,-.04]	[.00, .05]	[-.15,-.10]	[.38, .42]	[-.57,-.53]	

Note: SDw: Within-person SD. SDb: Between-person SD. Min: mean of minimum rating. Max: mean of maximum rating. Within-person correlations at lower triangle and between-person correlations at upper triangle. Confidence interval of correlations in squared brackets. All these indices were calculated only in observations with no missingness in relevant ESM items, so the lower n for emotion regulation indices reflected more missing items in constituent ESM items.

Table S3.2.3

Descriptive Statistics, Within- and Between-person Correlations of Momentary Indices in Dataset 3: 3-wave longitudinal study (Leuven)

Variable(Index/Measure)	n	M	SDw	SDb	Min	Max	1	2	3	4	5	6	7	8
1. Positive emotion intensity	12346	5.69	1	1.63	1.85	8.94		.21	-.45	.25	-.28	.13	.21	-.05
								[.07, .34]	[-.55,-.33]	[.11, .37]	[-.40,-.15]	[.00, .27]	[.07, .33]	[-.19, .09]
2. Positive emotion differentiation	12346	-1.88	0.36	2.63	-13.19	0	.15		-.22	.27	-.22	.10	.16	-.05
							[.13, .17]		[-.35,-.09]	[.14, .39]	[-.35,-.08]	[-.04, .23]	[.02, .29]	[-.19, .09]
3. Negative emotion intensity	12346	1.48	0.88	0.94	0.26	4.65	-.53	-.22		-.40	.72	-.51	-.53	-.19
							[-.55,-.52]	[-.24,-.20]		[-.51,-.28]	[.64, .78]	[-.61,-.40]	[-.62,-.42]	[-.32,-.05]
4. Negative emotion differentiation	12346	-2.3	0.81	5.07	-31.81	0	.24	.33	-.54		-.37	.20	.20	.10
							[.22, .25]	[.31, .34]	[-.55,-.53]		[-.48,-.25]	[.07, .33]	[.06, .32]	[-.04, .24]
5. Emotion regulation intensity	12346	2.11	1.13	0.96	0.5	4.86	-.17	-.08	.34	-.20		-.61	-.70	-.13
							[-.19,-.15]	[-.10,-.06]	[.32, .35]	[-.22,-.19]		[-.69,-.52]	[-.76,-.62]	[-.26, .01]
6. Emotion regulation variability	12346	4.57	1.6	0.95	3.51	7.44	.03	-.09	-.01	-.09	-.18		.86	.63
							[.02, .05]	[-.11,-.07]	[-.03, .01]	[-.11,-.08]	[-.20,-.16]		[.82, .89]	[.54, .71]
7. Endorsement change	12346	2.6	1.25	1.06	1.51	6.39	.03	-.04	.00	-.09	-.13	.57		.15
							[.01, .04]	[-.06,-.02]	[-.01, .02]	[-.10,-.07]	[-.15,-.12]	[.56, .59]		[.02, .29]
8. Strategy switching	12346	1.96	0.82	0.93	0.31	4.67	.00	-.05	-.02	.00	-.03	.38	-.54	
							[-.01, .02]	[-.07,-.03]	[-.03, .00]	[-.02, .02]	[-.05,-.02]	[.37, .40]	[-.55,-.53]	

Note: SDw: Within-person SD. SDb: Between-person SD. Min: mean of minimum rating. Max: mean of maximum rating. Within-person correlations at lower triangle and between-person correlations at upper triangle. Confidence interval of correlations in squared brackets. All these indices were calculated only in observations with no missingness in relevant ESM items, so the lower n for emotion regulation indices reflected more missing items in constituent ESM items.

Table S3.2.4

Descriptive Statistics, Within- and Between-person Correlations of Momentary Indices in Dataset 4: Emotion regulation in daily life (Tilburg)

Variable(Index/Measure)	n	M	SDw	SDb	Min	Max	1	2	3	4	5	6	7	8
1. Positive emotion intensity	7904	4.58	1.17	1.28	1.95	7.09		-.06	-.20	.00	.19	-.13	-.14	-.02
								[-.20, .09]	[-.34,-.05]	[-.15, .15]	[.04, .33]	[-.27, .02]	[-.28, .01]	[-.17, .12]
2. Positive emotion differentiation	7904	-2.95	0.79	3.94	-18.14	-0.06	.13		-.01	.32	-.10	-.16	-.13	-.09
							[.11, .15]		[-.16, .14]	[.19, .45]	[-.25, .05]	[-.30,-.01]	[-.27, .02]	[-.23, .06]
3. Negative emotion intensity	7852	1.54	0.93	0.92	0.45	4.32	-.47	-.21		-.32	.63	-.29	-.31	-.07
							[-.49,-.46]	[-.23,-.18]		[-.44,-.18]	[.53, .71]	[-.42,-.15]	[-.44,-.17]	[-.21, .08]
4. Negative emotion differentiation	7852	-2.15	0.8	4.31	-23.6	-0.02	.27	.33	-.57		-.31	.09	.09	.03
							[.25, .29]	[.31, .35]	[-.58,-.55]		[-.44,-.17]	[-.06, .24]	[-.06, .24]	[-.12, .17]
5. Emotion regulation intensity	7802	2.32	1.08	0.9	0.92	4.71	.00	-.05	.25	-.16		-.41	-.55	.07
							[-.03, .02]	[-.07,-.03]	[.23, .27]	[-.18,-.14]		[-.53,-.28]	[-.65,-.44]	[-.08, .21]
6. Emotion regulation variability	7637	3.88	1.43	0.86	2.89	6.22	-.08	-.15	.08	-.18	-.03		.81	.58
							[-.10,-.06]	[-.17,-.12]	[.06, .11]	[-.20,-.15]	[-.05,-.01]		[.75, .86]	[.48, .67]
7. Endorsement change	7637	2.13	1.19	0.84	1.25	4.79	.00	-.06	.00	-.09	-.08	.62		.00
							[-.02, .03]	[-.08,-.03]	[-.03, .02]	[-.11,-.07]	[-.10,-.06]	[.60, .63]		[-.15, .15]
8. Strategy switching	7637	1.75	0.87	0.76	0.56	3.88	-.10	-.11	.10	-.10	.05	.46	-.41	
							[-.12,-.08]	[-.13,-.08]	[.08, .12]	[-.12,-.08]	[.03, .07]	[.44, .48]	[-.43,-.40]	

Note: SDw: Within-person SD. SDb: Between-person SD. Min: mean of minimum rating. Max: mean of maximum rating. Within-person correlations at lower triangle and between-person correlations at upper triangle. Confidence interval of correlations in squared brackets. All these indices were calculated only in observations with no missingness in relevant ESM items, so the lower n for emotion regulation indices reflected more missing items in constituent ESM items.

Table S3.2.5

Descriptive Statistics, Within- and Between-person Correlations of Momentary Indices in Dataset 5: Outside-in (Ghent)

**Variable(Index/Measure)**	**n**	**M**	**SDw**	**SDb**	**Min**	**Max**	**1**	**2**	**3**	**4**	**5**	**6**	**7**	**8**
1. Positive emotion intensity	9836	6.58	2.11	1.7	2.19	9.12		-.09	-.51	.09	-.08	-.27	-.13	-.30
								[-.23, .04]	[-.60,-.40]	[-.04, .22]	[-.21, .05]	[-.39,-.14]	[-.26, .00]	[-.42,-.17]
2. Positive emotion differentiation	9780	-1.63	0.55	3.3	-17.55	-0.05	.36		-.05	.37	.05	-.03	-.12	.13
							[.35, .38]		[-.18, .08]	[.25, .48]	[-.08, .18]	[-.16, .11]	[-.25, .02]	[.00, .26]
3. Negative emotion intensity	9836	1.42	1.55	1.11	0.27	4.91	-.33	-.17		-.14	.37	.22	.01	.39
							[-.35,-.31]	[-.19,-.15]		[-.27,-.01]	[.25, .48]	[.09, .34]	[-.13, .14]	[.27, .50]
4. Negative emotion differentiation	9836	-2.15	0.96	5.48	-31.81	-0.06	.17	.24	-.45		.02	-.18	-.27	.07
							[.15, .19]	[.22, .26]	[-.46,-.43]		[-.11, .15]	[-.31,-.05]	[-.39,-.14]	[-.06, .20]
5. Emotion regulation intensity	9837	2.35	2.3	1.1	0.68	5.27	-.06	-.04	.21	-.10		.01	-.27	.42
							[-.08,-.04]	[-.06,-.02]	[.19, .23]	[-.12,-.08]		[-.13, .14]	[-.39,-.14]	[.30, .52]
6. Emotion regulation variability	9837	3.19	2.05	1.62	2.42	8.36	-.09	-.11	.14	-.19	.08		.84	.57
							[-.11,-.07]	[-.13,-.09]	[.12, .15]	[-.21,-.17]	[.06, .10]		[.79, .87]	[.47, .65]
7. Endorsement change	9837	2.2	1.69	1.53	1.55	7.45	-.06	-.10	.10	-.19	.06	.92		.03
							[-.08,-.04]	[-.12,-.08]	[.08, .12]	[-.20,-.17]	[.04, .08]	[.92, .92]		[-.11, .16]
8. Strategy switching	9837	0.99	1.12	0.56	0.2	2.51	-.08	-.03	.10	-.04	.06	.31	-.09	
							[-.10,-.06]	[-.05,-.01]	[.08, .12]	[-.06,-.02]	[.04, .08]	[.29, .33]	[-.11,-.07]	

Note: SDw: Within-person SD. SDb: Between-person SD. Min: mean of minimum rating. Max: mean of maximum rating. Within-person correlations at lower triangle and between-person correlations at upper triangle. Confidence interval of correlations in squared brackets. All these indices were calculated only in observations with no missingness in relevant ESM items, so the lower n for emotion regulation indices reflected more missing items in constituent ESM items.

Table S3.3.1.1

Descriptive Statistics, Within- and Between-person Correlations of Positive Emotions in Dataset 1: G(F)ood together (Radboud)

Variable(Index/Measure)	n	M	SDw	SDb	Min	Max	1	2	3	4
1. Content	3489	7.12	1.27	1.39	3.17	9.18		.83	.90	.67
								[.75,.89]	[.85,.94]	[.53,.77]
2. Relaxed	3498	6.64	1.34	1.79	2.11	9.17	.38		.77	.58
							[.35,.41]		[.66,.84]	[.42,.71]
3. Joyful	3498	7.08	1.28	1.43	3.18	9.23	.50	.36		.72
							[.48,.53]	[.33,.39]		[.59,.81]
4. Energetic	3487	6.19	1.41	1.75	2.02	8.89	.35	.22	.47	
							[.32,.38]	[.18,.25]	[.44,.49]	

Note: SDw: Within-person SD. SDb: Between-person SD. Min: mean of minimum rating. Max: mean of maximum rating. Within-person correlations at lower triangle and between-person correlations at upper triangle. Confidence interval of correlations in squared brackets. All these ESM measures were calculated only in observations with no missingness.

Table S3.3.1.2

Descriptive Statistics, Within- and Between-person Correlations of Negative Emotions in Dataset 1: G(F)ood together (Radboud)

Variable(Index/Measure)	n	M	SDw	SDb	Min	Max	1	2	3	4	5
1. Irritated	3483	1.41	1.23	1.58	0.07	6.36		.45	.54	.56	.42
								[.26,.61]	[.36,.68]	[.40,.70]	[.22,.58]
2. Worried	3493	1.52	1.36	1.51	0.12	5.87	.24		.79	.67	.58
							[.21,.27]		[.70,.86]	[.53,.78]	[.42,.71]
3. Depressed	3487	1.14	1.24	1.25	0.13	4.76	.30	.33		.69	.76
							[.27,.33]	[.30,.36]		[.56,.79]	[.65,.84]
4. Insecure	3492	1.44	1.8	1.17	0.22	4.75	.23	.37	.40		.74
							[.19,.26]	[.34,.40]	[.37,.43]		[.62,.82]
5. Lonely	3483	0.96	1.1	1.2	0.04	4.69	.17	.19	.36	.32	
							[.13,.20]	[.16,.22]	[.33,.39]	[.29,.35]	

Note: SDw: Within-person SD. SDb: Between-person SD. Min: mean of minimum rating. Max: mean of maximum rating. Within-person correlations at lower triangle and between-person correlations at upper triangle. Confidence interval of correlations in squared brackets. All these ESM measures were calculated only in observations with no missingness.

Table S3.3.1.3

Descriptive Statistics, Within- and Between-person Correlations of Emotion Regulation Strategies in Dataset 1: G(F)ood together (Radboud)

Variable(Index/Measure)	n	M	SDw	SDb	Min	Max	1	2	3	4	5
1. Acceptance	585	5.73	2.62	2.58	2.24	8.68		.51	.18	-.05	.10
								[.32,.65]	[-.04,.39]	[-.26,.17]	[-.12,.31]
2. Reappraisal	585	3.69	2.44	2.55	1.12	7.39	.38		.39	.23	.30
							[.31,.44]		[.19,.56]	[.01,.43]	[.09,.49]
3. Suppression	585	3.4	2.27	2.7	0.82	7.36	.17	.32		.59	.10
							[.09,.25]	[.25,.39]		[.42,.71]	[-.12,.31]
4. Rumination	584	2.39	1.97	2.2	0.49	5.82	-.03	.19	.37		.24
							[-.11,.05]	[.11,.27]	[.30,.44]		[.02,.44]
5. Social Sharing	583	2.23	2.28	2.48	0.35	6.16	.10	.15	.18	.34	
							[.01,.18]	[.07,.23]	[.10,.26]	[.26,.41]	

Note: SDw: Within-person SD. SDb: Between-person SD. Min: mean of minimum rating. Max: mean of maximum rating. Within-person correlations at lower triangle and between-person correlations at upper triangle. Confidence interval of correlations in squared brackets. All these ESM measures were calculated only in observations with no missingness.

Table S3.3.2.1

Descriptive Statistics, Within- and Between-person Correlations of Positive Emotions in Dataset 2: Emotions in daily life (Leuven)

Variable(Index/Measure)	n	M	SDw	SDb	Min	Max	1	2
1. Relaxed	5818	5.78	1.28	2.1	0.99	9.46		.80
								[.72,.86]
2. Happy	5818	5.57	1.5	1.89	1.27	9.17	.55	
							[.53,.57]	

Note: SDw: Within-person SD. SDb: Between-person SD. Min: mean of minimum rating. Max: mean of maximum rating. Within-person correlations at lower triangle and between-person correlations at upper triangle. Confidence interval of correlations in squared brackets. All these ESM measures were calculated only in observations with no missingness.

Table S3.3.2.2

Descriptive Statistics, Within- and Between-person Correlations of Negative Emotions in Dataset 2: Emotions in daily life (Leuven)

**Variable(Index/Measure)**	**n**	**M**	**SDw**	**SDb**	**Min**	**Max**	**1**	**2**	**3**	**4**
1. Angry	5819	1.33	0.96	1.35	0.05	6.37		.65	.64	.68
								[.51,.75]	[.51,.75]	[.55,.77]
2. Anxious	5818	1.24	1.07	1.13	0.06	5.38	.31		.78	.78
							[.29,.34]		[.68,.84]	[.69,.85]
3. Depressed	5818	1.6	1.48	1.26	0.15	5.6	.39	.38		.94
							[.37,.41]	[.36,.40]		[.91,.96]
4. Sad	5817	1.7	1.28	1.46	0.1	6.39	.39	.39	.64	
							[.37,.41]	[.37,.41]	[.63,.66]	

Note: SDw: Within-person SD. SDb: Between-person SD. Min: mean of minimum rating. Max: mean of maximum rating. Within-person correlations at lower triangle and between-person correlations at upper triangle. Confidence interval of correlations in squared brackets. All these ESM measures were calculated only in observations with no missingness.

Table S3.3.2.3

Descriptive Statistics, Within- and Between-person Correlations of Emotion Regulation Strategies in Dataset 2: Emotions in daily life (Leuven)

Variable(Index/Measure)	n	M	SDw	SDb	Min	Max	1	2	3	4	5	6
1. Distraction	5817	2.89	1.66	1.99	0.28	7.92		.51	.42	.41	.30	.62
								[.34,.64]	[.24,.57]	[.23,.56]	[.11,.48]	[.48,.73]
2. Reappraisal	5817	1.76	1.17	1.39	0.16	6.23	.10		.78	.33	.67	.42
							[.08,.13]		[.68,.85]	[.14,.50]	[.54,.77]	[.24,.57]
3. Reflection	5817	2.27	1.25	1.79	0.19	7.48	.06	.30		.41	.64	.33
							[.03,.08]	[.27,.32]		[.23,.56]	[.51,.75]	[.14,.50]
4. Rumination	5817	2.65	1.71	1.91	0.17	7.67	.03	.17	.31		.37	.59
							[.00,.05]	[.15,.20]	[.29,.33]		[.18,.53]	[.45,.71]
5. Social Sharing	5817	2.07	1.23	1.95	0.08	7.69	.05	.24	.29	.14		.21
							[.03,.08]	[.22,.26]	[.26,.31]	[.12,.17]		[.01,.40]
6. Suppression	5819	2.3	1.57	1.75	0.17	7.18	.17	.09	.13	.28	.03	
							[.14,.19]	[.07,.12]	[.10,.15]	[.26,.31]	[.01,.06]	

Note: SDw: Within-person SD. SDb: Between-person SD. Min: mean of minimum rating. Max: mean of maximum rating. Within-person correlations at lower triangle and between-person correlations at upper triangle. Confidence interval of correlations in squared brackets. All these ESM measures were calculated only in observations with no missingness.

Table S3.3.3.1

Descriptive Statistics, Within- and Between-person Correlations of Positive Emotions in Dataset 3: 3-wave longitudinal study (Leuven)

**Variable(Index/Measure)**	**n**	**M**	**SDw**	**SDb**	**Min**	**Max**	**1**	**2**	**3**
1. Relaxed	12346	6	1.08	2.12	0.92	9.54		.81	.64
								[.75,.85]	[.55,.72]
2. Happy	12346	5.87	1.12	1.9	1.24	9.48	.49		.78
							[.48,.51]		[.72,.83]
3. Cheerful	12346	5.2	1.11	2.06	0.74	9.24	.36	.58	
							[.35,.38]	[.57,.59]	

Note:

SDw: Within-person SD. SDb: Between-person SD. Min: mean of minimum rating. Max: mean of maximum rating. Within-person correlations at lower triangle and between-person correlations at upper triangle. Confidence interval of correlations in squared brackets. All these ESM measures were calculated only in observations with no missingness.

Table S3.3.3.2

Descriptive Statistics, Within- and Between-person Correlations of Negative Emotions in Dataset 3: 3-wave longitudinal study (Leuven)

Variable(Index/Measure)	n	M	SDw	SDb	Min	Max	1	2	3	4	5	6
1. Angry	12346	1.19	0.82	1.28	0.09	6.41		.79	.60	.83	.84	.58
								[.73,.84]	[.50,.68]	[.79,.87]	[.80,.88]	[.47,.66]
2. Depressed	12346	1.26	0.98	1.2	0.09	5.7	.41		.75	.85	.90	.63
							[.40,.43]		[.68,.81]	[.81,.89]	[.86,.92]	[.53,.70]
3. Lonely	12346	1.78	1.33	1.65	0.11	6.93	.21	.35		.70	.72	.53
							[.20,.23]	[.34,.37]		[.62,.76]	[.65,.78]	[.42,.62]
4. Anxious	12346	1.03	0.81	0.99	0.08	5.28	.30	.38	.22		.83	.63
							[.29,.32]	[.37,.40]	[.21,.24]		[.78,.87]	[.54,.71]
5. Sad	12346	1.31	0.94	1.3	0.09	6.29	.42	.59	.37	.39		.61
							[.41,.44]	[.57,.60]	[.36,.39]	[.37,.40]		[.52,.69]
6. Stressed	12346	2.32	1.17	1.96	0.13	7.84	.31	.31	.18	.31	.28	
							[.29,.32]	[.29,.33]	[.17,.20]	[.30,.33]	[.26,.29]	

Note: SDw: Within-person SD. SDb: Between-person SD. Min: mean of minimum rating. Max: mean of maximum rating. Within-person correlations at lower triangle and between-person correlations at upper triangle. Confidence interval of correlations in squared brackets. All these ESM measures were calculated only in observations with no missingness.

Table S3.3.3.3

Descriptive Statistics, Within- and Between-person Correlations of Emotion Regulation Strategies in Dataset 3: 3-wave longitudinal study (Leuven)

Variable(Index/Measure)	n	M	SDw	SDb	Min	Max	1	2	3	4	5	6
1. Distraction	12346	2.43	1.87	1.8	0.19	7.38		.49	.35	.42	.46	.80
								[.38,.59]	[.22,.47]	[.30,.53]	[.34,.56]	[.74,.84]
2. Reappraisal	12346	1.51	1.08	1.29	0.15	6.01	.13		.71	.60	.49	.45
							[.12,.15]		[.63,.77]	[.50,.68]	[.38,.59]	[.33,.55]
3. Social Sharing	12346	1.8	1.09	1.72	0.11	7.37	.04	.25		.48	.39	.29
							[.03,.06]	[.23,.27]		[.36,.58]	[.26,.50]	[.16,.42]
4. Rumination	12346	1.83	1.35	1.61	0.14	7.01	.13	.16	.14		.68	.38
							[.11,.14]	[.14,.17]	[.12,.16]		[.60,.75]	[.25,.49]
5. Worry	12346	3.11	1.7	2.2	0.21	8.17	.12	.14	.13	.25		.50
							[.10,.13]	[.12,.16]	[.11,.14]	[.24,.27]		[.38,.59]
6. Suppression	12346	2.01	1.73	1.56	0.19	6.96	.30	.11	.01	.18	.19	
							[.29,.32]	[.09,.13]	[-.01,.02]	[.16,.20]	[.17,.20]	

Note: SDw: Within-person SD. SDb: Between-person SD. Min: mean of minimum rating. Max: mean of maximum rating. Within-person correlations at lower triangle and between-person correlations at upper triangle. Confidence interval of correlations in squared brackets. All these ESM measures were calculated only in observations with no missingness.

Table S3.3.4.1

Descriptive Statistics, Within- and Between-person Correlations of Positive Emotions in Dataset 4: Emotion regulation in daily life (Tilburg)

Variable(Index/Measure)	n	M	SDw	SDb	Min	Max	1	2	3	4	5	6	7
1. Energetic	7929	4.09	1.34	1.96	0.83	8.04		.65	.79	.66	.36	.77	.58
								[.56,.73]	[.72,.84]	[.56,.73]	[.22,.48]	[.71,.83]	[.47,.67]
2. Content	7934	5.3	1.3	1.91	1.34	8.66	.43		.75	.49	.53	.84	.59
							[.41,.45]		[.67,.81]	[.37,.59]	[.42,.63]	[.79,.88]	[.48,.68]
3. Enthusiastic	7944	4.31	1.5	1.91	0.95	8.07	.50	.53		.64	.26	.79	.65
							[.49,.52]	[.51,.54]		[.54,.72]	[.11,.39]	[.72,.84]	[.56,.73]
4. Deteremined	7922	3.6	1.46	1.91	0.72	7.72	.42	.31	.33		.28	.59	.63
							[.40,.44]	[.29,.33]	[.31,.35]		[.14,.41]	[.48,.67]	[.53,.71]
5. Calm	7929	5.71	1.46	1.93	1.32	8.77	.06	.29	.09	.09		.46	.28
							[.04,.08]	[.27,.31]	[.07,.11]	[.07,.11]		[.34,.57]	[.14,.41]
6. Joyful	7919	5.17	1.37	1.85	1.36	8.49	.51	.60	.57	.35	.21		.59
							[.49,.52]	[.58,.61]	[.55,.58]	[.33,.37]	[.19,.23]		[.49,.68]
7. Grateful	7904	3.89	1.87	1.84	1	7.99	.33	.44	.39	.30	.16	.45	
							[.31,.35]	[.42,.46]	[.37,.41]	[.27,.32]	[.14,.18]	[.43,.47]	

Note: SDw: Within-person SD. SDb: Between-person SD. Min: mean of minimum rating. Max: mean of maximum rating. Within-person correlations at lower triangle and between-person correlations at upper triangle. Confidence interval of correlations in squared brackets. All these ESM measures were calculated only in observations with no missingness.

Table S3.3.4.2

Descriptive Statistics, Within- and Between-person Correlations of Negative Emotions in Dataset 4: Emotion regulation in daily life (Tilburg)

Variable(Index/Measure)	n	M	SDw	SDb	Min	Max	1	2	3	4	5	6
1. Irritated	7939	1.68	1.02	1.66	0.24	6.88		.59	.74	.62	.83	.61
								[.49,.68]	[.67,.80]	[.53,.71]	[.77,.87]	[.50,.69]
2. Bored	7923	2.24	1.06	1.85	0.28	6.98	.16		.46	.39	.55	.36
							[.13,.18]		[.34,.57]	[.26,.51]	[.44,.65]	[.23,.48]
3. Nervous	7909	1.46	1.03	1.34	0.24	5.83	.23	.11		.79	.85	.75
							[.21,.25]	[.09,.14]		[.73,.84]	[.80,.89]	[.67,.80]
4. Sad	7898	1.24	1.21	1.07	0.25	4.9	.31	.14	.29		.84	.91
							[.29,.33]	[.12,.17]	[.27,.31]		[.78,.88]	[.88,.93]
5. Angry	7886	1.1	0.93	1.11	0.21	5.14	.51	.09	.25	.50		.77
							[.49,.52]	[.07,.11]	[.23,.28]	[.49,.52]		[.70,.82]
6. Depressed	7884	1.53	1.31	1.36	0.28	5.74	.34	.21	.27	.62	.45	
							[.32,.36]	[.19,.23]	[.25,.29]	[.61,.63]	[.43,.47]	

Note: SDw: Within-person SD. SDb: Between-person SD. Min: mean of minimum rating. Max: mean of maximum rating. Within-person correlations at lower triangle and between-person correlations at upper triangle. Confidence interval of correlations in squared brackets. All these ESM measures were calculated only in observations with no missingness.

Table S3.3.4.3

Descriptive Statistics, Within- and Between-person Correlations of Emotion Regulation Strategies in Dataset 4: Emotion regulation in daily life (Tilburg)

Variable(Index/Measure)	n	M	SDw	SDb	Min	Max	1	2	3	4	5	6	7
1. Distraction	7869	3.14	1.81	2	0.45	7.48		.76	.56	.56	.20	.38	.42
								[.70, .82]	[.45, .66]	[.45, .66]	[.06, .34]	[.25, .50]	[.29, .53]
2. Avoidance	7862	2.41	1.59	1.68	0.41	6.74	.30		.57	.43	.11	.32	.44
							[.28, .32]		[.46, .66]	[.30, .54]	[-.03, .26]	[.18, .45]	[.32, .55]
3. Rumination	7851	2.1	1.37	1.72	0.33	6.7	.11	.13		.66	.14	.70	.71
							[.09, .14]	[.11, .15]		[.56, .73]	[.00, .28]	[.62, .77]	[.63, .78]
4. Problem Solving	7850	2.01	1.34	1.67	0.34	6.58	.15	.14	.27		.30	.70	.62
							[.13, .17]	[.12, .16]	[.25, .29]		[.16, .43]	[.62, .77]	[.52, .70]
5. Acceptance	7850	3.64	2.16	1.87	0.74	7.6	.03	.00	-.04	.05		.23	.17
							[.00, .05]	[-.02, .03]	[-.06,-.01]	[.02, .07]		[.08, .36]	[.02, .31]
6. Social Sharing	7831	1.71	1.27	1.63	0.24	6.44	.13	.07	.30	.28	.05		.84
							[.11, .15]	[.05, .09]	[.28, .32]	[.26, .30]	[.03, .07]		[.79, .88]
7. Co-Brooding	7815	1.25	1.06	1.21	0.19	5.24	.07	.07	.34	.22	.03	.56	
							[.05, .10]	[.05, .09]	[.32, .36]	[.20, .24]	[.01, .06]	[.54, .57]	

Note: SDw: Within-person SD. SDb: Between-person SD. Min: mean of minimum rating. Max: mean of maximum rating. Within-person correlations at lower triangle and between-person correlations at upper triangle. Confidence interval of correlations in squared brackets. All these ESM measures were calculated only in observations with no missingness.

Table S3.3.5.1

Descriptive Statistics, Within- and Between-person Correlations of Positive Emotions in Dataset 5: Outside-in (Ghent)

**Variable(Index/Measure)**	**n**	**M**	**SDw**	**SDb**	**Min**	**Max**	**1**	**2**	**3**
1. Happy	9838	7.46	2.07	1.9	2.19	9.53		.80	.63
								[.75,.85]	[.55,.71]
2. Relaxed	9837	6.88	2.3	2.26	1.44	9.53	.38		.68
							[.37,.40]		[.60,.74]
3. Energetic	9838	5.39	2.72	2.54	0.82	9.27	.40	.23	
							[.38,.42]	[.21,.25]	

Note:

SDw: Within-person SD. SDb: Between-person SD. Min: mean of minimum rating. Max: mean of maximum rating. Within-person correlations at lower triangle and between-person correlations at upper triangle. Confidence interval of correlations in squared brackets. All these ESM measures were calculated only in observations with no missingness.

Table S3.3.5.2

Descriptive Statistics, Within- and Between-person Correlations of Negative Emotions in Dataset 5: Outside-in (Ghent)

Variable(Index/Measure)	n	M	SDw	SDb	Min	Max	1	2	3	4	5	6
1. Angry	9838	0.98	1.35	1.55	0.07	6.66		.64	.87	.88	.72	.72
								[.55,.71]	[.84,.90]	[.85,.91]	[.65,.78]	[.65,.78]
2. Annoyed	9838	1.88	2.11	1.85	0.08	6.83	.21		.61	.59	.70	.74
							[.19,.23]		[.52,.69]	[.49,.67]	[.63,.77]	[.67,.79]
3. Anxious	9836	0.92	1.45	1.25	0.08	5.04	.31	.17		.83	.77	.72
							[.30,.33]	[.15,.19]		[.79,.87]	[.70,.82]	[.65,.78]
4. Sad	9838	1.16	1.43	1.69	0.06	6.83	.44	.18	.31		.71	.73
							[.43,.46]	[.16,.20]	[.30,.33]		[.63,.77]	[.66,.79]
5. Stressed	9838	2.04	2.16	2.03	0.19	7.6	.23	.22	.31	.23		.86
							[.21,.25]	[.20,.24]	[.29,.33]	[.21,.24]		[.82,.89]
6. Uncertain	9838	1.55	2.01	1.56	0.11	6.08	.27	.26	.42	.29	.39	
							[.25,.29]	[.24,.28]	[.41,.44]	[.27,.30]	[.38,.41]	

Note: SDw: Within-person SD. SDb: Between-person SD. Min: mean of minimum rating. Max: mean of maximum rating. Within-person correlations at lower triangle and between-person correlations at upper triangle. Confidence interval of correlations in squared brackets. All these ESM measures were calculated only in observations with no missingness.

Table S3.3.5.3

Descriptive Statistics, Within- and Between-person Correlations of Emotion Regulation Strategies in Dataset 5: Outside-in (Ghent)

Variable(Index/Measure)	n	M	SDw	SDb	Min	Max	1	2	3	4	5	6	7	8
1. Reappraisal	9838	2.18	2.37	1.79	0.12	6.63		.91	.89	.66	.73	.92	.92	.83
								[.89,.93]	[.85,.91]	[.58,.73]	[.66,.79]	[.90,.94]	[.90,.94]	[.78,.87]
2. Distraction	9838	2.36	2.58	1.81	0.17	6.82	.34		.90	.69	.75	.91	.90	.83
							[.32,.36]		[.87,.92]	[.62,.76]	[.69,.81]	[.89,.93]	[.87,.92]	[.79,.87]
3. Social Support	9838	2.24	2.47	1.87	0.16	6.67	.25	.22		.58	.76	.93	.93	.92
							[.23,.27]	[.21,.24]		[.49,.67]	[.70,.81]	[.91,.95]	[.91,.94]	[.89,.94]
4. Suppression	9838	2.46	2.58	1.92	0.24	7.02	.19	.26	.05		.82	.63	.61	.55
							[.17,.21]	[.24,.28]	[.03,.07]		[.77,.86]	[.54,.70]	[.52,.69]	[.45,.64]
5. Rumination	9837	2.52	2.54	1.96	0.26	7.09	.20	.22	.27	.31		.73	.73	.72
							[.18,.22]	[.20,.24]	[.26,.29]	[.30,.33]		[.66,.79]	[.66,.78]	[.65,.78]
6. Self-compassion (Support)	9837	2.39	2.62	1.71	0.22	6.6	.30	.31	.25	.19	.20		.98	.89
							[.29,.32]	[.29,.32]	[.23,.27]	[.17,.21]	[.18,.22]		[.97,.98]	[.86,.91]
7. Self-compssion (Cheer-up)	9838	2.46	2.67	1.72	0.22	6.64	.32	.30	.27	.16	.25	.47		.90
							[.30,.33]	[.28,.32]	[.25,.29]	[.14,.18]	[.24,.27]	[.45,.48]		[.87,.92]
8. Expression	9838	2.18	2.41	1.75	0.24	6.43	.19	.16	.35	.05	.23	.27	.29	
							[.17,.21]	[.14,.18]	[.33,.37]	[.03,.07]	[.21,.24]	[.25,.29]	[.27,.30]	

Note: SDw: Within-person SD. SDb: Between-person SD. Min: mean of minimum rating. Max: mean of maximum rating. Within-person correlations at lower triangle and between-person correlations at upper triangle. Confidence interval of correlations in squared brackets. All these ESM measures were calculated only in observations with no missingness.

Supplemental Materials 4 – Multilevel Confirmatory Factor Analysis per Dataset

We ran Multilevel Confirmatory Factor Analyses (MCFA; see procedures in Eisele et al., 2021) to confirm the factor structure for positive emotions and negative emotions at both within-adolescent and between-adolescent levels. In the MCFA, positive emotion items were loaded on an overall positive emotion factor, negative emotion items were loaded on an overall negative emotion factor. The positive and negative emotion latent factors were allowed to correlate. We inspected model fit with conventional cutoff values (RMSEA $< .08$, CFI $> .90$ and TLI $> .90$; see Schermelleh-Engel et al., 2003). When model fits were unsatisfactory, as in datasets 3, 4, and 5, we allowed residual variance of overlapping items to correlate to improve model fit. In general, model fit at the within-person level was usually worse than at the between-person level. While the TLI is not acceptable in some models, both the RMSEA and CFI are. Overall, positive and negative emotions loaded separately on two factors as indicated with satisfactory fit indices, as shown in Table S3. In other words, it was suitable to take the mean of the positive emotions as a single-factor index, and likewise for negative emotions.

Table S4

Multilevel Confirmatory Factor Analysis per Datasets

Dataset	Within-person					Between-person				
	**SFL**	**X2**	**RMSEA**	**CFI**	**TLI**	**SFL**	**X2**	**RMSEA**	**CFI**	**TLI**
G(F)ood together (Radboud)	.43–.77	359.27	.06	.95	.86	.57–.98	74.06	.02	.99	.98
Emotions in daily life 2011 (Leuven)	.50–.84	231.03	.07	.98	.91	.70–.98	24.69	.02	$> .99$	.99
3-wave longitudinal study (Leuven)*	.43–.85	1,025.20	.06	.97	.91	.68–.99	104.47	.02	$> .99$	.99
Emotions in daily life (Tilburg)*	.26–.80	3,011.13	.08	.90	.76	.44–.97	408.03	.03	.99	.97
Outside-in (Ghent)*	.38–.76	876.50	.06	.95	.84	.72–.94	235.35	.03	.99	.96

Note: SFL = standardized factor loadings (all $p < .001$). X2 = Chi-square. RMSEA = Root Mean Square Error of Approximation. CFI = Comparative Fit Index. TLI = Tucker Lewis Index. When evaluating the fit of the within-person model, a saturated between-person model was specified. When evaluating the between-person model, a saturated within-person was specified. * For datasets 3, 4 and 5, we included correlations between residual variances of overlapping items (e.g., relaxed with stressed) to improve model fit. For the within-person model for dataset 3, we included the correlation between the items ”relaxed” and ”stressed” at the within-person level. For the within-person model for dataset 4, we included the correlation between the items ”angry” and ”irritated” and ”sad” and ”low” at the within-person level. For the within-person model for dataset 5, we included the correlation between the items ”angry” and ”sad” at the within-person level.

Supplemental Materials 5: Specification of Within-Person Mediation Model and Full Results of all Multilevel Models

Detailed Specifications of the Within-Person Mediation Model 1M

In Model 1M, we examined both the direct and indirect paths. The direct paths included the a-path (from lagged emotion differentiation to emotion regulation variability), the b-path (from emotion regulation variability to emotion intensity), and the c'-path (from lagged emotion differentiation to emotion intensity). The a-path corresponds to the effect analyzed in Model 1A, while the b-path and c'-path reflect how emotion intensity changes directly in response to fluctuations in emotion differentiation and emotion regulation variability. The indirect path in Model 1M, representing the mediation effect, is calculated as the sum of two components: the product of the a-path and b-path, and the covariance between a-path and b-path.

Estimation of the Direct Paths in Model 1M

The direct paths of Model 1M were estimated using the *nlme* package. Model 1M employed a stacked dataset, in which each row of data was split into two rows: one emphasizing the outcome (emotion intensity) and the other the mediator (emotion regulation variability) (Bauer et al., 2006; Bolger & Laurenceau, 2013). In this setup, emotion regulation variability serves as both an outcome in the a-path and a predictor in the b-path. Since we focused on within-person fluctuations, emotion regulation variability had to be modeled as a within-person component, precluding the modeling of its between-person component as an outcome. Consequently, the predictor (emotion differentiation) and outcome (emotion intensity) also had to be specified as within-person components, excluding their between-person components. To align with this approach, the intercepts of the mediator and outcome were fixed to 0 because the within-person components, being person-mean-centered, had zero within-person means. Although this approach allowed us to evaluate within-person mediation accurately (Bolger & Laurenceau,

2013), it prevented us from simultaneously estimating between-person effects within Model 1M, unlike what we did in other models (e.g., Model 1A). However, the three between-person relations among emotion differentiation, emotion regulation variability, and emotion intensity were already evaluated in Models 1A, 1B, 1C, 2A, and 2B.

Model 1M with positive emotion specification encountered an evaluation error for the first-order autocorrelation term on the residual. To check whether estimates deviated when the residual autocorrelation term was removed, we undertook the following steps. First, we ran Model 1M (positive emotion) in a two-step manner, including the autocorrelation term. This involved running Model 1A (positive emotion) and a modified version of Model 2A (positive emotion) with the outcome variable replaced by positive emotion intensity and the covariate positive emotion intensity replaced by lagged positive emotion intensity. Then, we ran these two models (two-step model 1M) again, this time excluding the autocorrelation term on the residual. Finally, we compared the fixed effects of interest (e.g., a-path: positive emotion differentiation → emotion regulation variability, and b-path: emotion regulation variability → positive emotion intensity) between the two-step models with and without the autocorrelation term. The comparisons revealed that the fixed effects for the a-, b-, and c'-paths remained in the same direction and statistical significance. Based on these findings, we proceeded with Model 1M (positive emotion) using the one-step approach described in the main text (i.e., evaluating the a-, b-, and c'-paths in a single multilevel model after the stacking procedure) without including the first-order autocorrelation term on the residual, which allowed Model 1M (positive emotion) to converge. As a result, in this and the subsequent Supplemental Materials, we report the Model 1M (positive emotion) results evaluated without specifying the first-order autocorrelation term on the residual.

All within-person mediation models (whether for positive or negative emotions and across varied specifications in sensitivity analyses) produced warnings about singularity precision, indicating that some random slopes were estimated as zero or that correlations

between them were approaching 1 or -1 (Bates, Kliegl, et al., 2015). To address this, we first simplified the models by removing random effects for variables not central to our primary interests (e.g., from lagged emotion intensity to emotion intensity). However, the singularity warnings persisted. Upon inspecting the outputs from both the full models and the simplified models without additional random slopes, we found that the warnings were caused by correlations between dataset-level random effects approaching 1 or -1. At the person-level, however, none of the random effects were estimated as zero, nor were any correlations between them near 1 or -1. Given that our primary focus is on interpreting person-level results, we deemed it acceptable to proceed with the estimates despite the presence of singularity precision warnings.

Estimation of the Indirect Path of Model 1M

Making use of the estimates from *nlme*, we can calculate the indirect path as the sum of two components: product of the a-path and b-path, and covariance of person-level random effects of the a-path and b-path. To further obtain the confidence interval of the indirect path, we made use of the Monte Carlo script by Preacher and Selig (2010). To prepare for this, we used the *lme4* package (Bates, Mächler, et al., 2015) in addition to *nlme* which we have used for evaluating other models. Both the *nlme* and *lme4* packages can evaluate three-level models, grouping measurements within adolescents and adolescents within datasets. Apart from *nlme* and *lme4*, other software options exist, namely the *brms* package (see Ram, 2022 for tutorial) and Mplus, a proprietary software (see McNeish & MacKinnon, 2022 for tutorial). However, unlike *nlme* and *lme4*, *brms* and Mplus can not yet simultaneously handle three-level nested structure and estimation of within-person mediation. So, we could only proceed with the *nlme* and *lme4* packages.

For models other than Model 1M, we primarily used the *nlme* package, as it supports the inclusion of a first-order autocorrelation term on the residual, which *lme4* does not. However, only *lme4* has compatible resources for extracting the asymptotic

covariance of random effects for two paths, an estimate needed for accurately assessing the confidence interval of the mediation effect (see Ram (2017) for an overview). This estimate affects the dispersion of the Monte Carlo resampled indirect effect: the larger it is, the wider the resample distribution's bell curve.

It is still possible to produce a confidence interval without this estimate using *nlme* results, but the interval will be liberal. This means that even if the interval does not include zero, we cannot be certain this would remain the case if the asymptotic covariance of random effects were included. Conversely, if the interval does include zero, we can be confident it will continue to include zero even when the missing estimate is added.

In summary, each package has distinct advantages and disadvantages. The *nlme* results come from better-specified models but provide a liberal confidence interval, which is not reliable for rejecting the null hypothesis of no within-person mediation. Conversely, *lme4* can produce a confidence interval capable of rejecting this null hypothesis, but it does so based on model estimates evaluated without the first-order autocorrelation residual term. For this exploratory research question, we computed the 95% confidence intervals for mediation using the Monte Carlo script by Preacher and Selig (2010) with results from both packages. To ensure robustness of our results, we reported the more conservative results between the two sets. For example, if one set gave an interval that crossed zero but the other set did not, we reported the set that crossed zero.

Before using *lme4* results, it was necessary to assess whether *lme4* results were similar as those from *nlme*. Therefore, we compared the fixed effects of interest (e.g., a-path: emotion differentiation → emotion regulation variability, and b-path: emotion regulation variability → emotion intensity) in Model 1M as estimated by both packages. The fixed effects showed the same direction and statistical significance in both *nlme* and *lme4*.

We initially planned to use the *lme4* package to reevaluate models under various

specifications detailed in Supplemental Materials 6 and 7. However, under those sensitivity analysis specifications, the *lme4* estimates showed very large deviations from those of *nlme* and encountered other errors (see Supplemental Materials 6 and 7 for details), making it impractical to conduct sensitivity analyses as extensively as we did for the direct paths in Model 1M and for estimates in other models without within-person mediation.

Full Multilevel Model Results

Table S5

Fixed Effect Estimates of Within-Person Temporal Associations and Between-Person Differences in Between Emotion Differentiation, Emotion Regulation Variability, and Emotion Intensity

	Negative Emotions *b* [95% *CI]*	Positive Emotions *b* [95% *CI]*
Outcome: Emotion regulation variability (Model 1A)	N = 752, n = 25867	N = 751, n = 25851
Within-person (time-varying)		
Lagged emotion differentiation	**-0.009 [-0.014, -0.005]**	**-0.009 [-0.014, -0.004]**
Lagged emotion intensity	-0.018 [-0.043, 0.007]	-0.005 [-0.017, 0.007]
Emotion regulation intensity	0.295 [-0.283, 0.872]	0.280 [-0.276, 0.837]
Time trend	**-0.003 [-0.004, -0.003]**	**-0.003 [-0.004, -0.002]**
Between-person (time-invariant)		
Intercept	**3.895 [2.773, 5.018]**	**4.056 [2.819, 5.294]**
Emotion differentiation	0.068 [-0.072, 0.207]	-0.053 [-0.258, 0.153]
Emotion intensity	-0.023 [-0.128, 0.083]	**-0.107 [-0.181, -0.034]**
Emotion regulation intensity	**-0.552 [-0.629, -0.475]**	**-0.561 [-0.631, -0.492]**
Age	-0.005 [-0.063, 0.054]	-0.012 [-0.077, 0.053]
Gender (female = 1, male = 0)	**0.412 [0.188, 0.637]**	**0.347 [0.120, 0.575]**
Outcome: Strategy switching (Model 1B)	N = 752, n = 25867	N = 751, n = 25851
Within-person (time-varying)		
Endorsement change	**-0.436 [-0.576, -0.296]**	**-0.437 [-0.575, -0.300]**
Lagged emotion differentiation	**-0.004 [-0.007, -0.002]**	**-0.004 [-0.007, 0.000]**
Lagged emotion intensity	-0.010 [-0.025, 0.005]	-0.002 [-0.013, 0.009]
Emotion regulation intensity	**-0.102 [-0.153, -0.051]**	**-0.102 [-0.149, -0.055]**
Time trend	**-0.002 [-0.002, -0.001]**	**-0.002 [-0.002, -0.001]**

Table S5

Fixed Effect Estimates of Within-Person Temporal Associations and Between-Person Differences in Between Emotion Differentiation, Emotion Regulation Variability, and Emotion Intensity (continued)

	Negative Emotions *b* [95% *CI]*	Positive Emotions *b* [95% *CI]*
Between-person (time-invariant)		
Intercept	**0.978 [0.346, 1.610]**	**0.993 [0.317, 1.670]**
Endorsement change	0.017 [-0.027, 0.061]	0.008 [-0.036, 0.052]
Emotion differentiation	**0.156 [0.086, 0.226]**	0.017 [-0.089, 0.123]
Emotion intensity	0.032 [-0.022, 0.085]	-0.035 [-0.073, 0.002]
Emotion regulation intensity	0.015 [-0.029, 0.058]	0.011 [-0.029, 0.052]
Age	**0.032 [0.002, 0.061]**	0.031 [-0.001, 0.064]
Gender (female = 1, male = 0)	**0.138 [0.026, 0.250]**	**0.127 [0.012, 0.242]**
Outcome: Endorsement change (Model 1C)	N = 752, n = 25867	N = 751, n = 25851
Within-person (time-varying)		
Strategy switching	0.312 [-1.140, 1.764]	0.302 [-1.135, 1.740]
Lagged emotion differentiation	**-0.008 [-0.012, -0.004]**	**-0.007 [-0.012, -0.003]**
Lagged emotion intensity	**-0.017 [-0.034, 0.000]**	-0.004 [-0.012, 0.004]
Emotion regulation intensity	0.054 [-0.233, 0.341]	0.058 [-0.228, 0.344]
Time trend	**-0.002 [-0.003, -0.002]**	**-0.002 [-0.003, -0.001]**
Between-person (time-invariant)		
Intercept	**2.427 [1.550, 3.304]**	**2.523 [1.653, 3.392]**
Strategy switching	**-0.234 [-0.318, -0.150]**	**-0.238 [-0.322, -0.154]**
Emotion differentiation	-0.082 [-0.184, 0.019]	**-0.148 [-0.296, 0.000]**
Emotion intensity	-0.072 [-0.148, 0.004]	0.025 [-0.028, 0.079]
Emotion regulation intensity	**-0.677 [-0.733, -0.621]**	**-0.696 [-0.746, -0.645]**
Age	-0.009 [-0.056, 0.039]	-0.014 [-0.061, 0.033]
Gender (female = 1, male = 0)	**0.215 [0.054, 0.376]**	**0.203 [0.041, 0.366]**
Within-person mediation (Model 1M)	N = 756, n = 52003	N = 755, n = 51991
Within-person (time-varying)		
Lagged emotion differentiation → emotion regulation variability (a-path)	**-0.013 [-0.018, -0.008]**	**-0.014 [-0.020, -0.008]**
Lagged emotion intensity → emotion regulation variability	-0.029 [-0.085, 0.026]	-0.005 [-0.025, 0.016]
Time trend → emotion regulation variability	**-0.005 [-0.006, -0.004]**	**-0.005 [-0.005, -0.004]**
Emotion regulation variability → emotion intensity (b-path)	**0.073 [0.038, 0.108]**	**-0.049 [-0.091, -0.006]**

Table S5

Fixed Effect Estimates of Within-Person Temporal Associations and Between-Person Differences in Between Emotion Differentiation, Emotion Regulation Variability, and Emotion Intensity (continued)

	Negative Emotions *b* [95% *CI]*	Positive Emotions *b* [95% *CI]*
Emotion regulation intensity → emotion intensity	**0.234 [0.166, 0.303]**	**-0.102 [-0.187, -0.018]**
Lagged emotion intensity → emotion intensity	**0.259 [0.204, 0.315]**	**0.300 [0.249, 0.351]**
Lagged emotion differentiation → emotion intensity (c'-path)	**0.008 [0.003, 0.013]**	**-0.016 [-0.026, -0.006]**
Time trend → emotion intensity	**-0.002 [-0.002, -0.001]**	**-0.002 [-0.003, -0.001]**
Mediation (sum of covariance and product of a- and b-path)	-0.000 [-0.001, 0.000]	-0.000 [-0.001, 0.001]
Within-person mediation (Model 1M) with person-level emotion differentiation as a moderator to a-path and b-path	N = 756, n = 52003	N = 755, n = 51991
Within-person (time-varying)		
Lagged emotion differentiation → emotion regulation variability (a-path)	**-0.015 [-0.020, -0.010]**	**-0.016 [-0.022, -0.010]**
Lagged emotion intensity → emotion regulation variability	-0.031 [-0.088, 0.026]	-0.005 [-0.026, 0.016]
Time trend → emotion regulation variability	**-0.005 [-0.006, -0.004]**	**-0.005 [-0.005, -0.004]**
Emotion regulation variability → emotion intensity (b-path)	**0.074 [0.038, 0.110]**	**-0.048 [-0.090, -0.006]**
Emotion regulation intensity → emotion intensity	**0.236 [0.167, 0.306]**	**-0.103 [-0.188, -0.018]**
Lagged emotion intensity → emotion intensity	**0.259 [0.201, 0.317]**	**0.301 [0.249, 0.352]**
Lagged emotion differentiation → emotion intensity (c'-path)	**0.008 [0.003, 0.014]**	**-0.016 [-0.026, -0.006]**
Time trend → emotion intensity	**-0.002 [-0.002, -0.001]**	**-0.002 [-0.003, -0.001]**
Lagged emotion differentiation → emotion regulation variability (a-path), moderated by between-person emotion differentiation	**-0.006 [-0.010, -0.002]**	-0.010 [-0.020, 0.000]
Emotion regulation variability → emotion intensity (b-path), moderated by between-person emotion differentiation	**-0.034 [-0.057, -0.010]**	0.046 [-0.002, 0.094]
Outcome: Emotion differentiation (Model 2A)	N = 751, n = 25830	N = 750, n = 25834
Within-person (time-varying)		
Emotion regulation variability	**-0.514 [-0.731, -0.296]**	**-0.276 [-0.496, -0.057]**
Lagged emotion differentiation	**-0.020 [-0.032, -0.007]**	**0.031 [0.001, 0.062]**
Emotion intensity	**-3.884 [-4.989, -2.779]**	**0.519 [0.206, 0.832]**
Emotion regulation intensity	-0.026 [-0.110, 0.058]	**-0.150 [-0.246, -0.055]**
Time trend	**-0.006 [-0.008, -0.004]**	**0.004 [0.003, 0.006]**
Between-person (time-invariant)		
Intercept	**-1.225 [-1.874, -0.576]**	-0.547 [-1.221, 0.127]

Table S5

Fixed Effect Estimates of Within-Person Temporal Associations and Between-Person Differences in Between Emotion Differentiation, Emotion Regulation Variability, and Emotion Intensity (continued)

	Negative Emotions *b* [95% *CI]*	Positive Emotions *b* [95% *CI]*
Emotion regulation variability	-0.035 [-0.072, 0.001]	-0.012 [-0.039, 0.015]
Emotion intensity	**-0.238 [-0.296, -0.180]**	**0.035 [0.005, 0.065]**
Emotion regulation intensity	-0.043 [-0.087, 0.001]	-0.014 [-0.044, 0.015]
Age	**-0.046 [-0.081, -0.011]**	**-0.069 [-0.100, -0.037]**
Gender (female = 1, male = 0)	0.047 [-0.074, 0.168]	**-0.149 [-0.239, -0.058]**
Outcome: Emotion differentiation (Model 2B)	N = 751, n = 25830	N = 750, n = 25834
Within-person (time-varying)		
Strategy switching	**-0.432 [-0.730, -0.133]**	**-0.306 [-0.525, -0.086]**
Endorsement change	**-0.550 [-0.771, -0.328]**	**-0.262 [-0.480, -0.043]**
Lagged emotion differentiation	**-0.018 [-0.030, -0.006]**	**0.031 [0.000, 0.062]**
Emotion intensity	**-3.887 [-5.009, -2.764]**	**0.519 [0.205, 0.833]**
Emotion regulation intensity	-0.035 [-0.121, 0.051]	**-0.149 [-0.243, -0.054]**
Time trend	**-0.006 [-0.008, -0.004]**	**0.004 [0.003, 0.006]**
Between-person (time-invariant)		
Intercept	**-1.264 [-1.921, -0.606]**	-0.558 [-1.234, 0.119]
Strategy switching	0.055 [-0.008, 0.118]	-0.004 [-0.052, 0.044]
Endorsement change	**-0.091 [-0.140, -0.042]**	-0.018 [-0.055, 0.019]
Emotion intensity	**-0.239 [-0.297, -0.181]**	**0.034 [0.004, 0.064]**
Emotion regulation intensity	**-0.068 [-0.114, -0.022]**	-0.017 [-0.049, 0.015]
Age	**-0.044 [-0.079, -0.009]**	**-0.068 [-0.099, -0.037]**
Gender (female = 1, male = 0)	0.034 [-0.086, 0.153]	**-0.148 [-0.238, -0.057]**

Note: Significant effects are displayed in bold. n: number of ESM assessments; N: number of adolescents; b: unstandardized effect; CI: confidence interval. In Model 1M, n is doubled because of how data have undergone the stacking preparation step.

Supplemental Materials 6: Sensitivity analyses using the successive approach to calculate Bray-Curtis dissimilarity

In the main analyses, we calculated emotion regulation variability as Bray-Curtis dissimilarity by comparing the moment of interest with all other moments the same individual reported, which is known as the all-moment comparison approach. An alternative approach to calculating Bray-Curtis dissimilarity is by the successive temporal comparison which compares the moment of interest with the previous moment. This approach of calculation is not available if such previous moments have missingness, but the all-moment comparison approach can still compute the dissimilarity as long as there are at least two observations. As sensitivity analyses, we ran the same analyses with the successive temporal comparison approach. As shown in Table S6, the momentary reciprocal hinderance between negative emotion differentiation and emotion regulation variability was also seen when emotion regulation variability was calculated in the successive temporal comparison approach. In terms of individual differences, similar to our main findings, there were no significant associations between negative emotion differentiation and emotion regulation variability (model 2A). In summary, our confirmatory hypotheses about the relations between negative emotion differentiation and emotion regulation variability were robust.

As for the sensitivity analyses of exploratory models on two emotion regulation variability subcomponents, model 1B, 1C, and 2B showed similar findings that there were momentary reciprocal hinderance between negative emotion differentiation and emotion regulation variability, except that the strategy switching subsequent no longer significantly predict changes in emotion differentiation in the subsequent moment (model 2B). In terms of individual difference, interestingly, in addition to the between-person negative association between negative emotion differentiation and endorsement change, there was a positive association between negative emotion differentiation and strategy switching (model 2B). In other words, the degree to which participants switched from one strategy to

another on average was positively related to their baseline negative emotion differentiation. In summary, the relations between negative emotion differentiation and emotion regulation variability subcomponents were also largely robust.

Sensitivity analyses of exploratory models on positive emotion differentiation showed that relations between positive emotion differentiation and emotion regulation variability were less robust than those between negative emotion differentiation and emotion regulation variability. Higher positive emotion differentiation preceded lower emotion regulation variability (model 1A) and specifically lower endorsement change (model 1C). Other than these, no other within-person temporal relations or between-person relations were found (model 1B, 2A, and 2B).

In the sensitivity analyses of the exploratory within-person mediation models (Model 1M), the direct paths results were consistent in direction and statistical significance with our main analyses. As a preparatory step for evaluating the indirect path, we reanalyzed the model using the *lme4* package under the successive temporal comparison approach specification. However, the lmer estimates for the b-path and c'-path in Model 1M (negative emotion) were in the opposite direction compared to our main analyses. This discrepancy may stem from the exclusion of the autocorrelated residual term, which was based on a lag-one temporal relation similar to the successive temporal comparison and becomes highly influential in the successive temporal comparison approach to operationalizing emotion regulation variability. Consequently, we were unable to estimate the confidence intervals for the indirect paths.

Table S6

Fixed Effect Estimates of Within-Person Temporal Associations and Between-Person Differences in Between Emotion Differentiation, Emotion Intensity, and Emotion Regulation Variability Calculated as the Successive Comparison Approach

	Negative Emotions *b* [95% *CI]*	Positive Emotions *b* [95% *CI]*
Outcome: Emotion regulation variability (Model 1A)	N = 678, n = 25522	N = 677, n = 25502
Within-person (time-varying)		
**Lagged emotion differentiation**	**-0.017 [-0.025, -0.010]**	**-0.021 [-0.039, -0.003]**
Lagged emotion intensity	-0.031 [-0.198, 0.136]	-0.006 [-0.051, 0.038]
Emotion regulation intensity	0.027 [-0.322, 0.376]	0.017 [-0.328, 0.361]
**Time trend**	**-0.006 [-0.008, -0.005]**	**-0.006 [-0.008, -0.004]**
Between-person (time-invariant)		
**Intercept**	**3.330 [2.293, 4.368]**	**3.145 [2.043, 4.247]**
Emotion differentiation	0.078 [-0.047, 0.204]	-0.020 [-0.214, 0.174]
Emotion intensity	0.014 [-0.083, 0.110]	-0.058 [-0.125, 0.009]
**Emotion regulation intensity**	**-0.504 [-0.573, -0.435]**	**-0.508 [-0.571, -0.445]**
Age	-0.002 [-0.053, 0.049]	0.008 [-0.047, 0.064]
**Gender (female = 1, male = 0)**	**0.240 [0.041, 0.440]**	**0.241 [0.036, 0.447]**
Outcome: Strategy switching (Model 1B)	N = 678, n = 25522	N = 677, n = 25502
Within-person (time-varying)		
**Endorsement change**	**-0.382 [-0.488, -0.275]**	**-0.380 [-0.484, -0.276]**
**Lagged emotion differentiation**	**-0.009 [-0.016, -0.002]**	-0.007 [-0.019, 0.005]
Lagged emotion intensity	-0.027 [-0.117, 0.062]	-0.007 [-0.041, 0.026]
Emotion regulation intensity	-0.071 [-0.154, 0.013]	-0.073 [-0.164, 0.018]
**Time trend**	**-0.004 [-0.005, -0.003]**	**-0.004 [-0.005, -0.002]**
Between-person (time-invariant)		
**Intercept**	**1.513 [1.035, 1.991]**	**1.470 [0.995, 1.944]**
**Endorsement change**	**0.092 [0.056, 0.128]**	**0.090 [0.054, 0.126]**
**Emotion differentiation**	**0.098 [0.044, 0.152]**	0.070 [-0.016, 0.155]
Emotion intensity	0.000 [-0.047, 0.047]	-0.017 [-0.045, 0.011]
Emotion regulation intensity	0.005 [-0.030, 0.040]	-0.008 [-0.039, 0.024]
Age	-0.002 [-0.018, 0.014]	0.001 [-0.016, 0.017]
Gender (female = 1, male = 0)	0.085 [-0.001, 0.171]	0.084 [-0.003, 0.170]
Outcome: Endorsement change (Model 1C)	N = 678, n = 25522	N = 677, n = 25502

Table S6

Fixed Effect Estimates of Within-Person Temporal Associations and Between-Person Differences in Between Emotion Differentiation, Emotion Intensity, and Emotion Regulation Variability Calculated as the Successive Comparison Approach (continued)

	Negative Emotions *b* [95% *CI]*	Positive Emotions *b* [95% *CI]*
Within-person (time-varying)		
**Strategy switching**	**-0.487 [-0.525, -0.449]**	**-0.486 [-0.522, -0.451]**
**Lagged emotion differentiation**	**-0.015 [-0.022, -0.008]**	**-0.020 [-0.036, -0.005]**
Lagged emotion intensity	-0.040 [-0.177, 0.096]	0.004 [-0.029, 0.037]
Emotion regulation intensity	-0.017 [-0.303, 0.270]	-0.027 [-0.319, 0.264]
**Time trend**	**-0.005 [-0.007, -0.004]**	**-0.005 [-0.007, -0.003]**
Between-person (time-invariant)		
**Intercept**	**1.446 [0.725, 2.167]**	**1.507 [0.788, 2.227]**
**Strategy switching**	**0.108 [0.036, 0.180]**	**0.090 [0.018, 0.162]**
Emotion differentiation	-0.011 [-0.096, 0.073]	-0.074 [-0.203, 0.055]
Emotion intensity	-0.052 [-0.119, 0.014]	-0.008 [-0.052, 0.035]
**Emotion regulation intensity**	**-0.325 [-0.374, -0.276]**	**-0.347 [-0.391, -0.304]**
Age	0.022 [-0.016, 0.060]	0.019 [-0.019, 0.056]
Gender (female = 1, male = 0)	0.089 [-0.042, 0.219]	0.088 [-0.044, 0.220]
Within-person mediation (Model 1M)	N = 682, n = 51338	N = 681, n = 51305
Within-person (time-varying)		
**Lagged emotion differentiation → emotion regulation variability (a-path)**	**-0.027 [-0.035, -0.019]**	**-0.027 [-0.040, -0.013]**
Lagged emotion intensity → emotion regulation variability	-0.110 [-0.312, 0.092]	0.004 [-0.054, 0.062]
**Time trend → emotion regulation variability**	**-0.006 [-0.008, -0.005]**	**-0.006 [-0.007, -0.004]**
**Emotion regulation variability → emotion intensity (b-path)**	**0.026 [0.020, 0.032]**	-0.013 [-0.028, 0.003]
**Emotion regulation intensity → emotion intensity**	**0.257 [0.196, 0.318]**	**-0.127 [-0.225, -0.029]**
**Lagged emotion intensity → emotion intensity**	**0.265 [0.243, 0.287]**	**0.310 [0.253, 0.368]**
**Lagged emotion differentiation → emotion intensity (c'-path)**	**0.009 [0.006, 0.012]**	**-0.018 [-0.028, -0.008]**
**Time trend → emotion intensity**	**-0.002 [-0.003, -0.001]**	**-0.002 [-0.003, -0.001]**
Within-person mediation (Model 1M) with person-level emotion differentiation as a moderator to a-path and b-path	N = 682, n = 51338	N = 681, n = 51305
Within-person (time-varying)		

Table S6

Fixed Effect Estimates of Within-Person Temporal Associations and Between-Person Differences in Between Emotion Differentiation, Emotion Intensity, and Emotion Regulation Variability Calculated as the Successive Comparison Approach (continued)

	Negative Emotions *b* [95% CI]	Positive Emotions *b* [95% CI]
**Lagged emotion differentiation → emotion regulation variability (a-path)**	**-0.031 [-0.040, -0.022]**	**-0.032 [-0.055, -0.010]**
Lagged emotion intensity → emotion regulation variability	-0.111 [-0.356, 0.134]	0.008 [-0.047, 0.062]
**Time trend → emotion regulation variability**	**-0.006 [-0.008, -0.005]**	**-0.006 [-0.007, -0.004]**
**Emotion regulation variability → emotion intensity (b-path)**	**0.027 [0.021, 0.033]**	-0.012 [-0.028, 0.003]
**Emotion regulation intensity → emotion intensity**	**0.255 [0.186, 0.324]**	**-0.127 [-0.228, -0.025]**
**Lagged emotion intensity → emotion intensity**	**0.268 [0.193, 0.344]**	**0.311 [0.252, 0.370]**
**Lagged emotion differentiation → emotion intensity (c'-path)**	**0.009 [0.005, 0.013]**	**-0.018 [-0.029, -0.008]**
**Time trend → emotion intensity**	**-0.002 [-0.002, -0.001]**	**-0.002 [-0.003, -0.001]**
**Lagged emotion differentiation → emotion regulation variability (a-path), moderated by between-person emotion differentiation**	**-0.014 [-0.022, -0.006]**	-0.016 [-0.038, 0.007]
Emotion regulation variability → emotion intensity (b-path), moderated by between-person emotion differentiation	0.000 [-0.007, 0.006]	0.015 [-0.002, 0.032]
Outcome: Emotion differentiation (Model 2A)	N = 678, n = 25510	N = 673, n = 25402
Within-person (time-varying)		
**Emotion regulation variability**	**-0.087 [-0.135, -0.038]**	0.005 [-0.011, 0.021]
**Lagged emotion differentiation**	**-0.022 [-0.034, -0.009]**	0.026 [-0.006, 0.057]
**Emotion intensity**	**-4.415 [-5.598, -3.233]**	**0.671 [0.422, 0.920]**
Emotion regulation intensity	0.074 [-0.006, 0.154]	-0.040 [-0.093, 0.013]
**Time trend**	**-0.005 [-0.008, -0.003]**	**0.004 [0.002, 0.006]**
Between-person (time-invariant)		
**Intercept**	**-1.611 [-2.247, -0.975]**	-0.077 [-0.691, 0.537]
Emotion regulation variability	-0.017 [-0.057, 0.024]	-0.006 [-0.036, 0.023]
**Emotion intensity**	**-0.238 [-0.299, -0.177]**	**0.035 [0.004, 0.065]**
**Emotion regulation intensity**	**-0.047 [-0.092, -0.001]**	-0.011 [-0.041, 0.018]
Age	-0.029 [-0.064, 0.006]	**-0.068 [-0.099, -0.036]**
Gender (female = 1, male = 0)	0.068 [-0.058, 0.193]	**-0.157 [-0.248, -0.065]**
Outcome: Emotion differentiation (Model 2B)	N = 678, n = 25510	N = 673, n = 25402
Within-person (time-varying)		

Table S6

Fixed Effect Estimates of Within-Person Temporal Associations and Between-Person Differences in Between Emotion Differentiation, Emotion Intensity, and Emotion Regulation Variability Calculated as the Successive Comparison Approach (continued)

	Negative Emotions *b* [95% *CI]*	Positive Emotions *b* [95% *CI]*
Strategy switching	-0.065 [-0.145, 0.014]	0.017 [-0.004, 0.039]
**Endorsement change**	**-0.099 [-0.147, -0.051]**	0.000 [-0.017, 0.017]
**Lagged emotion differentiation**	**-0.022 [-0.035, -0.009]**	0.025 [-0.006, 0.057]
**Emotion intensity**	**-4.399 [-5.535, -3.264]**	**0.672 [0.423, 0.921]**
Emotion regulation intensity	0.072 [-0.005, 0.149]	-0.040 [-0.093, 0.014]
**Time trend**	**-0.005 [-0.008, -0.003]**	**0.004 [0.002, 0.006]**
Between-person (time-invariant)		
**Intercept**	**-1.659 [-2.318, -1.001]**	-0.100 [-0.711, 0.512]
Strategy switching	0.069 [-0.003, 0.141]	0.019 [-0.034, 0.072]
**Endorsement change**	**-0.081 [-0.141, -0.021]**	-0.025 [-0.068, 0.019]
**Emotion intensity**	**-0.242 [-0.303, -0.181]**	**0.035 [0.005, 0.065]**
**Emotion regulation intensity**	**-0.067 [-0.115, -0.019]**	-0.018 [-0.051, 0.014]
Age	-0.026 [-0.062, 0.010]	**-0.066 [-0.098, -0.035]**
Gender (female = 1, male = 0)	0.060 [-0.065, 0.185]	**-0.157 [-0.249, -0.066]**

Note: Significant effects are displayed in bold. n: number of ESM assessments; N: number of adolescents; b: unstandardized effect; CI: confidence interval. In Model 1M, n is doubled because of how data have undergone the stacking preparation step.

Supplemental Materials 7: Sensitivity analyses on the potential influence of zero negative emotion intensity and zero emotion regulation intensity

Supplemental Materials 3 indicated that negative emotion intensity and emotion regulation intensity may experience some extent of floor effects. To address whether the presence of zero-intensity moments (where all negative emotions or regulation strategies were rated zero) confounded our main findings, we conducted sensitivity analyses as described in the below paragraphs. Positive emotions were excluded from these analyses, as descriptive statistics did not indicate the presence of floor effects in them.

We first created binary variables to indicate the presence of zero-intensity moments for negative emotions or emotion regulation strategies (first half of Table S7). These binary variables were multiplied by the within-person components of the independent variables in all models (e.g., emotion differentiation in Model 1A) to generate within-person moderators. All models (1A to 2B, for both positive and negative emotions) were then reanalyzed with the binary variables and within-person moderators included. Random effects for these within-person moderators correspondingly specified.

We ran a second set of sensitivity analyses using the within-person components of negative emotion intensity and emotion regulation intensity from our original models (second half of Table S7). These intensity variables were multiplied by the independent variables (e.g., the product of within-person negative emotion intensity and negative emotion differentiation in Model 1A) to create new within-person moderators. The analyses for all models (1A to 2B) were repeated with these continuous moderators added, again with random effects for these within-person moderators correspondingly specified.

Across both sets of analyses, the main effects of interest (e.g., emotion differentiation in Model 1A) generally remained consistent in direction and statistical significance. An exception arose in Model 1M with binary zero-intensity moderators, where the a-path (emotion differentiation to regulation variability) and b-path (regulation

variability to emotion intensity) were no longer significant. However, in Model 1M with continuous intensity moderators, the direct paths remained significant, consistent with the main analyses. These findings indicate that our results are generally robust against the presence of zero intensity in negative emotions and emotion regulation strategies.

As a preparatory step for evaluating the indirect path in Model 1M, we reanalyzed Model 1M with the aforementioned specifications using the *lme4* package. However, we encountered convergence issue in the binary moderator model, making us unable to obtain any estimates. As for the continuous moderator model, extraction of asymptotic covariance of random effects encountered an error as the model became too complex for evaluating so. Consequently, we were unable to estimate the confidence intervals for the indirect paths.

Table S7

Fixed Effect Estimates of Within-Person Temporal Associations and Between-Person Differences in Between Emotion Differentiation, Emotion Regulation Variability and Emotion Intensity, Examing the Potential Influence of Zero Negative Emotion (Regulation) Intensity

	Negative Emotions *b* [95% *CI]*
Presence of zero emotion (regulation) intensity as moderator	
Outcome: Emotion regulation variability (Model 1A)	N = 752, n = 25867
Within-person (time-varying)	
Lagged emotion differentiation	**-0.009 [-0.013, -0.005]**
Lagged emotion intensity	-0.021 [-0.046, 0.004]
Emotion regulation intensity	0.290 [-0.286, 0.866]
Time trend	**-0.003 [-0.004, -0.002]**
When the intensity of all emotions is rated zero → emotion regulation variability	0.032 [-0.053, 0.117]
Lagged emotion differentiation, when the intensity of all emotions is rated zero	**-0.056 [-0.107, -0.005]**
Between-person (time-invariant)	
Intercept	**3.948 [2.812, 5.084]**
Emotion differentiation	0.056 [-0.083, 0.195]
Emotion intensity	-0.032 [-0.137, 0.074]

Table S7

Fixed Effect Estimates of Within-Person Temporal Associations and Between-Person Differences in Between Emotion Differentiation, Emotion Regulation Variability and Emotion Intensity, Examing the Potential Influence of Zero Negative Emotion (Regulation) Intensity (continued)

	Negative Emotions *b* [95% *CI]*
Emotion regulation intensity	**-0.555 [-0.632, -0.479]**
Age	-0.007 [-0.066, 0.053]
Gender (female = 1, male = 0)	**0.410 [0.186, 0.633]**
Outcome: Strategy switching (Model 1B)	N = 752, n = 25867
Within-person (time-varying)	
Endorsement change	**-0.435 [-0.575, -0.294]**
Lagged emotion differentiation	**-0.004 [-0.006, -0.001]**
Lagged emotion intensity	-0.010 [-0.024, 0.005]
Emotion regulation intensity	**-0.103 [-0.153, -0.053]**
Time trend	**-0.002 [-0.002, -0.001]**
When the intensity of all emotions is rated zero → emotion regulation variability	0.012 [-0.044, 0.068]
Lagged emotion differentiation, when the intensity of all emotions is rated zero	-0.018 [-0.047, 0.011]
Between-person (time-invariant)	
Intercept	**0.912 [0.283, 1.540]**
Endorsement change	0.014 [-0.029, 0.058]
Emotion differentiation	**0.151 [0.081, 0.222]**
Emotion intensity	0.029 [-0.025, 0.083]
Emotion regulation intensity	0.013 [-0.031, 0.056]
Age	**0.036 [0.006, 0.065]**
Gender (female = 1, male = 0)	**0.139 [0.027, 0.251]**
Outcome: Endorsement change (Model 1C)	N = 752, n = 25867
Within-person (time-varying)	
Strategy switching	0.311 [-1.121, 1.743]
Lagged emotion differentiation	**-0.007 [-0.011, -0.004]**
Lagged emotion intensity	-0.015 [-0.032, 0.001]
Emotion regulation intensity	0.057 [-0.227, 0.341]
Time trend	**-0.002 [-0.003, -0.002]**

Table S7

Fixed Effect Estimates of Within-Person Temporal Associations and Between-Person Differences in Between Emotion Differentiation, Emotion Regulation Variability and Emotion Intensity, Examing the Potential Influence of Zero Negative Emotion (Regulation) Intensity (continued)

	Negative Emotions *b* [95% *CI]*
When the intensity of all emotions is rated zero → emotion regulation variability	0.048 [-0.018, 0.114]
Lagged emotion differentiation, when the intensity of all emotions is rated zero	-0.052 [-0.106, 0.003]
Between-person (time-invariant)	
Intercept	**2.536 [1.690, 3.382]**
Strategy switching	**-0.239 [-0.323, -0.156]**
Emotion differentiation	-0.088 [-0.190, 0.014]
Emotion intensity	-0.074 [-0.150, 0.001]
Emotion regulation intensity	**-0.681 [-0.737, -0.625]**
Age	-0.015 [-0.061, 0.031]
Gender (female = 1, male = 0)	**0.219 [0.059, 0.378]**
Within-person mediation (Model 1M)	N = 756, n = 52003
Within-person (time-varying)	
When the intensity of all emotions is rated zero → emotion regulation variability	0.156 [-0.048, 0.359]
When the intensity of all emotion regulation strategies is rated zero → emotion intensity	**1.124 [0.337, 1.911]**
Lagged emotion differentiation → emotion regulation variability (a-path)	0.001 [-0.030, 0.033]
Lagged emotion intensity → emotion regulation variability	0.192 [-0.194, 0.579]
Time trend → emotion regulation variability	**-0.005 [-0.005, -0.004]**
Emotion regulation variability → emotion intensity (b-path)	0.002 [-0.036, 0.041]
Emotion regulation intensity → emotion intensity	**0.241 [0.174, 0.308]**
Lagged emotion intensity → emotion intensity	**0.342 [0.253, 0.430]**
Lagged emotion differentiation → emotion intensity (c'-path)	**0.012 [0.002, 0.022]**
Time trend → emotion intensity	**-0.002 [-0.003, -0.002]**
Lagged emotion differentiation → emotion regulation variability (a-path), when the intensity of all emotions is rated zero	0.159 [-0.029, 0.347]
Emotion regulation variability → emotion intensity (b-path), when the intensity of all emotion regulation strategies is rated zero	**-1.044 [-1.361, -0.726]**

Table S7

Fixed Effect Estimates of Within-Person Temporal Associations and Between-Person Differences in Between Emotion Differentiation, Emotion Regulation Variability and Emotion Intensity, Examing the Potential Influence of Zero Negative Emotion (Regulation) Intensity (continued)

	Negative Emotions *b* [95% *CI*]
Within-person mediation (Model 1M) with person-level emotion differentiation as a moderator to a-path and b-path	N = 756, n = 52003
Within-person (time-varying)	
When the intensity of all emotions is rated zero → emotion regulation variability	0.157 [-0.055, 0.368]
When the intensity of all emotion regulation strategies is rated zero → emotion intensity	**1.128 [0.300, 1.956]**
Lagged emotion differentiation → emotion regulation variability (a-path)	-0.002 [-0.033, 0.030]
Lagged emotion intensity → emotion regulation variability	0.184 [-0.216, 0.584]
Time trend → emotion regulation variability	**-0.005 [-0.005, -0.004]**
Emotion regulation variability → emotion intensity (b-path)	0.004 [-0.041, 0.049]
Emotion regulation intensity → emotion intensity	**0.242 [0.168, 0.316]**
Lagged emotion intensity → emotion intensity	**0.341 [0.247, 0.435]**
Lagged emotion differentiation → emotion intensity (c'-path)	**0.013 [0.001, 0.024]**
Time trend → emotion intensity	**-0.002 [-0.003, -0.002]**
Lagged emotion differentiation → emotion regulation variability (a-path), when the intensity of all emotions is rated zero	0.151 [-0.051, 0.352]
Lagged emotion differentiation → emotion regulation variability (a-path), moderated by between-person emotion differentiation	**-0.013 [-0.019, -0.007]**
Emotion regulation variability → emotion intensity (b-path), when the intensity of all emotion regulation strategies is rated zero	**-1.040 [-1.363, -0.717]**
Emotion regulation variability → emotion intensity (b-path), moderated by between-person emotion differentiation	**-0.034 [-0.059, -0.009]**
Outcome: Emotion differentiation (Model 2A)	N = 751, n = 25830
Within-person (time-varying)	
Emotion regulation variability	**-0.510 [-0.727, -0.292]**
Lagged emotion differentiation	**-0.019 [-0.032, -0.005]**
Emotion intensity	**-3.900 [-5.118, -2.682]**
Emotion regulation intensity	-0.037 [-0.133, 0.058]

Table S7

Fixed Effect Estimates of Within-Person Temporal Associations and Between-Person Differences in Between Emotion Differentiation, Emotion Regulation Variability and Emotion Intensity, Examing the Potential Influence of Zero Negative Emotion (Regulation) Intensity (continued)

	Negative Emotions *b* [95% *CI]*
Time trend	**-0.006 [-0.008, -0.003]**
When the intensity of all emotion regulation strategies is rated zero → emotion intensity	-0.412 [-0.883, 0.059]
Emotion regulation variability, when the intensity of all emotion regulation strategies is rated zero	0.205 [-0.040, 0.450]
Between-person (time-invariant)	
Intercept	**-1.014 [-1.701, -0.327]**
Emotion regulation variability	**-0.059 [-0.098, -0.019]**
Emotion intensity	**-0.260 [-0.314, -0.205]**
Emotion regulation intensity	**-0.123 [-0.175, -0.071]**
Age	**-0.053 [-0.089, -0.017]**
Gender (female = 1, male = 0)	0.040 [-0.073, 0.153]
Outcome: Emotion differentiation (Model 2B)	N = 751, n = 25830
Within-person (time-varying)	
Strategy switching	**-0.447 [-0.689, -0.205]**
Endorsement change	**-0.545 [-0.782, -0.307]**
Lagged emotion differentiation	**-0.016 [-0.031, -0.002]**
Emotion intensity	**-3.873 [-5.187, -2.559]**
Emotion regulation intensity	-0.043 [-0.148, 0.063]
Time trend	**-0.006 [-0.008, -0.004]**
When the intensity of all emotion regulation strategies is rated zero → emotion intensity	-0.091 [-0.646, 0.464]
Endorsement change, when the intensity of all emotion regulation strategies is rated zero	**0.323 [0.047, 0.598]**
Strategy switching, when the intensity of all emotion regulation strategies is rated zero	**0.594 [0.232, 0.956]**
Between-person (time-invariant)	
Intercept	**-0.927 [-1.595, -0.259]**

Table S7

Fixed Effect Estimates of Within-Person Temporal Associations and Between-Person Differences in Between Emotion Differentiation, Emotion Regulation Variability and Emotion Intensity, Examing the Potential Influence of Zero Negative Emotion (Regulation) Intensity (continued)

	Negative Emotions *b* [95% *CI*]
Strategy switching	-0.029 [-0.095, 0.037]
Endorsement change	**-0.069 [-0.127, -0.012]**
Emotion intensity	**-0.268 [-0.322, -0.213]**
Emotion regulation intensity	**-0.109 [-0.164, -0.054]**
Age	**-0.060 [-0.095, -0.025]**
Gender (female = 1, male = 0)	0.037 [-0.075, 0.150]
Within-person emotion (regulation) intensity as moderator	
Outcome: Emotion regulation variability (Model 1A)	N = 752, n = 25867
Within-person (time-varying)	
Lagged emotion differentiation	**-0.014 [-0.021, -0.007]**
Lagged emotion intensity	-0.016 [-0.040, 0.009]
Emotion regulation intensity	0.294 [-0.296, 0.884]
Time trend	**-0.003 [-0.004, -0.002]**
Lagged emotion differentiation, moderated by within-person emotion intensity	**0.002 [0.000, 0.003]**
Between-person (time-invariant)	
Intercept	**3.902 [2.823, 4.982]**
Emotion differentiation	0.062 [-0.078, 0.202]
Emotion intensity	-0.023 [-0.129, 0.082]
Emotion regulation intensity	**-0.552 [-0.629, -0.476]**
Age	-0.005 [-0.061, 0.051]
Gender (female = 1, male = 0)	**0.411 [0.187, 0.636]**
Outcome: Strategy switching (Model 1B)	N = 752, n = 25867
Within-person (time-varying)	
Endorsement change	**-0.435 [-0.576, -0.293]**
Lagged emotion differentiation	**-0.007 [-0.012, -0.002]**

Table S7

Fixed Effect Estimates of Within-Person Temporal Associations and Between-Person Differences in Between Emotion Differentiation, Emotion Regulation Variability and Emotion Intensity, Examing the Potential Influence of Zero Negative Emotion (Regulation) Intensity (continued)

	Negative Emotions *b* [95% *CI*]
Lagged emotion intensity	-0.009 [-0.023, 0.005]
Emotion regulation intensity	**-0.102 [-0.149, -0.056]**
Time trend	**-0.002 [-0.002, -0.001]**
Lagged emotion differentiation, moderated by within-person emotion intensity	0.001 [0.000, 0.002]
Between-person (time-invariant)	
Intercept	**0.867 [0.242, 1.492]**
Emotion differentiation	**0.147 [0.077, 0.218]**
Emotion intensity	0.029 [-0.025, 0.082]
Emotion regulation intensity	0.009 [-0.030, 0.048]
Age	**0.038 [0.009, 0.067]**
Gender (female = 1, male = 0)	**0.143 [0.032, 0.255]**
Outcome: Endorsement change (Model 1C)	N = 752, n = 25867
Within-person (time-varying)	
Strategy switching	0.295 [-1.136, 1.726]
Lagged emotion differentiation	**-0.011 [-0.016, -0.006]**
Lagged emotion intensity	-0.016 [-0.033, 0.002]
Emotion regulation intensity	0.049 [-0.232, 0.330]
Time trend	**-0.002 [-0.003, -0.002]**
Lagged emotion differentiation, moderated by within-person emotion intensity	**0.001 [0.000, 0.002]**
Between-person (time-invariant)	
Intercept	**2.435 [1.546, 3.323]**
Emotion differentiation	**-0.120 [-0.223, -0.016]**
Emotion intensity	-0.074 [-0.152, 0.003]
Emotion regulation intensity	**-0.653 [-0.709, -0.596]**
Age	-0.008 [-0.056, 0.040]
Gender (female = 1, male = 0)	**0.190 [0.026, 0.355]**
Within-person mediation (Model 1M)	N = 756, n = 52003

Table S7

Fixed Effect Estimates of Within-Person Temporal Associations and Between-Person Differences in Between Emotion Differentiation, Emotion Regulation Variability and Emotion Intensity, Examing the Potential Influence of Zero Negative Emotion (Regulation) Intensity (continued)

	Negative Emotions *b* [95% *CI*]
Within-person (time-varying)	
Lagged emotion differentiation → emotion regulation variability (a-path)	**-0.026 [-0.033, -0.018]**
Lagged emotion intensity → emotion regulation variability	-0.025 [-0.085, 0.034]
Time trend → emotion regulation variability	**-0.005 [-0.006, -0.004]**
Emotion regulation variability → emotion intensity (b-path)	**0.085 [0.049, 0.120]**
Emotion regulation intensity → emotion intensity	**0.220 [0.142, 0.298]**
Lagged emotion intensity → emotion intensity	**0.261 [0.203, 0.318]**
Lagged emotion differentiation → emotion intensity (c'-path)	**0.009 [0.004, 0.014]**
Time trend → emotion intensity	**-0.002 [-0.002, -0.001]**
Lagged emotion differentiation → emotion regulation variability (a-path), moderated by within-person emotion intensity	**0.004 [0.003, 0.006]**
Emotion regulation variability → emotion intensity (b-path), moderated by within-person emotion regulation intensity	**0.023 [0.011, 0.035]**
Within-person mediation (Model 1M) with person-level emotion differentiation as a moderator to a-path and b-path	N = 756, n = 52003
Within-person (time-varying)	
Lagged emotion differentiation → emotion regulation variability (a-path)	**-0.025 [-0.032, -0.019]**
Lagged emotion intensity → emotion regulation variability	-0.026 [-0.085, 0.033]
Time trend → emotion regulation variability	**-0.005 [-0.006, -0.004]**
Emotion regulation variability → emotion intensity (b-path)	**0.084 [0.052, 0.117]**
Emotion regulation intensity → emotion intensity	**0.222 [0.147, 0.297]**
Lagged emotion intensity → emotion intensity	**0.262 [0.207, 0.317]**
Lagged emotion differentiation → emotion intensity (c'-path)	**0.009 [0.004, 0.014]**
Time trend → emotion intensity	**-0.002 [-0.002, -0.001]**
Lagged emotion differentiation → emotion regulation variability (a-path), moderated by within-person emotion intensity	**0.004 [0.002, 0.005]**
Lagged emotion differentiation → emotion regulation variability (a-path), moderated by between-person emotion differentiation	-0.003 [-0.007, 0.001]

Table S7

Fixed Effect Estimates of Within-Person Temporal Associations and Between-Person Differences in Between Emotion Differentiation, Emotion Regulation Variability and Emotion Intensity, Examing the Potential Influence of Zero Negative Emotion (Regulation) Intensity (continued)

	Negative Emotions *b* [95% *CI]*
Emotion regulation variability → emotion intensity (b-path), moderated by within-person emotion regulation intensity	**0.023 [0.012, 0.035]**
Emotion regulation variability → emotion intensity (b-path), moderated by between-person emotion differentiation	-0.016 [-0.039, 0.008]
Outcome: Emotion differentiation (Model 2A)	N = 751, n = 25830
Within-person (time-varying)	
Emotion regulation variability	**-0.524 [-0.829, -0.219]**
Lagged emotion differentiation	-0.012 [-0.026, 0.003]
Emotion intensity	**-3.788 [-4.792, -2.784]**
Emotion regulation intensity	**0.096 [0.007, 0.186]**
Time trend	**-0.005 [-0.008, -0.003]**
Emotion regulation variability, moderated by within-person emotion regulation intensity	**-0.269 [-0.400, -0.138]**
Between-person (time-invariant)	
Intercept	**-1.118 [-1.819, -0.417]**
Emotion regulation variability	**-0.056 [-0.094, -0.019]**
Emotion intensity	**-0.191 [-0.252, -0.131]**
Emotion regulation intensity	**-0.078 [-0.124, -0.033]**
Age	**-0.050 [-0.088, -0.013]**
Gender (female = 1, male = 0)	0.040 [-0.085, 0.166]
Outcome: Emotion differentiation (Model 2B)	N = 751, n = 25830
Within-person (time-varying)	
Strategy switching	**-0.473 [-0.820, -0.127]**
Endorsement change	**-0.636 [-0.971, -0.302]**
Lagged emotion differentiation	-0.009 [-0.023, 0.006]
Emotion intensity	**-3.687 [-4.772, -2.603]**
Emotion regulation intensity	0.078 [-0.022, 0.178]

Table S7

Fixed Effect Estimates of Within-Person Temporal Associations and Between-Person Differences in Between Emotion Differentiation, Emotion Regulation Variability and Emotion Intensity, Examing the Potential Influence of Zero Negative Emotion (Regulation) Intensity (continued)

	Negative Emotions *b* [95% *CI]*
Time trend	**-0.006 [-0.008, -0.004]**
Endorsement change, moderated by within-person emotion regulation intensity	**-0.300 [-0.434, -0.165]**
Strategy switching, moderated by within-person emotion regulation intensity	**-0.332 [-0.483, -0.182]**
Between-person (time-invariant)	
Intercept	**-1.173 [-1.833, -0.513]**
Strategy switching	-0.030 [-0.096, 0.036]
Endorsement change	**-0.079 [-0.133, -0.025]**
Emotion intensity	**-0.184 [-0.244, -0.124]**
Emotion regulation intensity	**-0.087 [-0.135, -0.039]**
Age	**-0.049 [-0.084, -0.013]**
Gender (female = 1, male = 0)	0.048 [-0.076, 0.172]

Note: Significant effects are displayed in bold. n: number of ESM assessments; N: number of adolescents; b: unstandardized effect; CI: confidence interval. In Model 1M, n is doubled because of how data have undergone the stacking preparation step.

Supplemental Materials 8: Potential influence of age: Dataset-specific effects and sensitivity analyses

In this section, we first present how within-person effects in our preregistered analyses vary across datasets (Table S8.1). Following this, we explore whether within-dataset age differences moderated the within-person effects of interest through sensitivity analyses.

Dataset-specific effects

Table S8.1 reveals that within-person results appeared stronger in datasets sampling late adolescents. This pattern suggests indicative evidence of age moderation in the within-person effects we studied. Indicative, because we cannot tease apart the influence of age differences from other study design features. In other words, the differences in strength of within-person results could have possibly been caused by study design features instead of age differences.

Table S8.1

Dataset-specific effects, given by sum of dataset-level random effects and fixed effects

Model	Index or path	Outside-in (Ghent)	G(F)ood together (Radboud)	3-wave longitudinal study (Leuven)	Emotions in daily life 2011 (Leuven)	Emotions in daily life (Tilburg)
	Age mean	13.486	16.434	18.322	19.053	20.879
	Age standard deviation	0.578	0.684	0.957	1.275	1.701
Model 1A (Negative emotion; Outcome: emotion regulation variability)	Lagged emotion differentiation	-0.008	-0.009	-0.013	-0.012	-0.005
Model 1B (Negative emotion; Outcome: strategy switching)	Lagged emotion differentiation	-0.003	-0.004	-0.005	-0.006	-0.002
Model 1C (Negative emotion; Outcome: endorsement change)	Lagged emotion differentiation	-0.003	-0.010	-0.010	-0.010	-0.007
Model 2A (Outcome: Negative emotion differentiation)	Emotion regulation variability	-0.193	-0.487	-0.605	-0.681	-0.605
Model 2B (Outcome: Negative emotion differentiation)	Endorsement change	-0.245	-0.473	-0.647	-0.752	-0.631
Model 2B (Outcome: Negative emotion differentiation)	Strategy switching	0.036	-0.380	-0.571	-0.678	-0.565
Model 1A (Positive emotion; Outcome: emotion regulation variability)	Lagged emotion differentiation	-0.004	-0.011	-0.012	-0.012	-0.008
Model 1B (Positive emotion; Outcome: strategy switching)	Lagged emotion differentiation	-0.002	-0.003	-0.005	-0.006	-0.002
Model 1C (Positive emotion; Outcome: endorsement change)	Lagged emotion differentiation	-0.002	-0.009	-0.010	-0.008	-0.006
Model 2A (Outcome: Positive emotion differentiation)	Emotion regulation variability	-0.084	-0.107	-0.341	-0.179	-0.671
Model 2B (Outcome: Positive emotion differentiation)	Endorsement change	-0.095	-0.117	-0.319	-0.161	-0.622
Model 2B (Outcome: Positive emotion differentiation)	Strategy switching	-0.055	-0.118	-0.368	-0.292	-0.814
Model 1M (Negative emotion)	Lagged emotion differentiation → emotion regulation variability (a-path)	-0.009	-0.011	-0.016	-0.016	-0.012
Model 1M (Negative emotion)	Emotion regulation variability → emotion intensity (b-path)	0.092	0.100	0.050	0.030	0.092
Model 1M (Negative emotion)	Lagged emotion differentiation → emotion intensity (c'-path)	0.008	0.004	0.010	0.014	0.005
Model 1M (Positive emotion)	Lagged emotion differentiation → emotion regulation variability (a-path)	-0.013	-0.015	-0.016	-0.015	-0.015
Model 1M (Positive emotion)	Emotion regulation variability → emotion intensity (b-path)	-0.117	-0.055	-0.003	-0.029	-0.026
Model 1M (Positive emotion)	Lagged emotion differentiation → emotion intensity (c'-path)	-0.017	-0.018	-0.019	-0.019	-0.017

Sensitivity Analyses on Within-Dataset Age Differences

To further examine the role of age, we created a new variable, dataset-centered age, representing age differences within each dataset. Between-dataset age differences were already accounted for in the dataset-level random slopes. We calculated within-person moderators by multiplying the dataset-centered age with the within-person components of the independent variables (e.g., emotion differentiation in Model 1A). These moderators were added to all models (1A to 2B, positive and negative emotions), replacing the original age variable. To prevent the model from being overly complex for R packages' evaluation, random effects for the within-person moderators were not included.

Our analyses showed that the main effects of primary interest (e.g., emotion differentiation in Model 1A) remained consistent in direction and statistical significance across models. The only exception was the b-path (from emotion regulation variability to positive emotion intensity) in Model 1M (positive emotion), where the estimate maintained its original direction but its 95% confidence interval narrowly crossed zero into the positive range (0.000). These findings suggest that our results are generally robust against within-dataset age differences. Regardless of the dataset-level variations in and within-dataset age differences' on strength of within-person effects, we can still conclude there are fixed effects across the participants from the five datasets we studied on the within-person processes we hypothesized on.

Table S8.2

Fixed Effect Estimates of Within-Person Temporal Associations and Between-Person Differences in Between Emotion Differentiation, Emotion Regulation Variability, and Emotion Intensity, with Within-Dataset Age Differences as a Moderator

	Negative Emotions *b* [95% *CI]*	Positive Emotions *b* [95% *CI]*
Outcome: Emotion regulation variability (Model 1A)	N = 752, n = 25867	N = 751, n = 25851
Within-person (time-varying)		
Lagged emotion differentiation	**-0.009 [-0.014, -0.005]**	**-0.009 [-0.014, -0.004]**
Lagged emotion differentiation, moderated by within-dataset age difference	-0.001 [-0.003, 0.002]	-0.002 [-0.005, 0.001]
Lagged emotion intensity	-0.018 [-0.044, 0.008]	-0.005 [-0.017, 0.007]
Emotion regulation intensity	0.294 [-0.285, 0.874]	0.280 [-0.275, 0.834]
Time trend	**-0.003 [-0.004, -0.003]**	**-0.003 [-0.004, -0.002]**
Between-person (time-invariant)		
Intercept	**3.817 [3.347, 4.287]**	**3.848 [3.386, 4.310]**
Emotion differentiation	0.069 [-0.071, 0.209]	-0.049 [-0.255, 0.158]
Emotion intensity	-0.022 [-0.128, 0.084]	**-0.107 [-0.181, -0.034]**
Emotion regulation intensity	**-0.553 [-0.630, -0.476]**	**-0.561 [-0.630, -0.492]**
Within-dataset age difference	0.010 [-0.086, 0.106]	0.009 [-0.087, 0.105]
Gender (female = 1, male = 0)	**0.410 [0.186, 0.634]**	**0.347 [0.120, 0.575]**
Outcome: Strategy switching (Model 1B)	N = 752, n = 25867	N = 751, n = 25851
Within-person (time-varying)		
Endorsement change	**-0.439 [-0.577, -0.301]**	**-0.440 [-0.572, -0.307]**
Lagged emotion differentiation	**-0.004 [-0.007, -0.002]**	**-0.004 [-0.007, -0.001]**
Lagged emotion differentiation, moderated by within-dataset age difference	**-0.002 [-0.003, 0.000]**	-0.002 [-0.004, 0.001]
Lagged emotion intensity	-0.010 [-0.025, 0.005]	-0.002 [-0.013, 0.009]
Emotion regulation intensity	**-0.102 [-0.152, -0.052]**	**-0.102 [-0.152, -0.052]**
Time trend	**-0.002 [-0.002, -0.001]**	**-0.002 [-0.002, -0.001]**
Between-person (time-invariant)		
Intercept	**1.535 [1.110, 1.959]**	**1.544 [1.138, 1.950]**
Endorsement change	0.017 [-0.027, 0.061]	0.008 [-0.036, 0.052]
Emotion differentiation	**0.154 [0.084, 0.225]**	0.016 [-0.091, 0.122]
Emotion intensity	0.031 [-0.023, 0.085]	-0.036 [-0.073, 0.002]
Emotion regulation intensity	0.014 [-0.029, 0.058]	0.011 [-0.029, 0.052]

Table S8.2

Fixed Effect Estimates of Within-Person Temporal Associations and Between-Person Differences in Between Emotion Differentiation, Emotion Regulation Variability, and Emotion Intensity, with Within-Dataset Age Differences as a Moderator (continued)

	Negative Emotions *b* [95% *CI]*	Positive Emotions *b* [95% *CI]*
Within-dataset age difference	0.026 [-0.023, 0.074]	0.023 [-0.025, 0.072]
Gender (female = 1, male = 0)	**0.138 [0.025, 0.251]**	**0.124 [0.009, 0.239]**
Outcome: Endorsement change (Model 1C)	N = 752, n = 25867	N = 751, n = 25851
Within-person (time-varying)		
Strategy switching	0.312 [-1.138, 1.762]	0.303 [-1.136, 1.741]
Lagged emotion differentiation	**-0.008 [-0.012, -0.004]**	**-0.007 [-0.012, -0.003]**
Lagged emotion differentiation, moderated by within-dataset age difference	0.001 [-0.001, 0.002]	-0.001 [-0.004, 0.001]
Lagged emotion intensity	-0.017 [-0.034, 0.000]	-0.004 [-0.012, 0.004]
Emotion regulation intensity	0.053 [-0.234, 0.340]	0.058 [-0.228, 0.344]
Time trend	**-0.002 [-0.003, -0.002]**	**-0.002 [-0.003, -0.001]**
Between-person (time-invariant)		
Intercept	**2.275 [1.996, 2.554]**	**2.278 [2.004, 2.551]**
Strategy switching	**-0.234 [-0.318, -0.150]**	**-0.238 [-0.322, -0.154]**
Emotion differentiation	-0.081 [-0.183, 0.021]	-0.145 [-0.293, 0.004]
Emotion intensity	-0.071 [-0.147, 0.005]	0.025 [-0.028, 0.078]
Emotion regulation intensity	**-0.677 [-0.733, -0.621]**	**-0.695 [-0.746, -0.644]**
Within-dataset age difference	0.007 [-0.061, 0.076]	0.005 [-0.063, 0.073]
Gender (female = 1, male = 0)	**0.215 [0.054, 0.376]**	**0.204 [0.041, 0.367]**
Within-person mediation (Model 1M)	N = 752, n = 51697	N = 751, n = 51685
Within-person (time-varying)		
Lagged emotion differentiation → emotion regulation variability (a-path)	**-0.013 [-0.018, -0.008]**	**-0.014 [-0.020, -0.008]**
Lagged emotion intensity → emotion regulation variability	-0.029 [-0.082, 0.024]	-0.005 [-0.026, 0.016]
Time trend → emotion regulation variability	**-0.005 [-0.006, -0.004]**	**-0.005 [-0.005, -0.004]**
Emotion regulation variability → emotion intensity (b-path)	**0.073 [0.039, 0.108]**	**-0.049 [-0.092, -0.006]**
Emotion regulation intensity → emotion intensity	**0.234 [0.166, 0.302]**	**-0.101 [-0.189, -0.013]**
Lagged emotion intensity → emotion intensity	**0.258 [0.202, 0.315]**	**0.300 [0.249, 0.350]**
Lagged emotion differentiation → emotion intensity (c'-path)	**0.008 [0.003, 0.013]**	**-0.016 [-0.026, -0.006]**
Time trend → emotion intensity	**-0.002 [-0.002, -0.001]**	**-0.002 [-0.003, -0.001]**

Table S8.2

Fixed Effect Estimates of Within-Person Temporal Associations and Between-Person Differences in Between Emotion Differentiation, Emotion Regulation Variability, and Emotion Intensity, with Within-Dataset Age Differences as a Moderator (continued)

	Negative Emotions *b* [95% *CI]*	Positive Emotions *b* [95% *CI]*
Lagged emotion differentiation → emotion regulation variability (a-path), moderated by within-dataset age difference	-0.001 [-0.004, 0.002]	-0.004 [-0.009, 0.000]
Emotion regulation variability → emotion intensity (b-path), moderated by within-dataset age difference	-0.010 [-0.027, 0.007]	0.011 [-0.010, 0.033]
Mediation (sum of covariance and product of a- and b-path)	-0.000 [-0.001, 0.000]	-0.000 [-0.001, 0.001]
Within-person mediation (Model 1M) with person-level emotion differentiation as a moderator to a-path and b-path	N = 752, n = 51697	N = 751, n = 51685
Within-person (time-varying)		
Lagged emotion differentiation → emotion regulation variability (a-path)	**-0.015 [-0.020, -0.010]**	**-0.016 [-0.022, -0.010]**
Lagged emotion intensity → emotion regulation variability	-0.030 [-0.084, 0.023]	-0.005 [-0.028, 0.018]
Time trend → emotion regulation variability	**-0.005 [-0.006, -0.004]**	**-0.005 [-0.005, -0.004]**
Emotion regulation variability → emotion intensity (b-path)	**0.074 [0.038, 0.111]**	**-0.048 [-0.094, -0.003]**
Emotion regulation intensity → emotion intensity	**0.236 [0.164, 0.307]**	**-0.101 [-0.200, -0.003]**
Lagged emotion intensity → emotion intensity	**0.258 [0.201, 0.315]**	**0.300 [0.250, 0.350]**
Lagged emotion differentiation → emotion intensity (c'-path)	**0.008 [0.003, 0.014]**	**-0.016 [-0.025, -0.007]**
Time trend → emotion intensity	**-0.002 [-0.002, -0.001]**	**-0.002 [-0.003, -0.001]**
Lagged emotion differentiation → emotion regulation variability (a-path), moderated by between-person emotion differentiation	**-0.006 [-0.009, -0.002]**	**-0.011 [-0.021, 0.000]**
Lagged emotion differentiation → emotion regulation variability (a-path), moderated by within-dataset age difference	-0.001 [-0.004, 0.002]	**-0.005 [-0.009, 0.000]**
Emotion regulation variability → emotion intensity (b-path), moderated by between-person emotion differentiation	**-0.034 [-0.057, -0.011]**	**0.049 [0.000, 0.097]**
Emotion regulation variability → emotion intensity (b-path), moderated by within-dataset age difference	-0.011 [-0.028, 0.006]	0.013 [-0.009, 0.035]
Outcome: Emotion differentiation (Model 2A)	N = 751, n = 25830	N = 750, n = 25834
Within-person (time-varying)		
Emotion regulation variability	**-0.504 [-0.717, -0.290]**	**-0.282 [-0.517, -0.047]**
Emotion regulation variability, moderated by within-dataset age difference	-0.014 [-0.093, 0.064]	-0.028 [-0.074, 0.018]
Lagged emotion differentiation	**-0.018 [-0.031, -0.006]**	0.030 [0.000, 0.061]

Table S8.2

Fixed Effect Estimates of Within-Person Temporal Associations and Between-Person Differences in Between Emotion Differentiation, Emotion Regulation Variability, and Emotion Intensity, with Within-Dataset Age Differences as a Moderator (continued)

	Negative Emotions *b* [95% *CI]*	Positive Emotions *b* [95% *CI]*
Emotion intensity	**-3.885 [-5.056, -2.714]**	**0.528 [0.200, 0.855]**
Emotion regulation intensity	-0.028 [-0.110, 0.054]	**-0.153 [-0.253, -0.053]**
Time trend	**-0.006 [-0.008, -0.004]**	**0.004 [0.003, 0.006]**
Between-person (time-invariant)		
Intercept	**-2.034 [-2.265, -1.804]**	**-1.754 [-2.269, -1.238]**
Emotion regulation variability	-0.035 [-0.071, 0.001]	-0.012 [-0.039, 0.015]
Emotion intensity	**-0.237 [-0.296, -0.179]**	**0.034 [0.004, 0.064]**
Emotion regulation intensity	-0.043 [-0.087, 0.001]	-0.015 [-0.044, 0.015]
Within-dataset age difference	-0.037 [-0.088, 0.014]	**-0.078 [-0.117, -0.040]**
Gender (female = 1, male = 0)	0.044 [-0.076, 0.165]	**-0.150 [-0.240, -0.059]**
Outcome: Emotion differentiation (Model 2B)	N = 751, n = 25830	N = 750, n = 25834
Within-person (time-varying)		
Strategy switching	**-0.418 [-0.717, -0.118]**	**-0.334 [-0.615, -0.054]**
Endorsement change	**-0.545 [-0.768, -0.321]**	**-0.267 [-0.470, -0.064]**
Endorsement change, moderated by within-dataset age difference	0.009 [-0.075, 0.092]	-0.058 [-0.117, 0.001]
Strategy switching, moderated by within-dataset age difference	-0.024 [-0.106, 0.058]	-0.002 [-0.050, 0.046]
Lagged emotion differentiation	**-0.019 [-0.031, -0.007]**	0.030 [-0.002, 0.061]
Emotion intensity	**-3.927 [-4.989, -2.865]**	**0.520 [0.196, 0.845]**
Emotion regulation intensity	-0.036 [-0.120, 0.048]	**-0.158 [-0.249, -0.067]**
Time trend	**-0.006 [-0.008, -0.004]**	**0.005 [0.003, 0.006]**
Between-person (time-invariant)		
Intercept	**-2.041 [-2.251, -1.832]**	**-1.754 [-2.246, -1.262]**
Strategy switching	0.055 [-0.008, 0.119]	-0.004 [-0.052, 0.044]
Endorsement change	**-0.092 [-0.141, -0.042]**	-0.018 [-0.054, 0.019]
Emotion intensity	**-0.238 [-0.295, -0.180]**	**0.034 [0.004, 0.064]**
Emotion regulation intensity	**-0.068 [-0.114, -0.022]**	-0.016 [-0.048, 0.016]
Within-dataset age difference	-0.034 [-0.085, 0.017]	**-0.077 [-0.116, -0.038]**
Gender (female = 1, male = 0)	0.034 [-0.086, 0.154]	**-0.151 [-0.241, -0.061]**

Note: Significant effects are displayed in bold. n: number of ESM assessments; N: number of adolescents; b: unstandardized effect; CI: confidence interval. In Model 1M, n is doubled because of how data have undergone the stacking preparation step.

References

Achterhof, R., Kirtley, O. J., Schneider, M., Hagemann, N., Hermans, K. S., Hiekkaranta, A. P., Lecei, A., Lafit, G., & Myin-Germeys, I. (2022). Adolescents' real-time social and affective experiences of online and face-to-face interactions. *Computers in Human Behavior*, *129*, 107159.

Aldao, A., Nolen-Hoeksema, S., & Schweizer, S. (2010). Emotion-regulation strategies across psychopathology: A meta-analytic review. *Clinical Psychology Review*, *30*(2), 217–237.

Bakker, J. M., Goossens, L., Kumar, P., Lange, I. M., Michielse, S., Schruers, K., Bastiaansen, J. A., Lieverse, R., Marcelis, M., van Amelsvoort, T.others. (2019). From laboratory to life: Associating brain reward processing with real-life motivated behaviour and symptoms of depression in non-help-seeking young adults. *Psychological Medicine*, *49*(14), 2441–2451.

Barge-Schaapveld, D. Q., Nicolson, N. A., Berkhof, J., & Marten, W. deVries. (1999). Quality of life in depression: Daily life determinants and variability. *Psychiatry Research*, *88*(3), 173–189.

Barrantes-Vidal, N., Chun, C., Myin-Germeys, I., & Kwapil, T. (2013). Psychometric Schizotypy Predicts Psychotic-Like, Paranoid, and Negative Symptoms in Daily Life. *Journal of Abnormal Psychology*, *122*, 1077–1087. https://doi.org/10.1037/a0034793

Bastiaansen, J. A., Meurs, M., Stelwagen, R., Wunderink, L., Schoevers, R. A., Wichers, M., & Oldehinkel, A. J. (2018). Self-monitoring and personalized feedback based on the experiencing sampling method as a tool to boost depression treatment: A protocol of a pragmatic randomized controlled trial (ZELF-i). *BMC Psychiatry*, *18*, 1–11.

Bates, D., Kliegl, R., Vasishth, S., & Baayen, H. (2015). Parsimonious mixed models. *arXiv Preprint arXiv:1506.04967*. https://arxiv.org/abs/1506.04967

Bates, D., Mächler, M., Bolker, B., & Walker, S. (2015). Fitting linear mixed-effects models using lme4. *Journal of Statistical Software*, *67*(1), 1–48.

https://doi.org/10.18637/jss.v067.i01

Bauer, D. J., Preacher, K. J., & Gil, K. M. (2006). Conceptualizing and testing random indirect effects and moderated mediation in multilevel models: New procedures and recommendations. *Psychological Methods*, *11*(2), 142–163. https://doi.org/10.1037/1082-989X.11.2.142

Bennik, E. (2015). *Every dark cloud has a colored lining: The relation between positive and negative affect and reactivity to positive and negative events.*

Berking, M., & Znoj, H. (2011). *SEK-27-Fragebogen zur standardisierten selbsteinschätzung emotionaler kompetenzen.*

Bolger, N., & Laurenceau, J.-P. (2013). *Intensive longitudinal methods: An introduction to diary and experience sampling research.* Guilford press.

Braet, J., Debra, G., & Giletta, M. (2023). *I've got a friend in me: The effect of self-compassion on depressive symptoms via emotion regulation.*

Brans, K., Koval, P., Verduyn, P., Lim, Y. L., & Kuppens, P. (2013). The regulation of negative and positive affect in daily life. *Emotion*, *13*(5), 926–939. https://doi.org/10.1037/a0032400

Bülow, A., van Roekel, E., Boele, S., Denissen, J. J., & Keijsers, L. (2022). Parent–adolescent interaction quality and adolescent affect—An experience sampling study on effect heterogeneity. *Child Development*, *93*(3), e315–e331.

Delespaul, P., & DeVries, M. W. (1987). The daily life of ambulatory chronic mental patients. *The Journal of Nervous and Mental Disease*, *175*(9), 537–544.

Eisele, G., Lafit, G., Vachon, H., Kuppens, P., Houben, M., Myin-Germeys, I., & Viechtbauer, W. (2021). Affective structure, measurement invariance, and reliability across different experience sampling protocols. *Journal of Research in Personality*, *92*, 104094. https://doi.org/10.1016/j.jrp.2021.104094

Emery, N. N., Walters, K. J., Njeim, L., Barr, M., Gelman, D., & Eddie, D. (2022). Emotion differentiation in early recovery from alcohol use disorder: Associations with

in-the-moment affect and 3-month drinking outcomes. *Alcoholism: Clinical and Experimental Research*, *46*(7), 1294–1305.

Erbas, Y., Ceulemans, E., Kalokerinos, E. K., Houben, M., Koval, P., Pe, M. L., & Kuppens, P. (2018). Why I don't always know what I'm feeling: The role of stress in within-person fluctuations in emotion differentiation. *Journal of Personality and Social Psychology*, *115*(2), 179.

Erbas, Y., Kalokerinos, E. K., Kuppens, P., van Halem, S., & Ceulemans, E. (2021). Momentary Emotion Differentiation: The Derivation and Validation of an index to Study Within-Person Fluctuations in Emotion Differentiation. *Assessment*, 107319112199008. https://doi.org/10.1177/1073191121990089

Fried, E. I., Papanikolaou, F., & Epskamp, S. (2022). Mental health and social contact during the COVID-19 pandemic: An ecological momentary assessment study. *Clinical Psychological Science*, *10*(2), 340–354.

Hartley, S., Haddock, G., e Sa, D. V., Emsley, R., & Barrowclough, C. (2014). An experience sampling study of worry and rumination in psychosis. *Psychological Medicine*, *44*(8), 1605–1614.

Hasmi, L., Drukker, M., Guloksuz, S., Menne-Lothmann, C., Decoster, J., Van Winkel, R., Collip, D., Delespaul, P., De Hert, M., Derom, C.others. (2017). Network approach to understanding emotion dynamics in relation to childhood trauma and genetic liability to psychopathology: Replication of a prospective experience sampling analysis. *Frontiers in Psychology*, *8*, 1908.

Jacobs, N., Myin-Germeys, I., Derom, C., Delespaul, P., Van Os, J., & Nicolson, N. (2007). A momentary assessment study of the relationship between affective and adrenocortical stress responses in daily life. *Biological Psychology*, *74*(1), 60–66.

Kiekens, G., Claes, L., Schoefs, S., Kemme, N. D., Luyckx, K., Kleiman, E. M., Nock, M. K., Myin-Germeys, I.others. (2023). The detection of acute risk of self-injury project: Protocol for an ecological momentary assessment study among individuals seeking

treatment. *JMIR Research Protocols*, *12*(1), e46244.

Knapp, K. S., Bradizza, C. M., Zhao, J., Linn, B. K., Wilding, G. E., LaBarre, C., & Stasiewicz, P. R. (2024). Emotion differentiation among individuals in a randomized clinical trial for alcohol use disorder: Within-and between-person associations with affect, craving, and alcohol use in daily life. *Behaviour Research and Therapy*, *173*, 104474.

Koval, P., Pe, M. L., Meers, K., & Kuppens, P. (2013). Affect dynamics in relation to depressive symptoms: Variable, unstable or inert? *Emotion (Washington, D.C.)*, *13*(6), 1132.

Lafit, G., Adolf, J. K., Dejonckheere, E., Myin-Germeys, I., Viechtbauer, W., & Ceulemans, E. (2021). Selection of the Number of Participants in Intensive Longitudinal Studies: A User-Friendly Shiny App and Tutorial for Performing Power Analysis in Multilevel Regression Models That Account for Temporal Dependencies. *Advances in Methods and Practices in Psychological Science*, *4*(1), 251524592097873. https://doi.org/10.1177/2515245920978738

Lischetzke, T., Schemer, L., Glombiewski, J. A., In-Albon, T., Karbach, J., & Könen, T. (2021). Negative Emotion Differentiation Attenuates the Within-Person Indirect Effect of Daily Stress on Nightly Sleep Quality Through Calmness. *Frontiers in Psychology*, *12*, 684117. https://doi.org/10.3389/fpsyg.2021.684117

Lo, T. T., van Lissa, C. J., Verhagen, M., Hoemann, K., Erbas, Y., & Maciejewski, D. (2024). A theory-informed emotion regulation variability index: Bray-curtis dissimilarity. *Emotion*. https://doi.org/10.1037/emo0001344

McNeish, D., & MacKinnon, D. P. (2022). Intensive longitudinal mediation in Mplus. *Psychological Methods*.

Medland, H., De France, K., Hollenstein, T., Mussoff, D., & Koval, P. (2020). Regulating Emotion Systems in Everyday Life: Reliability and Validity of the RESS-EMA Scale. *European Journal of Psychological Assessment*, *36*(3), 437–446.

https://doi.org/10.1027/1015-5759/a000595

Mestdagh, M., Verdonck, S., Piot, M., Niemeijer, K., Kilani, G., Tuerlinckx, F., Kuppens, P., & Dejonckheere, E. (2023). M-Path: An easy-to-use and highly tailorable platform for ecological momentary assessment and intervention in behavioral research and clinical practice. *Frontiers in Digital Health*, *5*, 1182175.

Mosannenzadeh, F. (2021). *Attachment and emotions in romantic relationships [pre-registration]*. https://doi.org/10.17605/OSF.IO/M2T9G

Myin-Germeys, I., Delespaul, P. A., & DeVries, M. W. (2000). Schizophrenia patients are more emotionally active than is assumed based on their behavior. *Schizophrenia Bulletin*, *26*(4), 847–854.

O'Brien, S. T., Hinton, J. D., Moeck, E., Susanto, R., Jayaputera, G., Sinnott, R., Vu, D., Alvarez, M., Gleeson, J., & Koval, P. (2023). *SEMA3: A free smartphone platform for daily life surveys.*

Parkinson, B., & Totterdell, P. (1999). Classifying affect-regulation strategies. *Cognition & Emotion*, *13*(3), 277–303.

Preacher, K., & Selig, J. (2010). *Monte Carlo method for assessing multilevel mediation: An interactive tool for creating confidence intervals for indirect effects in 1-1-1 multilevel models [Computer software].*

Radloff, L. S. (1977). The CES-D scale: A self-report depression scale for research in the general population. *Applied Psychological Measurement*, *1*(3), 385–401.

Ram, N. (2017). *Analysis of Experience Sampling & EMA Data - Chapter 7: Within-Person (1-1-1) Mediation.* QuantDev, Pennsylvania State University.

Ram, N. (2022). *Specifying 1-1-1 mediation models in R.* The Change Lab, Stanford University.

Rauschenberg, C., van Os, J., Cremers, D., Goedhart, M., Schieveld, J. N., & Reininghaus, U. (2017). Stress sensitivity as a putative mechanism linking childhood trauma and psychopathology in youth's daily life. *Acta Psychiatrica Scandinavica*, *136*(4), 373–388.

Sawyer, S. M., Azzopardi, P. S., Wickremarathne, D., & Patton, G. C. (2018). The age of adolescence. *The Lancet Child & Adolescent Health*, *2*(3), 223–228.

Schermelleh-Engel, K., Moosbrugger, H., Müller, H.others. (2003). Evaluating the fit of structural equation models: Tests of significance and descriptive goodness-of-fit measures. *Methods of Psychological Research Online*, *8*(2), 23–74.

Schneiders, J., Nicolson, N. A., Berkhof, J., Feron, F. J., Van Os, J., & Devries, M. W. (2006). Mood reactivity to daily negative events in early adolescence: Relationship to risk for psychopathology. *Developmental Psychology*, *42*(3), 543.

Spence, J. R., Brown, D. J., Keeping, L. M., & Lian, H. (2014). Helpful today, but not tomorrow? Feeling grateful as a predictor of daily organizational citizenship behaviors. *Personnel Psychology*, *67*(3), 705–738.

Springstein, T., Thompson, R. J., & English, T. (2023). Examining situational differences in momentary emotion differentiation and emotional clarity in everyday life. *Emotion (Washington, D.C.)*.

van den Broek, N., Larsen, Junilla. K., Verhagen, M., Burk, W. J., & Vink, J. M. (2020). Is Adolescents' Food Intake Associated with Exposure to the Food Intake of Their Mothers and Best Friends? *Nutrients*, *12*(3), 786. https://doi.org/10.3390/nu12030786

van Eck, M., Nicolson, N. A., & Berkhof, J. (1998). Effects of stressful daily events on mood states: Relationship to global perceived stress. *Journal of Personality and Social Psychology*, *75*(6), 1572.

Van Roekel, E., & Trompetter, H. (2023). *Understanding (individual differences in) emotion regulation in daily life.* https://osf.io/7q4gd/.

Verhagen, M., Lo, T. T., Maciejewski, D. F., & Eltanamly, H. (2022). *Flits Study: A dyadic (parent-adolescent) EMA design [dataset].*

Vogelsmeier, L. V., Jongerling, J., & Maassen, E. (2023). *Assessing and accounting for measurement in intensive longitudinal studies: Current practices, considerations, and avenues for improvement.*

von Klipstein, L., Servaas, M. N., Lamers, F., Schoevers, R. A., Wardenaar, K. J., & Riese, H. (2023). Increased affective reactivity among depressed individuals can be explained by floor effects: An experience sampling study. *Journal of Affective Disorders*, *334*, 370–381.
